# A single-amino-acid cleavage controls global mitochondrial complex integrity

**DOI:** 10.1038/s41594-026-01876-7

**Published:** 2026-08-31

**Authors:** Cansu Kücükköse, Marcin Luzarowski, Fabian Stockert, Annette Flotho, Miguel Cosenza-Contreras, Fatih Demir, Max Gilbert, Jörn Dengjel, Friedel Drepper, Mandy Jeske, Hans-Georg Koch, Pitter F. Huesgen, F.-Nora Vögtle

**Affiliations:** 1 Center for Molecular Biology of Heidelberg University (ZMBH), DKFZ-ZMBH Alliance, Heidelberg, Germany; 2 Core Facility for Mass Spectrometry and Proteomics, Center for Molecular Biology of Heidelberg University (ZMBH), DKFZ-ZMBH Alliance, Heidelberg, Germany; 3 Biochemistry and Functional Proteomics, Institute of Biology II, Faculty of Biology, University of Freiburg, Freiburg, Germany; 4 Spemann Graduate School of Biology and Medicine (SGBM), University of Freiburg, Freiburg, Germany; 5 Central Institute for Engineering, Electronics and Analytics, ZEA-3, Forschungszentrum Jülich, Jülich, Germany; 6 Department of Biology, University of Fribourg, Fribourg, Switzerland; 7 Heidelberg University Biochemistry Center (BZH), Heidelberg University, Heidelberg, Germany; 8 Institute of Biochemistry and Molecular Biology, ZBMZ, Faculty of Medicine, University of Freiburg, Freiburg, Germany; 9 CIBSS – Centre for Integrative Biological Signalling Studies, University of Freiburg, Freiburg, Germany; 10 Network Aging Research, Heidelberg University, Heidelberg, Germany

**Keywords:** Proteases, Mitochondrial proteins, Protein quality control, Post-translational modifications

## Abstract

Mitochondrial proteostasis depends on precise N-terminal processing of imported precursor proteins. Defects in this maturation step are implicated in disease, yet the functional impact in humans remains unclear. Here we show that the intermediate cleaving peptidase ICP55, which removes a single amino acid, acts as a key stabilizer of multimeric mitochondrial protein complexes. Using proteomics and complexome profiling, we identify over 100 human ICP55 substrates and demonstrate that loss of ICP55 triggers widespread destabilization of protein assemblies, with a global shift toward smaller subcomplexes. Thus, we uncover a conserved, post-translational mechanism that safeguards mitochondrial proteostasis by regulating complex integrity through a single amino-acid cleavage, and we reveal N-terminal proteoform control as an unexpected layer of organellar homeostasis.

## Abstract (web summary)

Kücükköse, Luzarowski and colleagues show that removing a single N-terminal amino acid during the maturation of newly imported mitochondrial proteins globally stabilizes mitochondrial protein complexes, revealing an unexpected layer of regulation of mitochondrial proteostasis.

## Main

Mitochondria are essential organelles involved in energy conversion, biosynthesis and signaling pathways, and their dysfunction underlies a broad spectrum of human diseases^1,2^. Most mitochondrial proteins are nuclear-encoded, synthesized in the cytosol, and imported into the organelle as precursors carrying N-terminal mitochondrial targeting signals (MTSs)^3–5^. These presequences are proteolytically removed upon entry into the matrix in a sequential process: the mitochondrial processing peptidase (MPP) cleaves the presequence to expose a new N terminus, and in several cases, the intermediate cleaving peptidase Icp55 removes an additional single amino acid to generate a stabilizing N terminus^3,6–9^. The mitochondrial intermediate peptidase (MIP, initially identified in rat^10^; Oct1 in yeast^11^) performs a similar secondary trimming function for a different subset of substrates, removing an additional eight amino acids from MPP-generated processing intermediates^12,13^. Importantly, sequential processing is defined largely from studies in yeast, and its relevance in human mitochondria remains unclear. Mutations in the MPP subunits PMPCA and PMPCB, MIP and ICP55 (encoded by *XPNPEP3*) have been identified in patients with severe neurodegeneration, cardiomyopathy and kidney disease, underscoring the physiological importance of precise presequence processing^14–17^. However, despite this clear clinical relevance, the molecular consequences of defective N-terminal processing and trimming in human mitochondria remain poorly understood.

In *Saccharomyces cerevisiae* ~70% of mitochondrial proteins carry cleavable presequences^3^. Icp55 substrates share the common MPP cleavage site motif (arginine or lysine in position –2 of the MPP cleavage site) followed by a bulky hydrophobic residue (tyrosine, leucine or phenylalanine) at the newly exposed N terminus that is removed by Icp55. The Icp55 substrate spectrum is well characterized in yeast, but its substrate specificity and functional relevance in human mitochondria are unknown. Current computational predictions for human presequences as well as for ICP55 substrates are based on algorithms trained mainly on yeast and plant data, raising the likelihood that human-specific cleavage site motifs and ICP55 substrates remain undiscovered.

In this Article we perform high-resolution N-terminal proteomics comparing HEK293T wild type (WT) with ICP55 knockout cells to determine authentic mitochondrial cleavage sites and define the ICP55 substrate repertoire. We uncover previously unrecognized ICP55 cleavage motifs and assess the proteome-wide consequences of impaired N-terminal processing on mitochondrial function.

## Results

### Global N-terminomics identifies the ICP55 substrate spectrum and additional cleavage motifs

**Extended Data Fig. 1:**
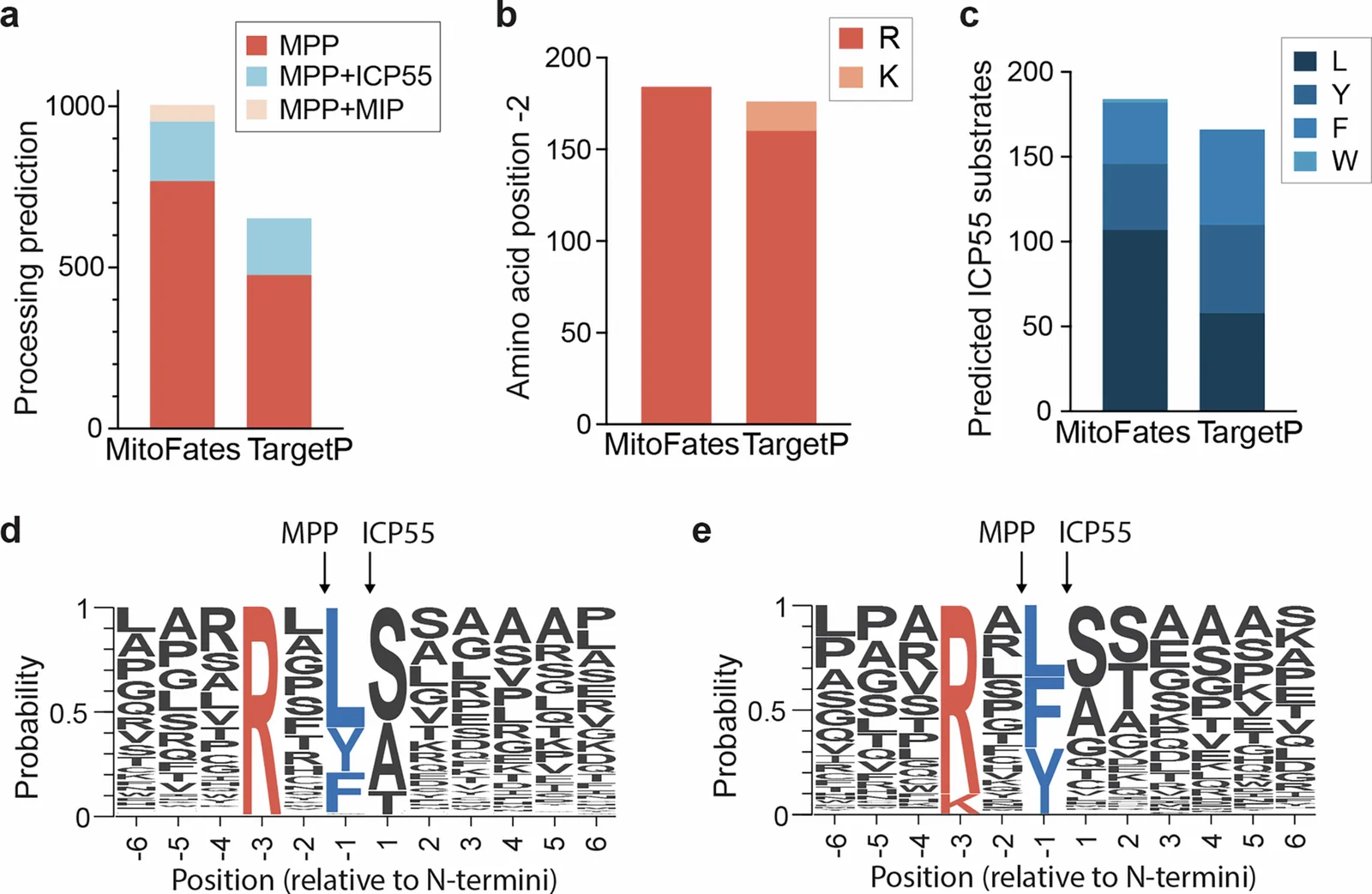
Prediction of mitochondrial precursor cleavage by MitoFates and TargetP. **a**) Prediction of MPP, MPP-ICP55 and MPP-MIP substrates by MitoFates and TargetP using the human proteome (Supplementary Table 1). Threshold for prediction > 0.385. **b**) Categorization of predicted ICP55 substrates in R-2 and K-2 MPP cleavage motifs. **c**) Categorization of predicted ICP55 substrates into F, Y, L, or W ICP55 cleavage motifs. **d**) Amino acid plot of relative frequency for ICP55 substrates predicted by MitoFates. **e**) Amino acid plot of relative frequency for ICP55 substrates predicted by TargetP. Source data

Mitochondrial precursor processing represents the crucial maturation step following the import of nuclear-encoded proteins into mitochondria. Whereas the majority of mitochondrial proteins use cleavable N-terminal presequences as targeting signals for import in yeast^3^, comprehensive, high-confidence data for the human mitochondrial proteome are lacking. To gain initial insights into the landscape of presequence-containing proteins in human cells, we subjected the complete human proteome to two widely used mitochondrial targeting prediction algorithms, MitoFates and TargetP^18,19^ (Extended Data Fig. 1a and Supplementary Table 1). MitoFates predicted MTSs in 1,004 proteins, whereas TargetP identified cleavable presequences in only 656 proteins, corresponding to 60–90% of mitochondrial proteins and highlighting substantial divergence between the algorithms.

To probe for potential substrates of the human orthologue of yeast ICP55, we searched for the established MPP-ICP55 cleavage motifs derived from yeast^3,6^ and plant datasets^7,8^ (characterized by an MPP recognition site with R or K at position –2, followed by a bulky hydrophobic residue—Y, L or F—removed by Icp55). Using this criterion, we identified 184 (MitoFates, which directly predicts Icp55 substrates) and 166 (TargetP) putative ICP55 substrates in human mitochondria (Extended Data Fig. 1b,c). Motif visualization based on relative amino-acid frequencies at the predicted cleavage sites revealed a clear enrichment for R at the –3 position, followed by the predicted single-residue cleavage by ICP55 (Extended Data Fig. 1d,e).

**Fig. 1:**
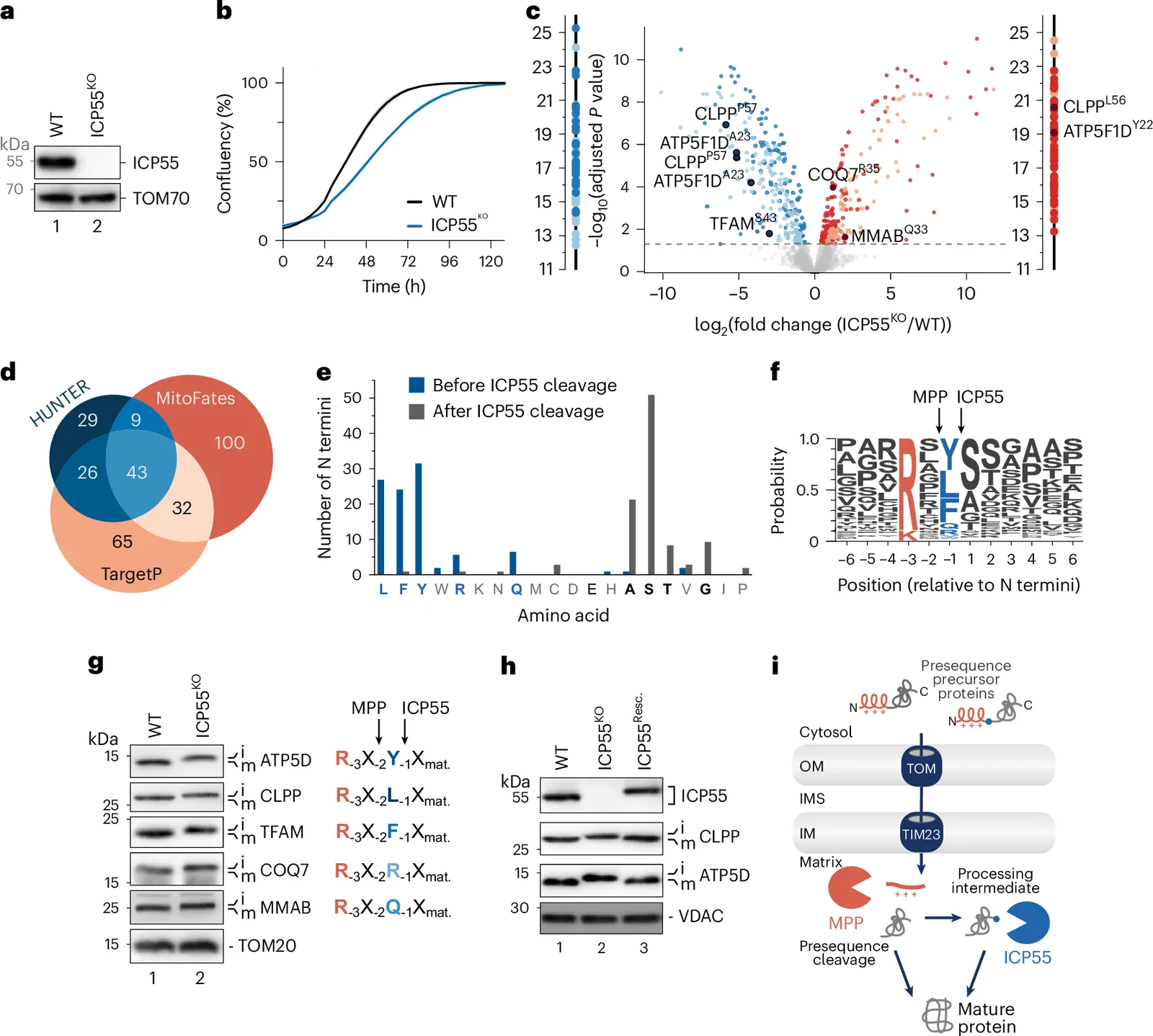
Identification of the ICP55 substrate repertoire using an unbiased global proteomic approach. **a**, Analysis of protein levels in mitochondria isolated from HEK293T WT and ICP55^KO^ cells. Samples were subjected to SDS–PAGE and immunoblotting. **b**, Assessment of proliferation of WT and ICP55^KO^ cells on high-glucose medium. Data are mean ± s.e.m. from three replicates. Shaded areas indicate s.e.m. **c**, N-terminal peptide abundance in WT and ICP55^KO^ proteomes visualized as a volcano plot (Supplementary Table 2). Shown are mean log_2_ ratios (ICP55^KO^/WT) of 3,042 peptides quantified in at least three of five replicates in both conditions. Peptides with significant differences in abundance (FDR-controlled LIMMA-moderated two-sided *t*-test with *P* < 0.05) are colored. Dark red and dark blue indicate N-terminal peptides identified after digestion with trypsin, and light red and light blue after digestion with legumain. 159 peptides only observed in one of the two genotypes are indicated on log_2_-transformed intensity scales on either side of the volcano plot. Selected N termini are highlighted with the start position in the protein. **d**, Venn diagram comparing experimentally determined ICP55 substrates (HUNTER; Supplementary Table 3) with predictions from MitoFates and TargetP. Numbers indicate individual proteins. **e**, Distribution of amino acids at N termini from experimentally identified ICP55 substrates in ICP55^KO^ (before ICP55 cleavage) and WT (after ICP55 cleavage) mitochondria. **f**, Relative frequency of amino acids in experimentally determined ICP55 substrates. The first six amino acids before and after the ICP55 cleavage site are shown. **g**, Analysis of migration of experimentally determined ICP55 substrates from each substrate class. Mitochondria isolated from WT and ICP55^KO^ cells were analyzed by SDS–PAGE and immunoblotting. TOM20, loading control; i, processing intermediate; m, mature protein. Representative immunoblots of three independent experiments are shown. **h**, Analysis of protein levels and migration comparing mitochondria from WT, ICP55^KO^ and ICP55^KO^ cells re-expressing ICP55 (ICP55^Resc.^). Samples were analyzed by SDS–PAGE and immunoblotting. VDAC, loading control. Representative immunoblots of three independent experiments are shown. **i**, Cartoon illustrating the presequence import pathway in human cells including presequence processing by MPP and ICP55. OM, outer membrane; IMS, intermembrane space; IM, inner membrane. Source data

**Extended Data Fig. 2:**
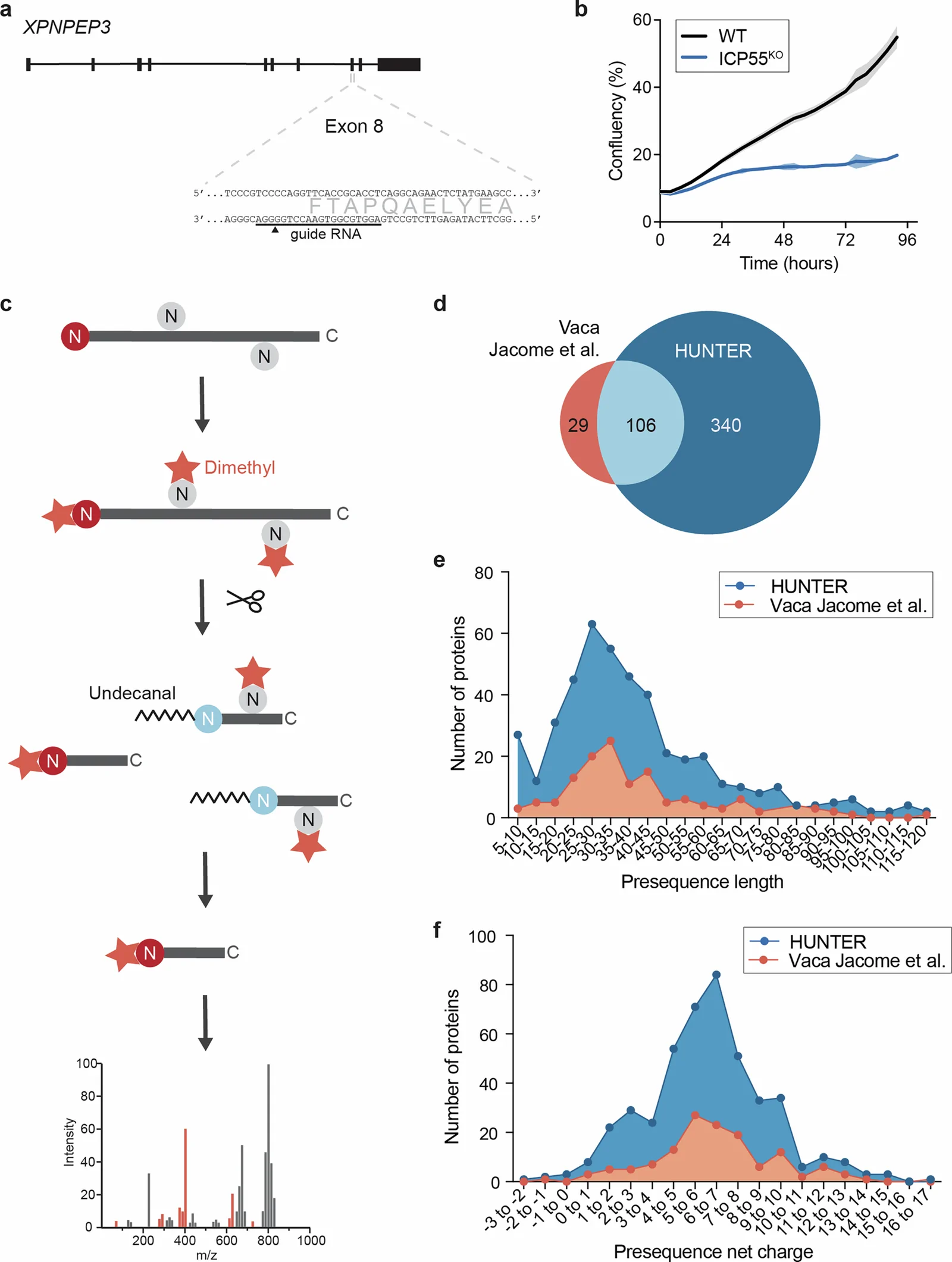
ICP55^KO^ generation and experimental N-terminomics strategy. **a**) Schematic overview of the targeting strategy of human ICP55 by CRISPR-Cas9. The guide RNA was directed against exon 8 of *XPNPEP3*. Successful targeting of the gene was confirmed by sequencing. **b**) Proliferation assay of wild-type (WT) and ICP55^KO^ cells on low-glucose medium. Data represent mean ± SEM from three replicates. Shaded areas indicate SEM. **c**) Cartoon depicting the quantitative N-proteomics analysis using HUNTER (High-efficiency Undecanal-based N-Termini EnRichment). **d**) Comparison of protein N-termini identified by HUNTER (Supplementary Table 2) and previous N-terminome analysis of U937 cells (Vaca Jacome et al.)^22^. **e**) Analysis of presequence length of processed mitochondrial proteins identified by HUNTER (446) and in Vaca Jacome et al. (135)^22^. **f**) Analysis of presequence net charge of cleaved proteins identified by HUNTER and in Vaca Jacome et al.^22^. Source data

Although prediction tools provide valuable initial insights into the potential substrate spectrum and subcellular targeting, they are inherently limited by species-specific training sets and their reliance on known motifs, rendering them unable to identify previously unrecognized cleavage sites. To obtain a comprehensive and unbiased view of the authentic substrate repertoire of human ICP55, we generated ICP55 knockout (ICP55^KO^) HEK293T cells using CRISPR-Cas9-mediated genome editing (Fig. 1a and Extended Data Fig. 2a). Loss of ICP55 led to a growth defect under high-glucose growth conditions, which was markedly exacerbated under low-glucose conditions (Fig. 1b and Extended Data Fig. 2b). We isolated mitochondria and analyzed their N termini globally by high-efficiency undecanal-based N-termini enrichment (HUNTER) proteomics (Fig. 1c, Extended Data Fig. 2c and Supplementary Table 2)^20^. Across all samples, this approach identified 3,201 N-terminal peptides corresponding to 1,511 proteins. Among these, 446 mitochondrial proteins with cleavable N termini (Methods; starting at residues 5–120 and identified in at least three out of five WT replicates)^21^.

Next, we compared our N-terminome to a previously published study by Vaca Jacome et al.^22^, which identified N termini for 135 processed mitochondrial proteins in U937 cells (up to residue 120) using doublet N-terminal oriented proteomics (dN-TOP). Of these, 106 overlapped with the 446 proteins identified in our HUNTER dataset (Extended Data Fig. 2d; using a criterion of single-peptide identification in the Vaca Jacome dataset and peptide presence in ≥3 of 5 replicates in our dataset). Analysis of the presequence length distribution confirmed the expected average length of 20–40 amino acids (Extended Data Fig. 2e), and revealed that net positive charges of +4 to +8 were most prevalent (Extended Data Fig. 2f), consistent with findings in yeast and plant MTSs^3,7,8^. The comparisons collectively validate our human mitochondrial N-terminome as a valuable resource for studying mitochondrial protein maturation and processing mechanisms.

**Extended Data Fig. 3:**
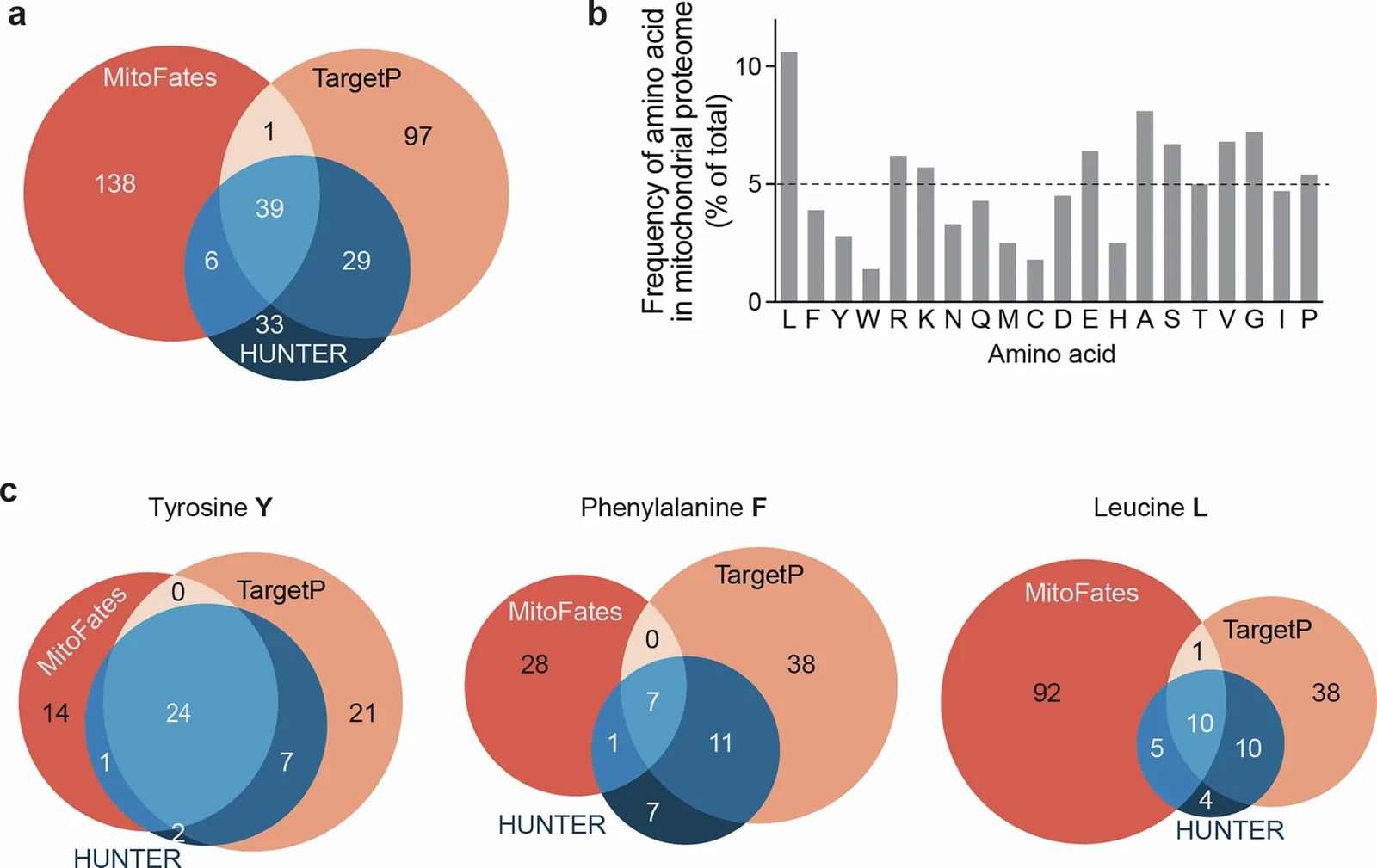
Comparison of experimental and *in silico* ICP55 cleavage motifs. **a**) Venn diagram comparing experimentally determined ICP55 substrates (HUNTER; Supplementary Table 3) with prediction from MitoFates and TargetP (Supplementary Table 1). Numbers indicate identified or predicted peptides. **b**) Frequency of all amino acids in the human mitochondrial proteome^21^. Dashed horizontal line, average amino acid frequency. **c**) Individual comparison of Y, F and L substrates from experimentally determined ICP55 substrates with MitoFates and TargetP predictions. Source data

We next examined our dataset for evidence of single-residue cleavage events indicative of ICP55 activity following initial processing by MPP. We identified 107 proteins that, in WT mitochondria, displayed typically two forms—an intermediate and a mature N terminus—differing by a single residue, with the extended form predominantly detected in ICP55^KO^ mitochondria. Among the 107 putative ICP55 substrates, 43 were predicted by both MitoFates and TargetP, while 9 and 26 were predicted exclusively by MitoFates and TargetP, respectively; notably, 29 substrates were not predicted by either algorithm (Fig. 1d, Extended Data Fig. 3a and Supplementary Table 3). Unexpectedly, analysis of these 29 unpredicted substrates revealed two additional categories of cleavage specificity beyond the canonical Y, L and F residues. R and Q also emerged as substrate-defining residues for ICP55 cleavage (Fig. 1e,f and Extended Data Fig. 3b), each supported by the presence of at least three proteins per additional motif class. Comparison of our experimentally defined ICP55 substrates with in silico predictions showed good agreement for Y-terminal substrates but poorer overlap for L- and F-motifs (Extended Data Fig. 3c), likely due to the yeast-derived training set that identified Y as the most frequently removed residue^3^.

To further validate our proteomic findings, we first assessed the sub-mitochondrial localization of the identified ICP55 substrates. Annotation based on MitoCarta3.0^21^ revealed that all experimentally identified substrates localize to the matrix or inner membrane (IM), consistent with their accessibility to MPP- and ICP55-mediated processing (Supplementary Table 3). We next evaluated the electrophoretic mobility of representative ICP55 substrates from each cleavage class in WT and ICP55^KO^ mitochondria by sodium dodecyl sulfate polyacrylamide gel electrophoresis (SDS–PAGE) and immunoblotting (Fig. 1g). The identified ICP55 substrates exhibited slight shifts in mobility in ICP55^KO^ mitochondria that were restored upon re-expression of ICP55, confirming the cleavage defect was attributable to loss of ICP55 (Fig. 1h).

Collectively, our comprehensive proteomic strategy identifies mature N termini for 446 processed human mitochondrial proteins, substantially expanding current datasets. Quantitative N-terminal analysis of WT and ICP55^KO^ mitochondria identified 107 high-confidence ICP55 substrates and uncovered additional cleavage specificities for the human protease. Integration of experimental and predicted data suggests that up to 300 of the ~1,100 mitochondrial proteins in human cells may be subject to dual processing by MPP and ICP55, representing an upper-bound estimate based on our N-terminome data and prediction algorithms (Fig. 1i). Thus, our findings substantially extend the so far best characterized ICP55 substrate spectrum from yeast mitochondria, which is currently limited to ~50 proteins, and indicate that sequential N-terminal processing may be more prevalent in human mitochondria than previously anticipated.

### Retention of a single N-terminal residue disrupts respiratory chain complex integrity

**Fig. 2:**
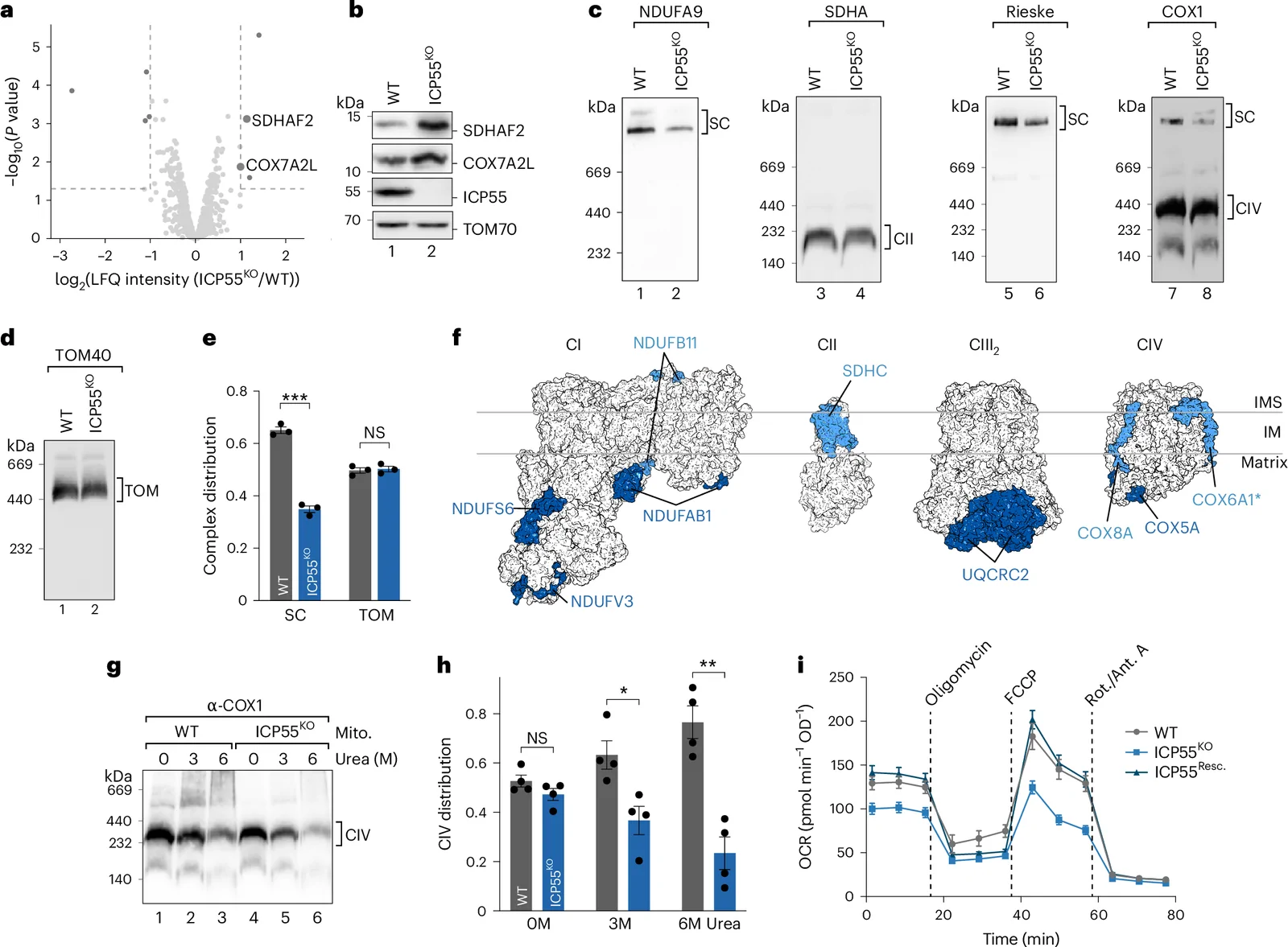
Loss of ICP55 impacts respiratory chain integrity via destabilization of protein-protein interactions. **a**, Volcano plot visualizing changes in mitochondrial protein abundance in ICP55^KO^ compared to WT cells (Supplementary Table 4). *n* = 5 biological replicates. Dashed lines indicate the chosen significance thresholds (horizontal line, LIMMA-moderated two-sided Student’s *t*-test FDR-adjusted *P* = 0.05; left and right vertical lines, twofold decrease or increase in abundance, respectively). **b**, Analysis of protein levels of mitochondria isolated from WT and ICP55^KO^ cells. Samples were subjected to SDS–PAGE and immunoblotting. Representative immunoblots of three independent experiments are shown. **c**,**d**, Isolated mitochondria from WT and ICP55^KO^ cells were solubilized in digitonin buffer and analyzed by BN–PAGE and immunoblotting (NDUFA9, SDHA, Rieske, COX1 (**c**); TOM40 (**d**)). SC, respiratory chain supercomplexes; CII, respiratory chain complex II; CIV, respiratory chain complex IV; TOM, translocase of the outer mitochondrial membrane. Representative immunoblots of three independent experiments are shown. **e**, Quantification of the respiratory chain supercomplex and TOM complex from the analysis in **c** and **d** using antisera against Rieske (CIII subunit) and TOM40. Data represent mean ± s.e.m. from three replicates. Unpaired, two-sided Student’s *t*-test was used to compare between two groups. Significances are indicated with asterisks: ***P < 0.001; NS, not significant. Exact *P* values: SC 0.000072 and TOM 0.718. **f**, Structural visualization of ETC complexes: CI, PDB 6ZKC ref. ^51^; CII, PDB 1ZOY ref. ^52^; CIII_2_, PDB 6Q9E ref. ^53^; CIV, PDB 5Z62 ref. ^54^. Dark blue, experimentally determined ICP55 substrates; light blue, predicted ICP55 substrates. *The predicted ICP55 substrate COX6A2 (tissue-specific isoform) is mapped to the position of the ubiquitously expressed isoform COX6A1 in the structure. **g**, Determination of ETC complex IV stability by addition of 3 and 6 M urea during digitonin solubilization. Samples were analyzed as in **c**. **h**, Quantification of samples from **g**. Data represent mean ± s.e.m. from four replicates. Unpaired, two-sided Student’s *t*-test was used to compare between two groups. *P < 0.05; **P < 0.01. Exact *P* values: 0 M urea, *P* = 0.192; 3 M urea *P* = 0.017; 6 M urea, *P* = 0.001. **i**, Analysis of oxygen consumption rates (OCRs) of WT, ICP55^KO^ and ICP55^KO^ cells re-expressing ICP55 (ICP55^Resc.^) under basal conditions and after addition of the indicated compounds. Data represent mean ± s.e.m. from six replicates. Rot., rotenone; Ant. A, antimycin A; OD, optical density. Source data

To assess the functional consequences of ICP55 loss on mitochondrial physiology, we performed quantitative proteomic analysis comparing mitochondria from WT and ICP55^KO^ cells (Fig. 2a and Supplementary Table 4). Only a minor subset of proteins showed significant difference in abundance. Notably, this included two respiratory chain assembly factors, SDHAF2 and COX7A2L (also known as SCAF1), that accumulated in ICP55-deficient mitochondria. This increase was confirmed by immunoblotting, which revealed a pronounced increase in their steady-state levels (Fig. 2b). SDHAF2 has been implicated in the assembly of complexes II and IV of the electron transport chain (ETC), and COX7A2L is a known facilitator of respiratory chain supercomplex (SC) formation^23–25^. Importantly, neither SDHAF2 nor COX7A2L were identified as ICP55 substrates in our N-terminome (Supplementary Table 3).

**Extended Data Fig. 4:**
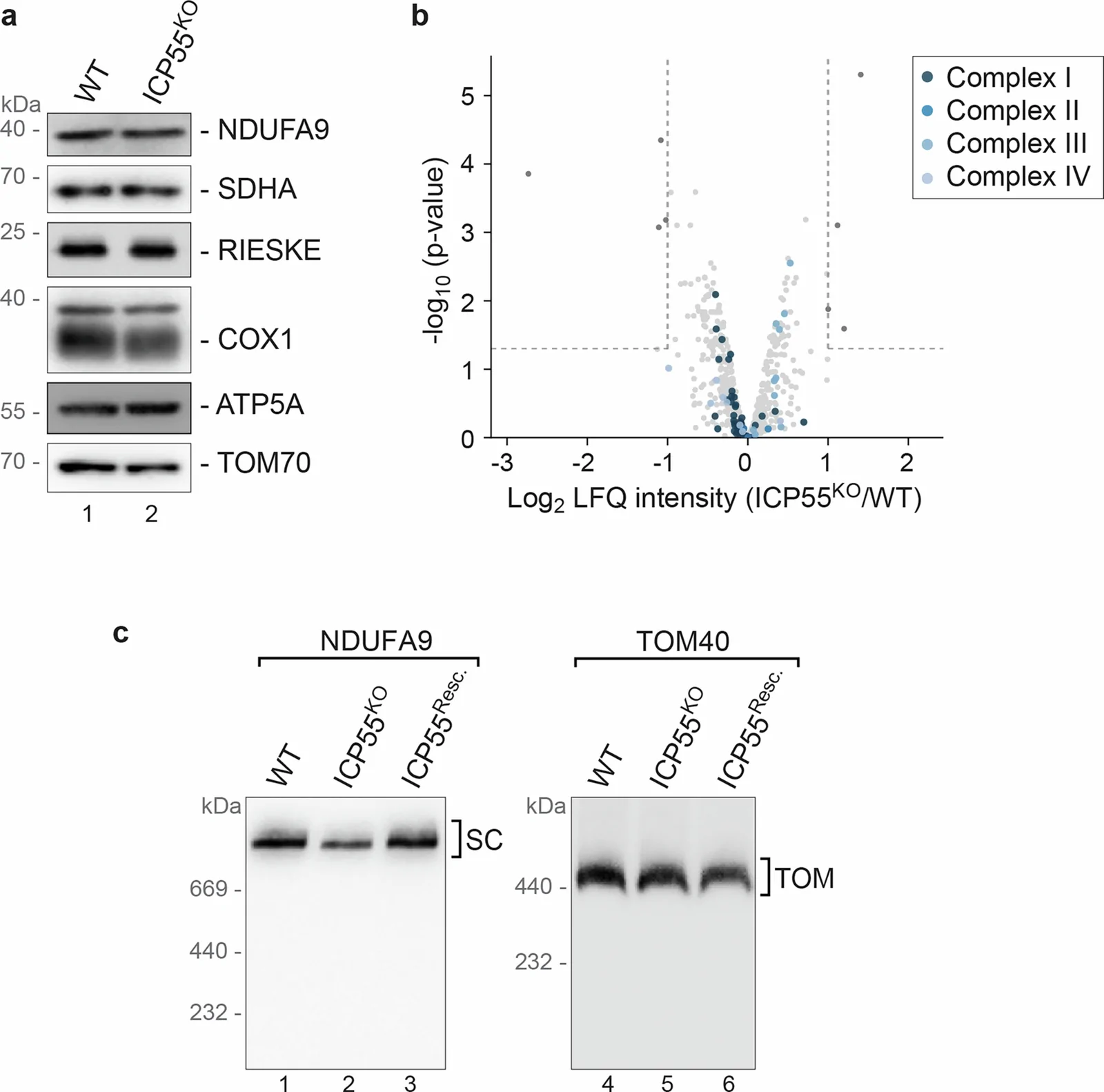
Loss of ICP55 does not change ETC subunit abundance and re-expression rescues supercomplex assembly. **a**) Analysis of protein levels in wild-type (WT) and ICP55^KO^ mitochondria. Samples were analyzed by SDS–PAGE and immunoblotting. **b**) Volcano plot of mitochondrial proteins quantified by proteomic analysis of WT and ICP55^KO^ cells (Supplementary Table 4). n = 5 replicates. Horizontal line, LIMMA-moderated two-sided Student´s t-test FDR-adjusted p-value = 0.05; left and right vertical lines, twofold decrease or increase in abundance, respectively. Subunits of electron transport chain complex I, II, III and IV are highlighted in blue. **c**) Isolated mitochondria from WT, ICP55^KO^ and ICP55^KO^ cells re-expressing ICP55 (ICP55^Resc.^) were solubilized in digitonin buffer and analyzed by BN–PAGE and immunoblotting. SC, respiratory chain supercomplexes; TOM40, loading control; TOM, translocase of the outer mitochondrial membrane. Representative immunoblots of three independent experiments. Source data

Given their role in ETC biogenesis, we hypothesized that upregulation of these assembly factors might reflect an increased formation of respiratory chain (super)complexes. To investigate this, we analyzed mitochondrial protein complexes by blue-native (BN)–PAGE followed by immunodetection (Fig. 2c–e). Surprisingly, despite the elevated levels of assembly factors, we observed a marked reduction in respiratory chain supercomplex abundance in ICP55^KO^ mitochondria. In contrast, the outer-membrane TOM complex remained unaffected. This reduction was not attributable to a decreased steady-state abundance of individual ETC subunits^26^, as these were unchanged or only mildly altered (Extended Data Fig. 4a,b). Importantly, re-expression of ICP55 restored respiratory chain supercomplex assembly, establishing that the observed defect is a direct consequence of ICP55 loss (Extended Data Fig. 4c).

Respiratory chain supercomplexes are primarily composed of complex I, dimeric complex III (III_2_) and complex IV, each of which includes subunits identified as ICP55 substrates (Fig. 2f). We speculated that the increased expression of assembly factors may reflect a compensatory response to instability of the ETC complexes. To directly assess ETC stability, we subjected mitochondria to digitonin solubilization in the presence or absence of urea. Urea destabilizes protein-protein interactions through direct binding to protein backbones and side chains, thereby weakening hydrogen bonds and van der Waals forces that stabilize native complexes. In parallel, urea alters water structure, reducing the hydrophobic effect that drives protein-folding and subunit association^27^. Together, these effects promote the unfolding and dissociation of protein assemblies^28–30^. Using this approach, we found that in addition to the reduced supercomplexes, the individual ETC complexes were more prone to urea-induced disassembly in ICP55^KO^ mitochondria than in WT controls (Fig. 2g,h). These findings suggest that the elongation of respiratory chain subunits by a single N-terminal amino acid, resulting from impaired ICP55-mediated processing, destabilizes protein-protein interfaces, impairing complex integrity.

To determine whether this decreased structural integrity impacts mitochondrial ETC function, we measured oxygen consumption rates. ICP55^KO^ cells exhibited a clear reduction in mitochondrial respiration (Fig. 2i). Importantly, re-expression of ICP55 restored respiratory activity to WT levels. Thus, the retention of a single N-terminal amino acid on multiple ETC subunits destabilizes respiratory chain assemblies, either by impairing supercomplex formation or decreasing mature complex stability, and diminishes mitochondrial respiratory activity.

### Loss of ICP55 decreases mitochondrial HSP60 complex stability

**Fig. 3:**
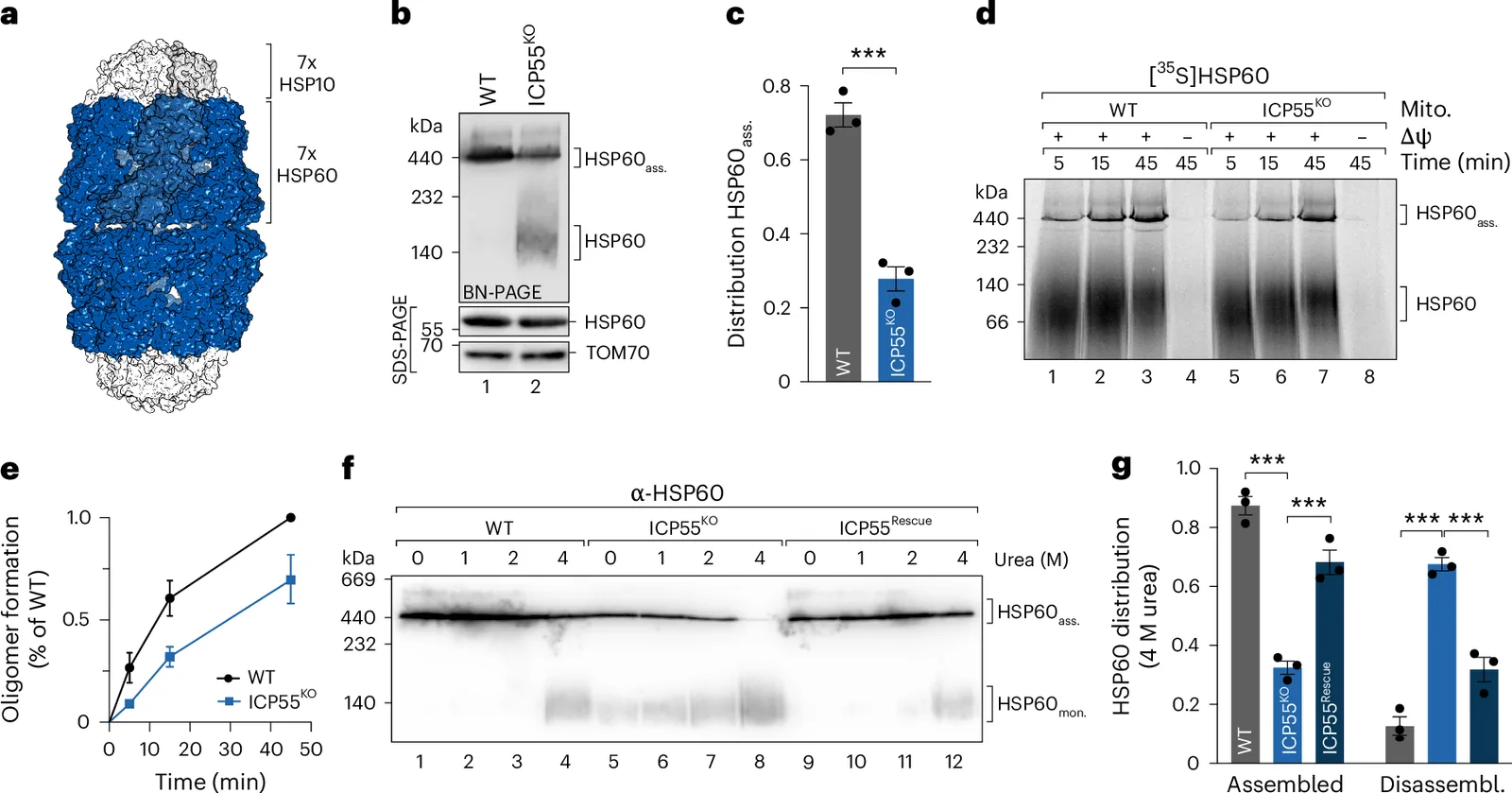
Impaired HSP60 complex formation in ICP55-deficient mitochondria. **a**, EM structure of the ATP-bound HSP60/HSP10 complex (PDB 9ES0)^55^. **b**, Analysis of protein levels in WT and ICP55^KO^ mitochondria. Samples were solubilized in digitonin buffer and analyzed on BN–PAGE (top panel) or subjected to SDS–PAGE (lower panels) followed by immunoblotting. HSP60_ass._, assembled HSP60 barrel. TOM70, loading control. Representative immunoblots of three independent experiments are shown. **c**, Quantification of assembled HSP60 from **b**. Data represent mean ± s.e.m. from three replicates. Unpaired, two-sided Student’s *t*-test was used to compare between two groups. Significances are indicated with asterisks: ***P < 0.001. Exact *P* value: *P* = 0.00073. **d**, Import of radiolabeled HSP60 precursor into mitochondria isolated from WT or ICP55^KO^ mitochondria. Where indicated, the membrane potential (Δ*ψ*) was dissipated before the import reaction. Samples were analyzed by BN–PAGE and autoradiography. Representative autoradiograph from three independent experiments. **e**, Quantification of the HSP60 oligomer formation kinetic shown in **d**. Data represent mean ± s.e.m. from three replicates. **f**, Isolated mitochondria from WT, ICP55^KO^ and ICP55^KO^ cells re-expressing ICP55 (ICP55^Rescue^) were solubilized in digitonin buffer in the absence or presence of different concentrations of urea and analyzed by BN–PAGE and immunoblotting. **g**, Quantification of assembled and disassembled HSP60 after treatment of isolated mitochondria with 4 M urea (analysis as in **f**). Data represent mean ± s.e.m. from three replicates. Statistical significance was assessed by one-way ANOVA followed by Tukey’s multiple-comparison test, performed separately for assembled and disassembled fractions. ***P < 0.001. Exact *P* values: WT:ICP55^KO^, *P* = 0.000053; ICP55^KO^:ICP55^Resc.^, *P* = 0.0006. Source data

The decreased integrity of the respiratory chain in ICP55^KO^ mitochondria led us to hypothesize that the retention of an additional N-terminal residue may destabilize other mitochondrial protein complexes that include ICP55 substrates. One such substrate is the mitochondrial chaperonin HSP60, the central folding machinery in the matrix. HSP60 functions as an essential protein-folding system in both prokaryotic and eukaryotic cells, forming a barrel-shaped monoheptameric complex that—together with HSP10—facilitates the refolding and maturation of imported or stress-denatured proteins within mitochondria (Fig. 3a)^31,32^. Its activity is vital for mitochondrial proteostasis and cellular viability, and mutations in HSP60 have been associated with neurodegenerative disease^33^.

To assess whether HSP60 complex integrity is compromised in the absence of ICP55, we examined the endogenous assembly state of HSP60 by BN–PAGE and immunoblotting. In ICP55^KO^ mitochondria, HSP60 complex formation was markedly decreased, despite unchanged steady-state levels of the protein (Fig. 3b). Notably, a lower molecular weight band appeared exclusively in ICP55-deficient mitochondria, likely representing monomeric HSP60 that failed to assemble into, or disintegrated from, the oligomeric barrel structure.

**Extended Data Fig. 5:**
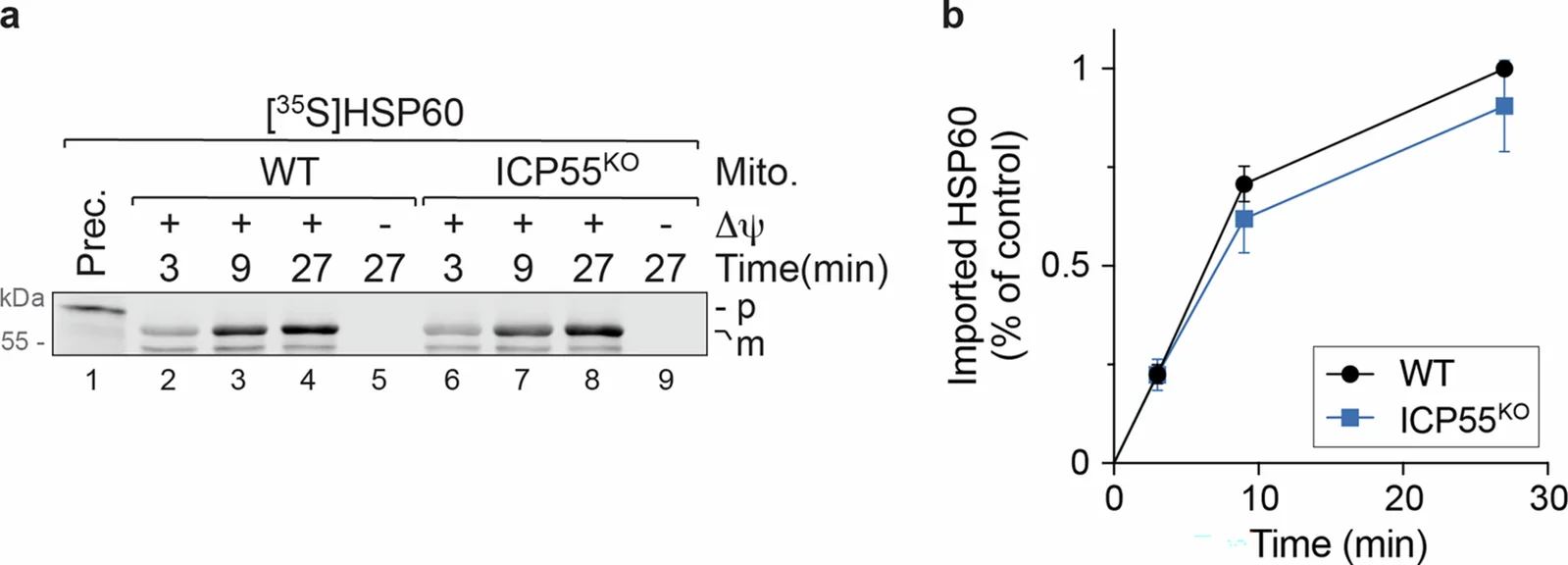
Additional N-terminal residue does not impact on HSP60 import. **a**) Import of radiolabelled HSP60 precursor into mitochondria isolated from wild-type (WT) or ICP55^KO^ mitochondria. Where indicated the membrane potential (Δψ) was dissipated prior to the import reaction. Samples were treated with proteinase K and analyzed by SDS–PAGE and autoradiography. **b**) Quantification of import kinetic from (**a**). Data represent mean ± SEM from three replicates. Source data

This shift toward lower molecular weight species could result either from impaired oligomerization during assembly or from reduced stability of the assembled complex. To distinguish between these possibilities, we first monitored HSP60 assembly dynamics by importing radiolabeled HSP60 into isolated WT and ICP55^KO^ mitochondria, followed by SDS– and BN–PAGE to assess import and complex formation. Although mitochondrial import was unaffected, a mild reduction in the assembly rate of newly imported HSP60 into the mature barrel was observed in ICP55-deficient mitochondria (Fig. 3d,e and Extended Data Fig. 5a,b).

Next we tested the structural stability of the assembled HSP60 complex by challenging protein-protein interactions with increasing concentrations of urea. In WT mitochondria, the HSP60 complex was stable and resisted dissociation at low urea concentrations (Fig. 3f,g). In contrast, in ICP55^KO^ mitochondria, the complex rapidly disassembled even under mild denaturing conditions. Importantly, re-expression of ICP55 restored HSP60 complex stability.

These findings reveal that the incorporation of unprocessed HSP60 impairs the stability of the chaperonin complex. Although assembly efficiency is only modestly affected, the resulting monoheptamer is structurally destabilized and prone to disintegration. Thus, loss of ICP55 not only perturbs mitochondrial energy metabolism via defective respiratory chain assembly, but might also compromise mitochondrial proteostasis by destabilizing HSP60, the major folding chaperone of the matrix.

### Complexome profiling reveals destabilization of respiratory chain complexes and HSP60 assemblies in ICP55^KO^ mitochondria

**Fig. 4:**
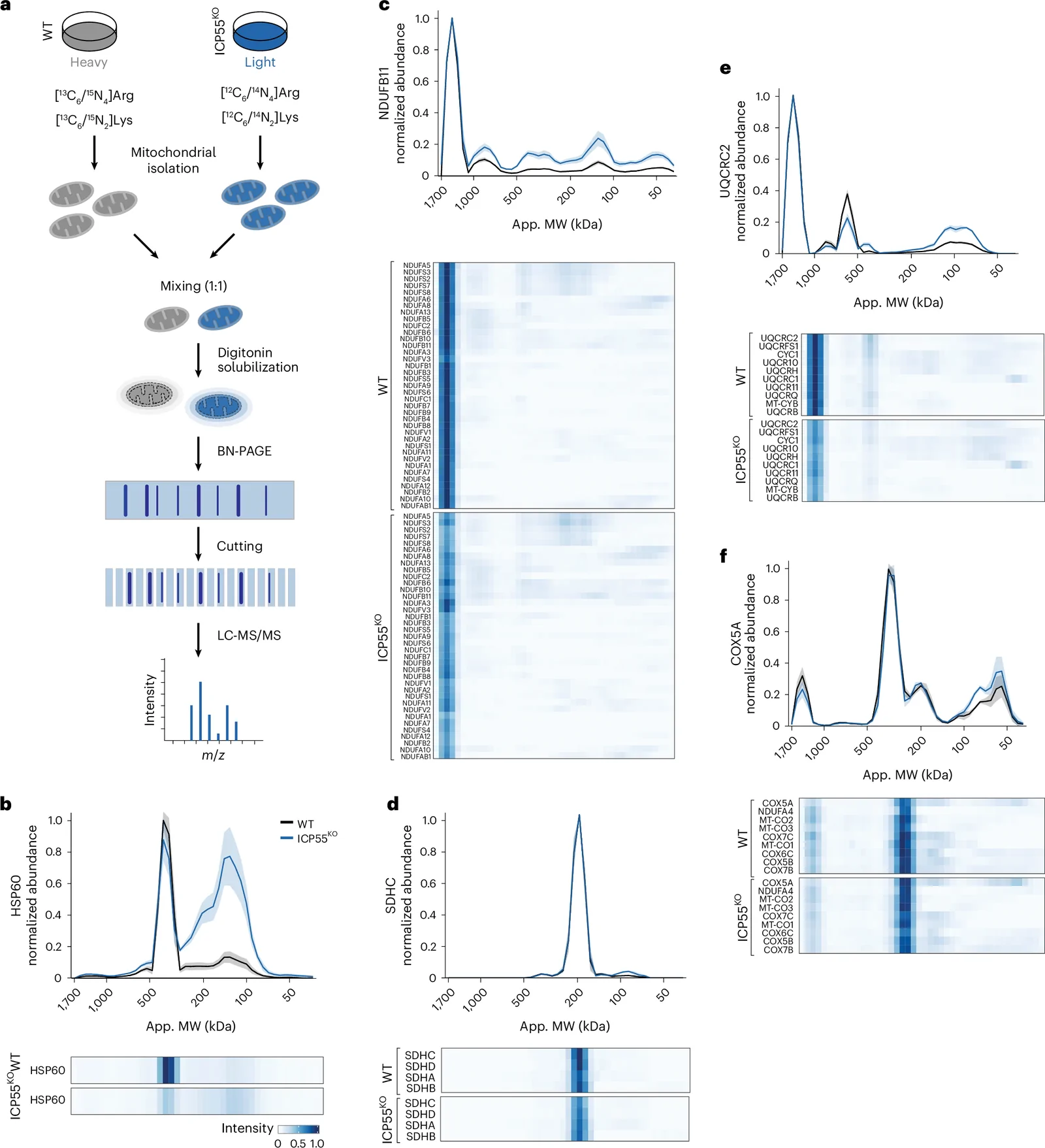
SILAC-based complexome profiling. **a**, Schematic representation of SILAC/complexome profiling (Supplementary Table 5). **b**, Complexome profiles of HSP60 in WT and ICP55^KO^ mitochondria. Solid bold lines represent the mean distribution profiles, and shaded bands represent s.e.m. (*n* = 4 independent biological replicates per genotype, dis-elution score nominal *P* ≤ 0.05, two-sided Student’s *t*-test). Profiles were normalized within replicates. The heatmap shows the mean HSP60 abundance in each fraction across the four biological replicates, normalized to the maximum intensity detected for that protein. App. MW, apparent molecular weight. **c**–**f**, Complexome profiles of the ICP55 substrates NDUFB11 (subunit of CI; **c**), SDHC (subunit of CII; **d**), UQCRC2 (subunit of CIII; **e**) and COX5A (subunit of CIV; **f**). Bold lines represent the mean distribution profiles, and shaded bands represents s.e.m. (*n* = 4 independent biological replicates per genotype). Profiles were normalized within replicates. The heatmaps show all identified subunits of the ETC CI–CIV^26^. Each heatmap cell represents the mean abundance for the indicated protein, genotype and fraction across the four biological replicates. Abundance in each row was normalized to the maximum intensity detected for that protein. Source data

**Extended Data Fig. 6:**
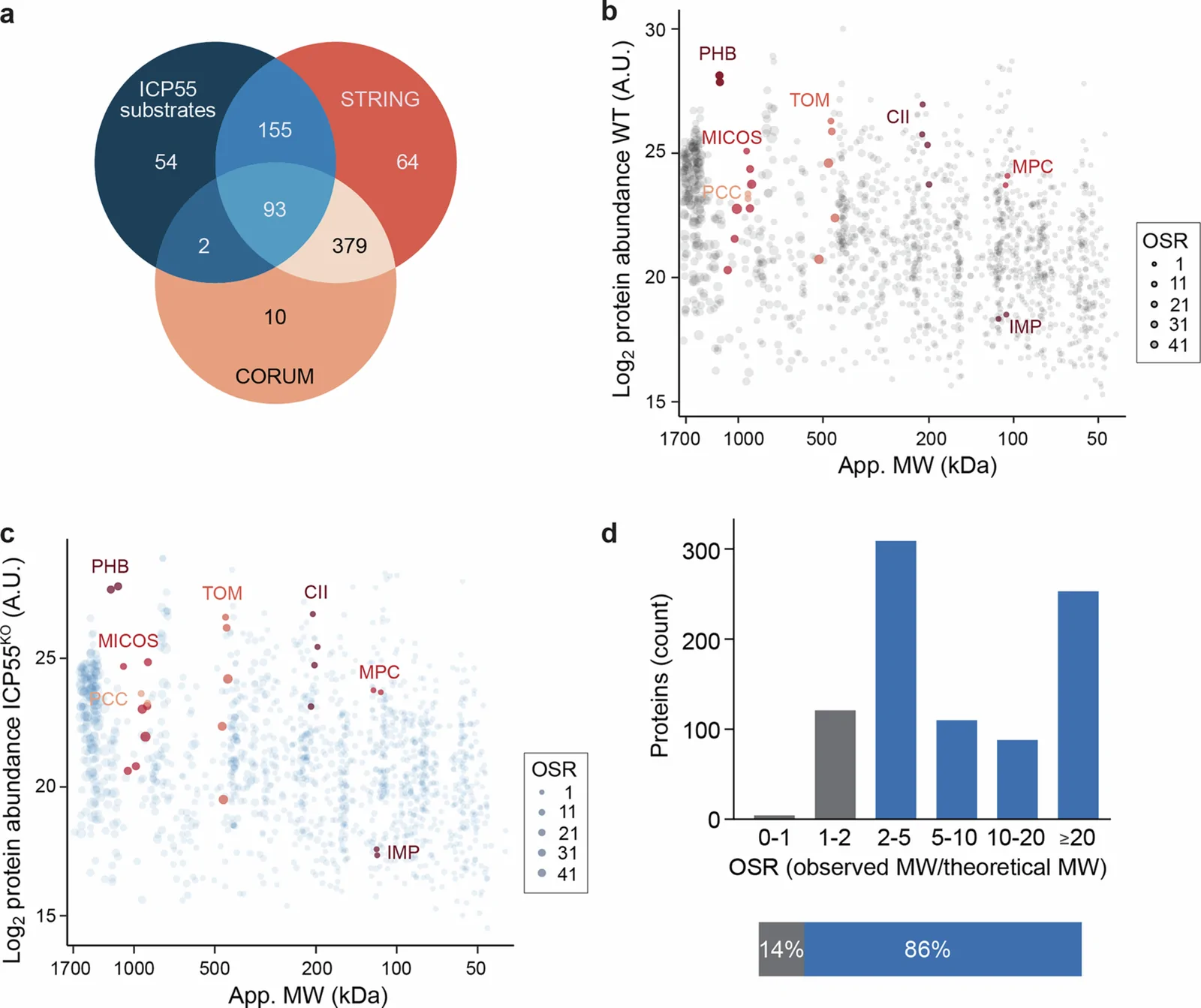
Validation of global complexome profiling. **a**) Venn diagram depicting the intersection between ICP55 substrates (experimentally identified and predicted) and proteins annotated as members of complexes in the CORUM and STRING (medium score) databases. **b**) Scatter plot displaying all deconvoluted protein peaks detected in migration profiles of 933 mitochondrial proteins from wild-type (WT) mitochondria (Supplementary Table 5). Peaks were derived from the summed migration profiles (n = 4 independently isolated mitochondrial preparations). The y-axis represents the log_2_ maximum peak intensity obtained after deconvolution, and the x-axis indicates the corresponding apparent molecular weight. The subunits of the following known mitochondrial protein machineries are highlighted: Prohibitin (PHB), mitochondrial contact site and cristae organizing system (MICOS), propionyl-CoA carboxylase (PCC), translocase of the outer mitochondrial membrane (TOM), electron transport chain complex II (CII), mitochondrial pyruvate carrier (MPC), mitochondrial inner membrane protease (IMP). **c**) All deconvoluted protein peaks detected in migration profiles of 933 mitochondrial proteins from ICP55^KO^ mitochondria (Supplementary Table 5). Peaks were derived from the summed migration profiles (n = 4 independently isolated mitochondrial preparations). The y-axis represents the log_2_ maximum peak intensity obtained after deconvolution, and the x-axis indicates the corresponding apparent molecular weight. The subunits of known mitochondrial protein machineries are highlighted (for details see (**b**)). **d**) Histogram showing the number of proteins grouped by oligomeric state ratio (OSR; defined as observed molecular weight divided by theoretical molecular weight). The bottom bar depicts the relative proportion of proteins with OSR ≥ 2, corresponding to proteins incorporated into complexes, compared to proteins with OSR < 2, corresponding to monomeric proteins. Source data

The analysis of the ETC and HSP60 complexes demonstrated that incorporation of proteins bearing a single additional N-terminal amino acid impairs complex integrity in ICP55^KO^ mitochondria (Figs. 2 and 3). Given that the majority of ICP55 substrates are components of multimeric protein assemblies (Extended Data Fig. 6a)^34,35^, we sought to assess complex stability in an unbiased and quantitative manner across the mitochondrial proteome. To this end, we employed stable isotope labeling by amino acids in cell culture (SILAC) in combination with complexome profiling (Fig. 4a)^36^. In brief, mitochondria isolated from SILAC-labeled WT and ICP55^KO^ cells were mixed in a 1:1 ratio and solubilized using the mild non-ionic detergent digitonin, which preserves native protein-protein interactions. The resulting lysates were separated by BN–PAGE, and the gel lane was sliced into 44 equally spaced fractions, each of which was subjected to quantitative LC-MS/MS analysis. As a result, we obtained distribution profiles of 2,989 proteins, of which 933 were annotated as mitochondrial^21^ (Supplementary Table 5). The migration range of the BN–PAGE extended from ~50 to 1,700 kDa, and resolution across the gradient was uniform between WT and ICP55^KO^ samples (Extended Data Fig. 6b,c). To determine the extent to which mitochondrial proteins participate in higher-order assemblies, we assessed the apparent molecular weight of all detected mitochondrial proteins relative to their theoretical monomeric mass. Remarkably, 86% exhibited migration at higher molecular weights, indicating that the vast majority of mitochondrial proteins exist as constituents of protein complexes under these native conditions (Extended Data Fig. 6d).

To validate the integrity and feasibility of our complexome approach, we first examined the distribution profiles of HSP60 and the ETC complexes. In both cases, the distribution profiles mirrored our previous biochemical observations, revealing decreased abundance of fully assembled HSP60 and ETC complexes in ICP55^KO^ mitochondria (Fig. 4b–f, heatmaps). For relative comparison of protein distributions across protein complexes, we normalized the intensity of the main assembly peak in each condition, WT and ICP55^KO^, to 1 (Fig. 4b–f, distribution profiles). In WT mitochondria, HSP60 migrated predominantly in the high molecular weight region, forming a sharp peak centered around ~450 kDa. This observation is consistent with the native heptameric barrel structure and corroborates our previous immunoblot data (Figs. 3b and 4b). By contrast, in ICP55^KO^ mitochondria, the HSP60 migration profile exhibited a bimodal distribution, with one peak co-migrating with the WT (~450 kDa) and a second peak of similar intensity emerging at ~120 kDa, consistent with the presence of unassembled or dissociated HSP60. The aberrated distribution profiles are therefore indicative of reduced complex stability in the absence of ICP55.

A similar pattern emerged for respiratory chain subunits known to be ICP55 substrates. In ICP55^KO^ mitochondria, we observed a marked increase in subcomplexes and lower molecular weight forms (distribution profiles of NDUFB11 for CI, SDHC for CII, UQCRC2 for CIII and COX5A for CIV; Fig. 4c–f), suggesting either an accumulation of assembly intermediates or enhanced dissociation of mature complexes. These changes occurred in parallel with a decrease in mature supercomplex abundance, pointing to compromised supercomplex stability as a direct consequence of impaired N-terminal processing (Fig. 4c–f, heatmaps).

### Loss of ICP55 globally disrupts mitochondrial complex integrity

Our complexome profiling revealed that the vast majority of mitochondrial proteins in human cells assemble into higher molecular weight complexes and that processing defects arising from ICP55 loss compromise the stability of key mitochondrial complexes, including HSP60 and the respiratory chain. Given that ~300 proteins are estimated to represent ICP55 substrates (based on experimental and predicted data), we next asked whether the absence of ICP55 exerts a global impact on mitochondrial complex stability.

**Extended Data Fig. 7:**
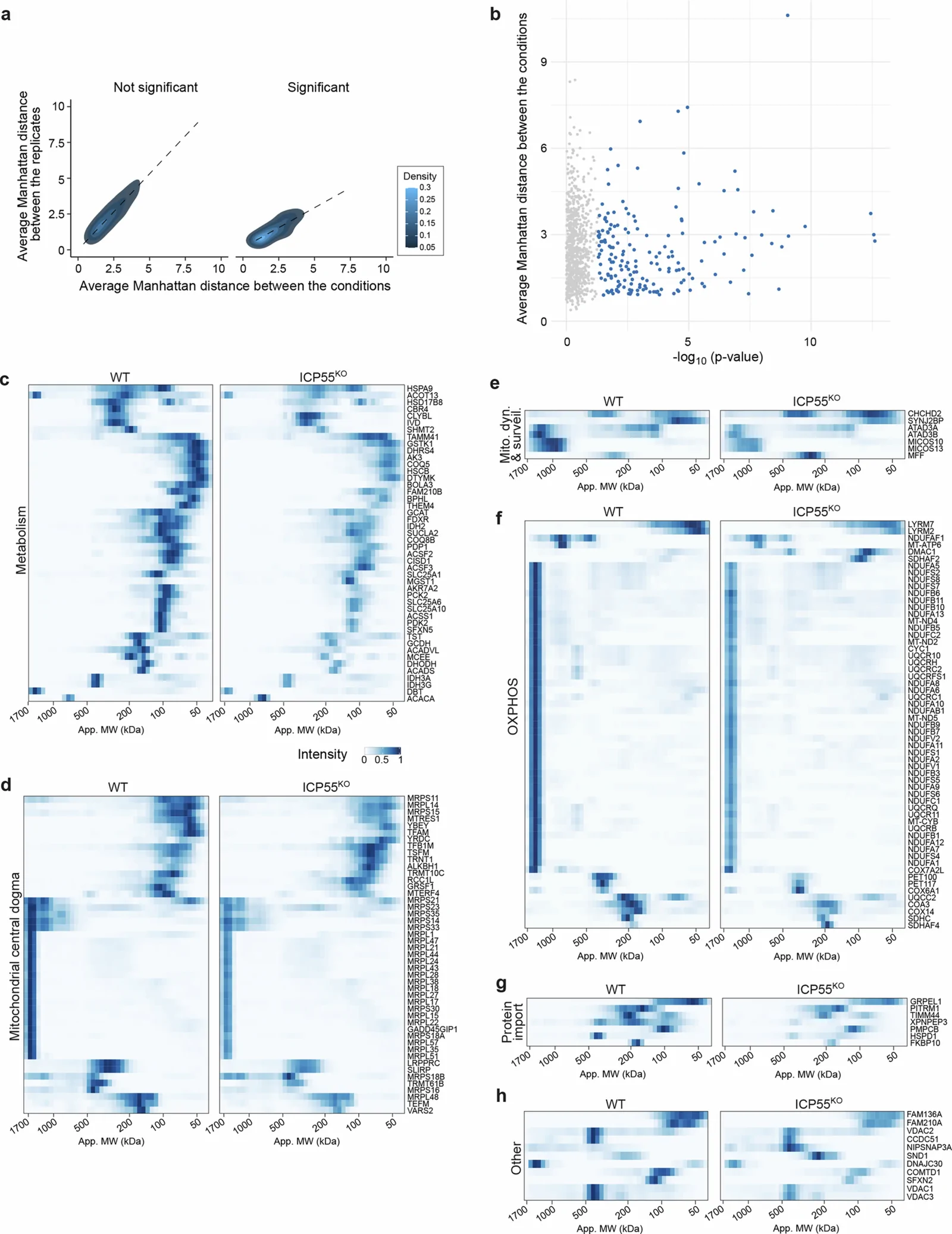
Manhattan distance analysis of global complexome. **a**) Distribution of average Manhattan distances between replicates compared to the average Manhattan distances between conditions for proteins with significantly altered distribution profiles and those without detectable changes. **b**) Distribution of 933 mitochondrial proteins quantified in the complexome profiling. Blue dots, proteins with significantly altered complexome profiles between wild-type and ICP55^KO^ mitochondria based on the dis-elution score (n = 4 independent biological replicates per genotype, dis-elution score nominal p-value ≤ 0.05 two-sided Student’s t-test). **c**) – **h**) Heatmaps of 177 proteins with altered complex behavior based on the dis-elution score (**c**, metabolism; **d**, mitochondrial central dogma; **e**, mitochondrial dynamics and surveillance (Mito. dyn. & surveil.); **f**, oxidative phosphorylation (OXPHOS); **g**, protein import; **h**, other). Heatmaps depict distribution profiles of proteins whose patterns significantly differed between the tested conditions. Each heatmap cell represents the mean abundance for the indicated protein, genotype, and fraction across the four biological replicates. Abundance in each row was normalized to the maximum intensity detected for that protein (n = 4 independent biological replicates per genotype, dis-elution score nominal p-value ≤ 0.05 two-sided Student’s t-test). App. MW, apparent molecular weight. Source data

**Extended Data Fig. 8:**
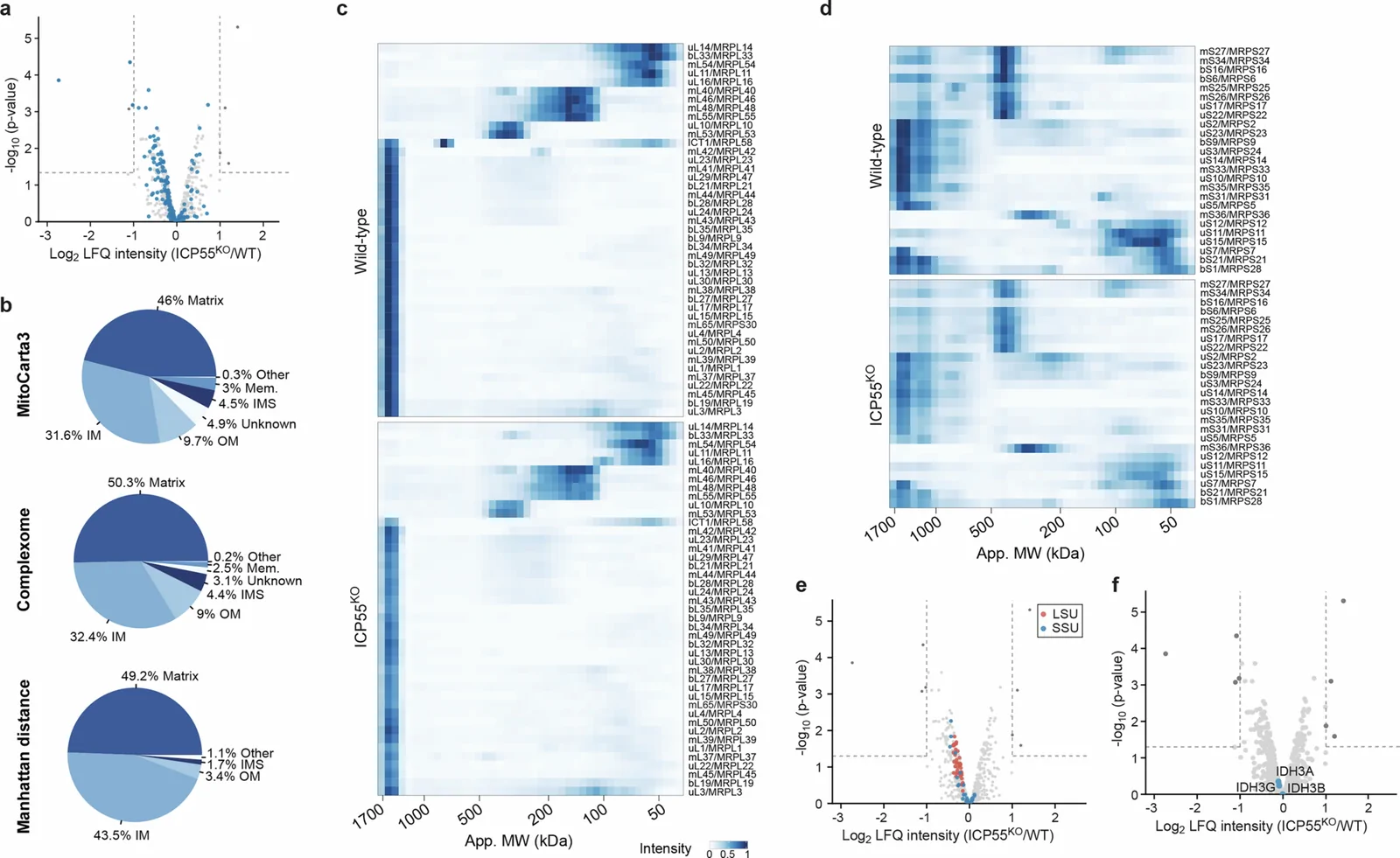
Global effects on mitochondrial complex integrity upon loss of ICP55. **a**) Volcano plot of mitochondrial proteins quantified by proteomic analysis of wild-type (WT) and ICP55^KO^ cells (Supplementary Table 4). n = 5 replicates. Horizontal line, LIMMA-moderated two-sided Student´s t-test FDR-adjusted p-value = 0.05; left and right vertical lines, twofold decrease or increase in abundance, respectively. Blue dots, 177 proteins with changed complexome distribution profiles according to the dis-elution score. **b**) Distribution of mitochondrial proteins into different mitochondrial sublocalisation. MitoCarta3.0 serves as a reference set for the mitochondrial proteome. OM, outer mitochondrial membrane; IMS, intermembrane space; IM, inner mitochondrial membrane; Mem. Membrane. **c**) Heatmap of proteins forming the large subunit of the mitochondrial ribosome^3^. Each heatmap cell represents the mean abundance for the indicated protein, genotype, and fraction across the four biological replicates. Abundance in each row was normalized to the maximum intensity detected for that protein. App. MW, apparent molecular weight. **d**) Heatmap of proteins that are subunits of the small subunit of the mitochondrial ribosome^38^. Each heatmap cell represents the mean abundance for the indicated protein, genotype, and fraction across the four biological replicates. Abundance in each row was normalized to the maximum intensity detected for that protein. **e**) Volcano plot of mitochondrial proteins quantified by proteomic analysis of WT and ICP55^KO^ cells (Supplementary Table 4). n = 5 replicates. Red dots, proteins of the large mitochondrial ribosome subunit; blue dots, proteins of the small mitochondrial ribosome subunit. **f**) Volcano plot of mitochondrial proteins quantified by proteomic analysis of WT and ICP55^KO^ cells (Supplementary Table 4). n = 5 replicates. Source data

To investigate this, we employed a statistical approach that compares protein migration profiles between WT and ICP55^KO^ mitochondria using the Manhattan distance metric, referred to as the dis-elution score (Extended Data Fig. 7a,b). This approach identified 177 proteins with significant alterations (nominal *P* ≤ 0.05; see Methods for details), reflecting either protein redistribution across protein assemblies or changes in protein assembly abundance (Extended Data Fig. 7c–h). Notably, the observed decreases in assembly abundance could not be explained by a globally reduced steady-state level of the corresponding proteins (Extended Data Fig. 8a).

Given their mitochondrial biogenesis pathways, ICP55 substrates predominantly localize to the matrix and IM, and, consistent with this, all experimentally identified ICP55 substrates are annotated to these two compartments (Supplementary Table 3). Accordingly, the 177 proteins exhibiting altered migration profiles were mainly derived from these two subcompartments, while proteins from the intermembrane space (IMS) and outer membrane (OM) were underrepresented (Extended Data Fig. 8b). The matrix and IM are more protein-dense than the IMS and OM and are therefore expected to harbor the majority of protein complexes and protein machineries^37^.

**Fig. 5:**
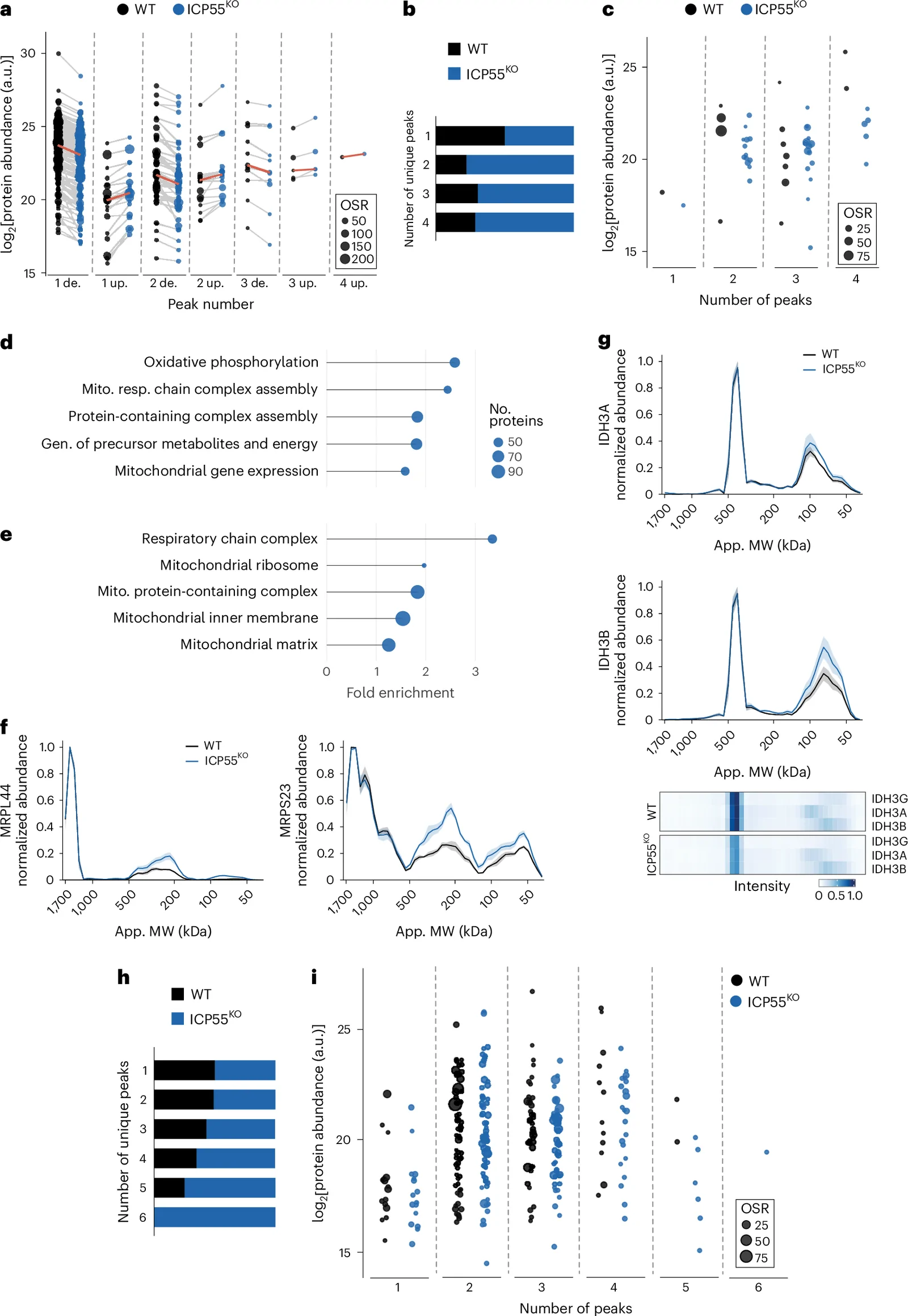
Defective N-terminal processing globally disrupts mitochondrial complex architecture. **a**, Trajectory plot linking corresponding peaks of altered proteins in WT and ICP55^KO^ mitochondria (Extended Data Fig. 7a,b). Protein migration profiles were obtained from four independent biological replicates per genotype (*n* = 4 independently isolated mitochondrial preparations for WT and *n* = 4 for ICP55^KO^). Summed profiles were generated from reproducible replicates (Pearson correlation coefficient ≥ 0.7); individual profiles that did not meet this criterion were excluded. Details of protein migration-profile deconvolution are provided in the Methods. Peaks identified in complexome profiles were numbered for each protein individually, with peak 1 corresponding to the highest apparent molecular weight (largest assembly) and increasing peak numbers reflecting progressively lower molecular weight species. Corresponding protein peaks were grouped according to their abundance trends between ICP55^KO^ and WT, separating those downregulated in condition ICP55^KO^ (*x* axis, de.) from those upregulated (up.). Red lines indicate the median log_2_ abundance change among the protein peaks in each group. The size of dots represents OSR. The *y* axis displays relative protein abundance [log_2_] of the observed unique protein peaks. **b**, Representation of the ratio of 45 altered proteins with 50 unique peaks in WT or ICP55^KO^. Peaks were derived from the summed profiles (*n* = 4 independently isolated mitochondrial preparations for WT and *n* = 4 for ICP55^KO^). Only reproducible profiles (Pearson correlation coefficient ≥0.7) were included. **c**, Analysis of proteins with unique peaks from **b**. The *y* axis displays relative protein abundance [log_2_] of the observed unique protein peaks. **d**, Gene Ontology (GO) enrichment analysis of biological process terms among mitochondrial proteins with significantly altered complexome profiles (dis-elution score nominal *P* ≤ 0.05, two-sided Student’s *t*-test). Significantly enriched terms were defined using an FDR of <0.05. **e**, GO enrichment analysis of cellular compartment terms among mitochondrial proteins with significantly altered complexome profiles (dis-elution score nominal *P* ≤ 0.05, two-sided Student’s *t*-test). Significantly enriched terms were defined using an FDR of <0.01. **f**, Representative complexome profiles of MRPL44 for the large mitoribosomal subunit (left panel) and MRPS23 for the small mitoribosomal subunit (right panel). Solid bold lines represent mean profiles, and shaded bands represents s.e.m. (*n* = 4 independent biological replicates per genotype, dis-elution score nominal *P* ≤ 0.05, two-sided Student’s *t*-test). Profiles were normalized within replicates. App. MW, apparent molecular weight. **g**, Complexome profiles of the ICP55 substrates IDH3A and IDH3B in WT and ICP55^KO^ mitochondria and heatmap of all three subunits of the IDH complex. Solid bold lines represent the mean distribution profiles, and the shaded bands represent s.e.m. (*n* = 4 independent biological replicates per genotype). Each heatmap cell represents the mean abundance for the indicated protein, genotype and fraction across the four biological replicates. Abundance in each row was normalized to the maximum intensity detected for that protein. **h**, Ratio of 249 proteins with 299 unique peaks in WT or ICP55^KO^ mitochondria within the global mitochondrial complexome (total of 933 mitochondrial proteins). Peaks were derived from the summed profiles (*n* = 4 independently isolated mitochondrial preparations for WT and *n* = 4 for ICP55^KO^). Only reproducible profiles (Pearson correlation coefficient ≥ 0.7) were included. **i**, Analysis of proteins from **h**. The *y* axis displays the relative protein abundance [log_2_] of the observed unique protein peaks. Source data

To characterize the protein distribution profiles of the altered proteins, we generated a trajectory plot in which protein peaks were grouped by peak number and annotated with their oligomeric state ratio (OSR; defined as the ratio of detected complex mass to theoretical monomeric mass). In this analysis, the median abundance (Fig. 5a, red line) demonstrated that high molecular weight complexes were frequently less abundant in ICP55^KO^ mitochondria. Only a small subset of proteins displayed increased complex abundance in the mutant. Furthermore, analysis of unique protein peaks, those detected exclusively in WT or ICP55^KO^ mitochondria, revealed that multiple proteins exhibited additional peaks in the mutant, typically accompanied by lower OSR values (Fig. 5b,c). These results indicate that proteins previously incorporated into high molecular weight assemblies in WT mitochondria are instead present in smaller assemblies or possibly as monomers in the absence of ICP55, consistent with disassembly or incomplete complex formation.

To understand which mitochondrial functions might be perturbed by ICP55^KO^-induced complexome changes, we performed Gene Ontology (GO) enrichment analysis on the 177 altered proteins. GO terms for biological processes were dominated by pathways associated with oxidative phosphorylation, respiratory chain biogenesis and assembly, and mitochondrial gene expression and translation (Fig. 5d). In parallel, GO terms for cellular components highlighted enrichments for the mitochondrial respirasome, IM protein complexes, mitochondrial matrix and mitoribosome (Fig. 5e).

Following up on these findings, we examined the mitochondrial ribosome in more detail and observed a decrease in the abundance of major peaks for both the large and small mitoribosomal subunits while overall subunit abundance was only slightly decreased (Extended Data Fig. 8c–e)^38^. Additionally, several subunits displayed increased abundance at lower molecular weights in ICP55^KO^ mitochondria (Fig. 5f), in agreement with the trajectory plot data and indicative of weakened ribosomal subunit assembly or stability.

GO analysis also suggested alterations in the generation of precursor metabolites. We therefore analyzed the assembly state of key mitochondrial metabolic enzymes. Similar to the patterns observed for the respiratory chain and mitoribosome, metabolic protein complexes exhibited decreased abundance at high molecular weight, with a corresponding increase in lower molecular weight forms (Extended Data Fig. 7c). For example, the isocitrate dehydrogenase complex, a heterotetramer composed of two IDH3A subunits and one each of IDH3B and IDH3G^39^, showed a marked reduction in fully assembled complex in ICP55^KO^ mitochondria (Fig. 5g, heatmaps), while their protein levels remained unaffected (Extended Data Fig. 8f). Notably, both IDH3A and IDH3B are ICP55 substrates. In ICP55^KO^ mitochondria, we observed increased signal at lower molecular weights likely corresponding to unassembled monomeric subunits (Fig. 5g, representative distribution profiles for IDH3A and IDH3B), again consistent with impaired complex integrity due to defective N-terminal processing.

To gain a global perspective on mitochondrial complex assembly, we examined the unique peaks of all identified mitochondrial proteins (Fig. 5h,i). Across the entire dataset, a greater number of proteins in ICP55^KO^ mitochondria displayed additional peaks in the lower molecular weight range (peaks 3–6). Together, these findings demonstrate that at a proteome-wide level, mitochondrial protein complexes in ICP55^KO^ cells are often less abundant in their fully assembled, high molecular weight states and more frequently detected in smaller assemblies or as monomeric units. Thus, our data underscore a widespread destabilization of mitochondrial protein complex architecture driven by the absence of a single-amino-acid processing step.

### An additional N-terminal residue is sufficient to destabilize mitochondrial protein complexes

The broad destabilization of mitochondrial protein complexes upon ICP55 loss raised the question of whether the retention of a single additional N-terminal residue alone is sufficient to directly impair complex stability.

**Fig. 6:**
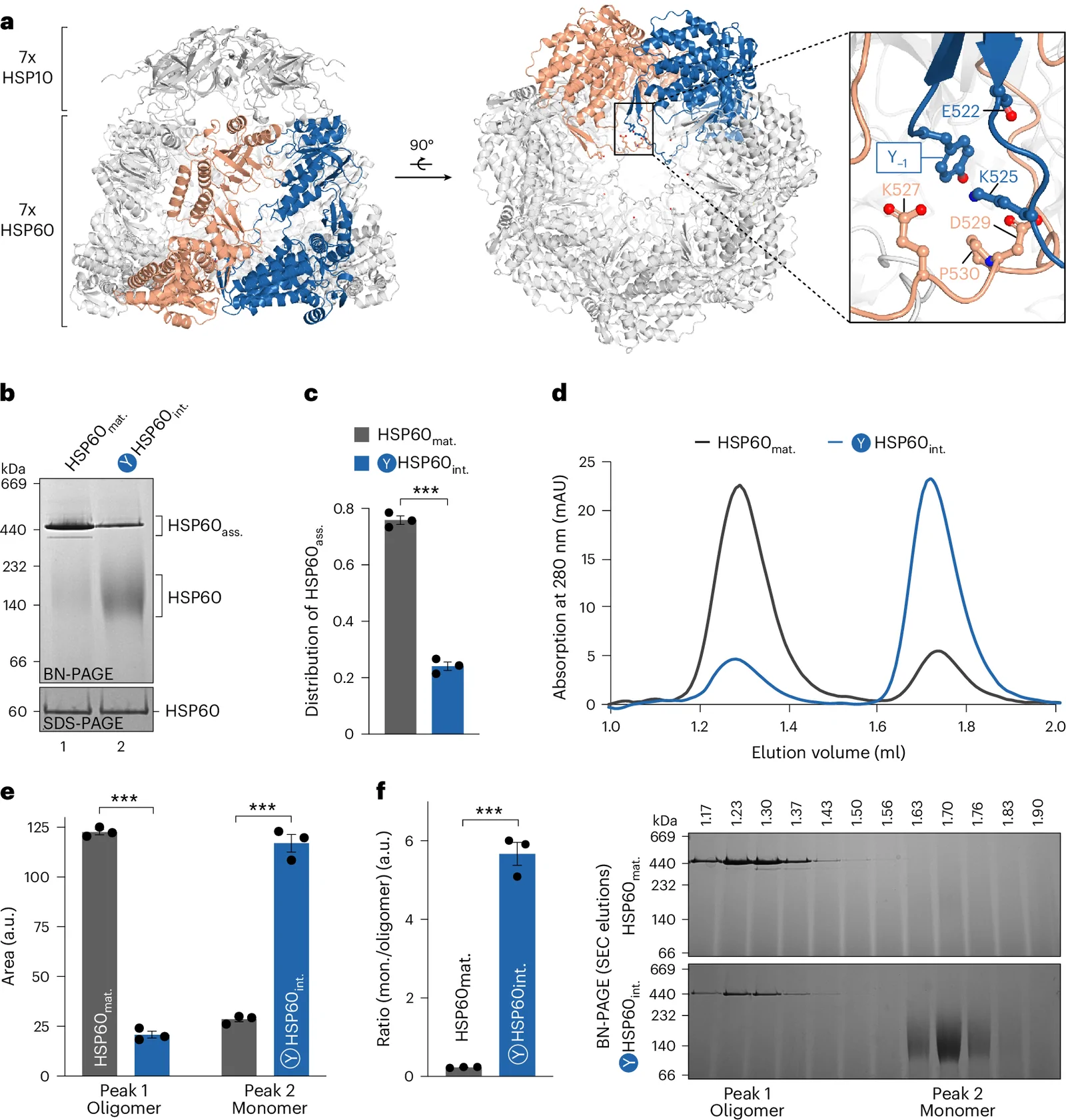
A single N-terminal residue destabilizes the HSP60 complex architecture. **a**, AlphaFold3 model of the HSP60/HSP10 barrel containing the additional tyrosine residue (Y_-1_). **b**, Analysis of the purified mature (HSP60_mat._) and intermediate HSP60 (YHSP60_int._) oligomers. Samples were analyzed on BN–PAGE (top panel) or subjected to SDS–PAGE (lower panel). Proteins were visualized by Coomassie staining. HSP60_ass._, assembled HSP60 barrel. **c**, Quantification of the assembled HSP60 from **b**. Data represent mean ± s.e.m. from three replicates. Unpaired, two-sided Student’s *t*-test was used to compare between two groups. Significances are indicated with asterisks: ***P < 0.001. Exact *P* value: *P* = 0.000075. **d**, Analysis of mature (mat.) and intermediate (int., Y) HSP60 oligomer by SEC. Purified recombinant HSP60 oligomer was applied to an S200 Increase 5/150 GL column and eluting protein species were monitored at 280 nm (chromatogram, upper panel) and analyzed by BN–PAGE and Coomassie staining (lower panel). **e**, The experiment was performed as in **d** using less protein and monitoring eluting protein species at 205 nm to quantify peak areas of the corresponding chromatograms (as depicted in **d**) representing the oligomer (peak 1, at around 1.27 ml) and the monomer (peak 2, at around 1.72 ml). Data represent mean ± s.e.m. from three replicates. Significance is indicated with asterisks: unpaired, two-sided Student’s *t*-test; ***P < 0.001. Exact *P* values: oligomer mat.:int. *P* = 7.9 × 10^−7^; monomer mat.:int., *P* = 3.4 × 10^−5^. **f**, Ratio of monomeric (mon.) and oligomeric form of mature and intermediate HSP60 from the analysis in **d**. Data represent mean ± s.e.m. from three replicates. Significance is indicated with asterisks: unpaired, two-sided Student’s *t*-test; ***P < 0.001. Exact *P* value: mat.:int. *P* = 4.9 × 10^5^. Source data

**Extended Data Fig. 9:**
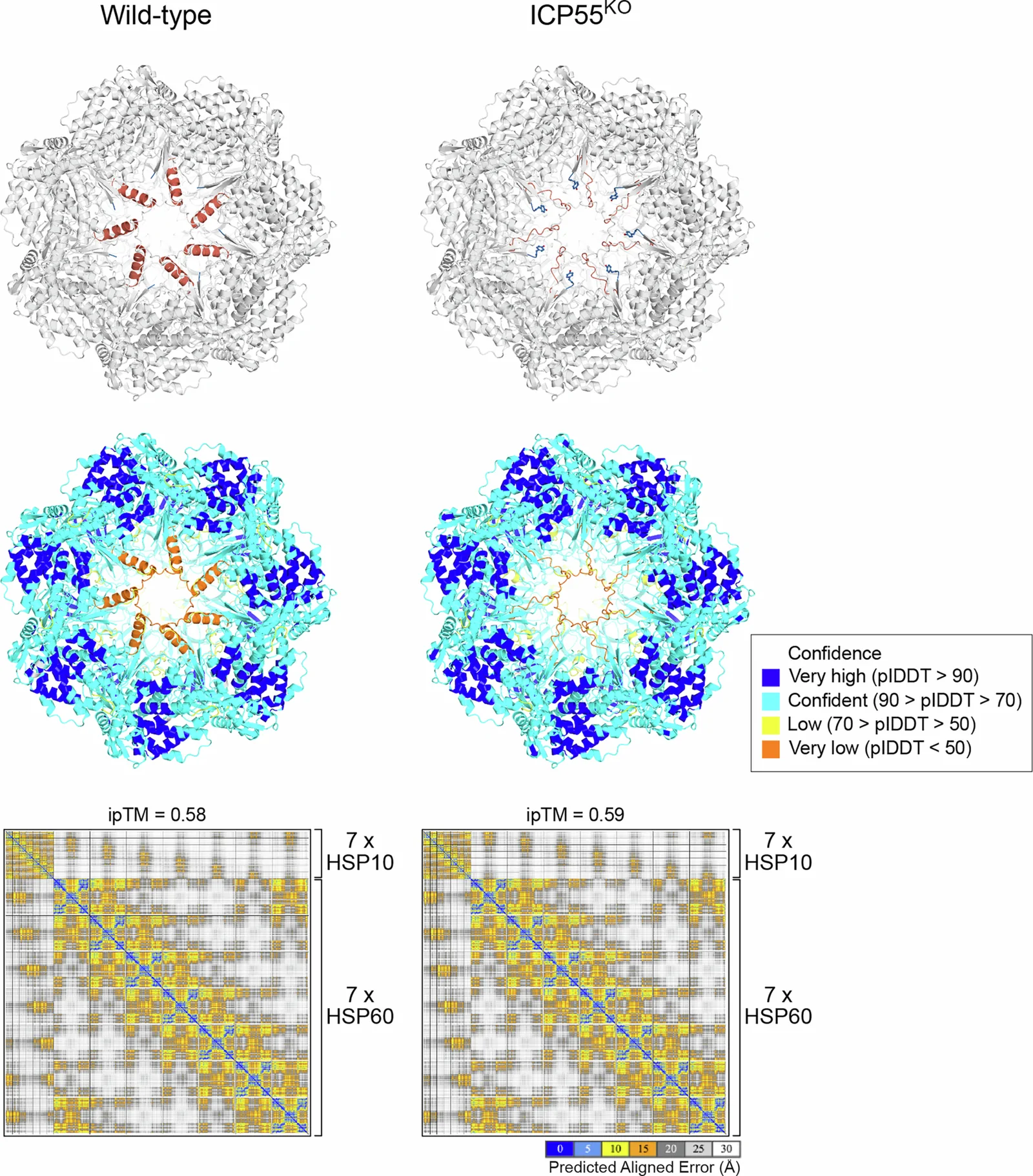
AlphaFold3 prediction of mature and intermediate HSP60 complexes. Structural prediction of the mature HSP60/HSP10 barrel (wild-type, left panels) or the barrel containing the additional tyrosine residue (ICP55^KO^, right panels) using AlphaFold3. Top, cartoon representation with the HSP60 C-termini colored in dark salmon and the N-terminal Y_-1_ depicted in blue ball-and-stick representation. Middle, cartoon representation color-coded by prediction confidence as indicated. Bottom, predicted aligned error (PAE) plots.

To investigate the molecular basis of this instability, we used AlphaFold3 to generate models of both the mature and ICP55-intermediate forms of HSP60 (Fig. 6a; refs. ^40,41^). The additional tyrosine residue at the N terminus of HSP60 faces toward the inner of the HSP60/HSP10 complex and contacts the C termini of both the same subunit and the neighboring HSP60 subunit. Although the positions of the HSP60 C termini are not predicted with high confidence (Extended Data Fig. 9), the tyrosine likely resides in a charged environment, which may lead to destabilization of the intra- and inter-subunit interactions required for proper complex formation and thereby cause the observed increase in monomeric HSP60 (Fig. 3b).

**Extended Data Fig. 10:**
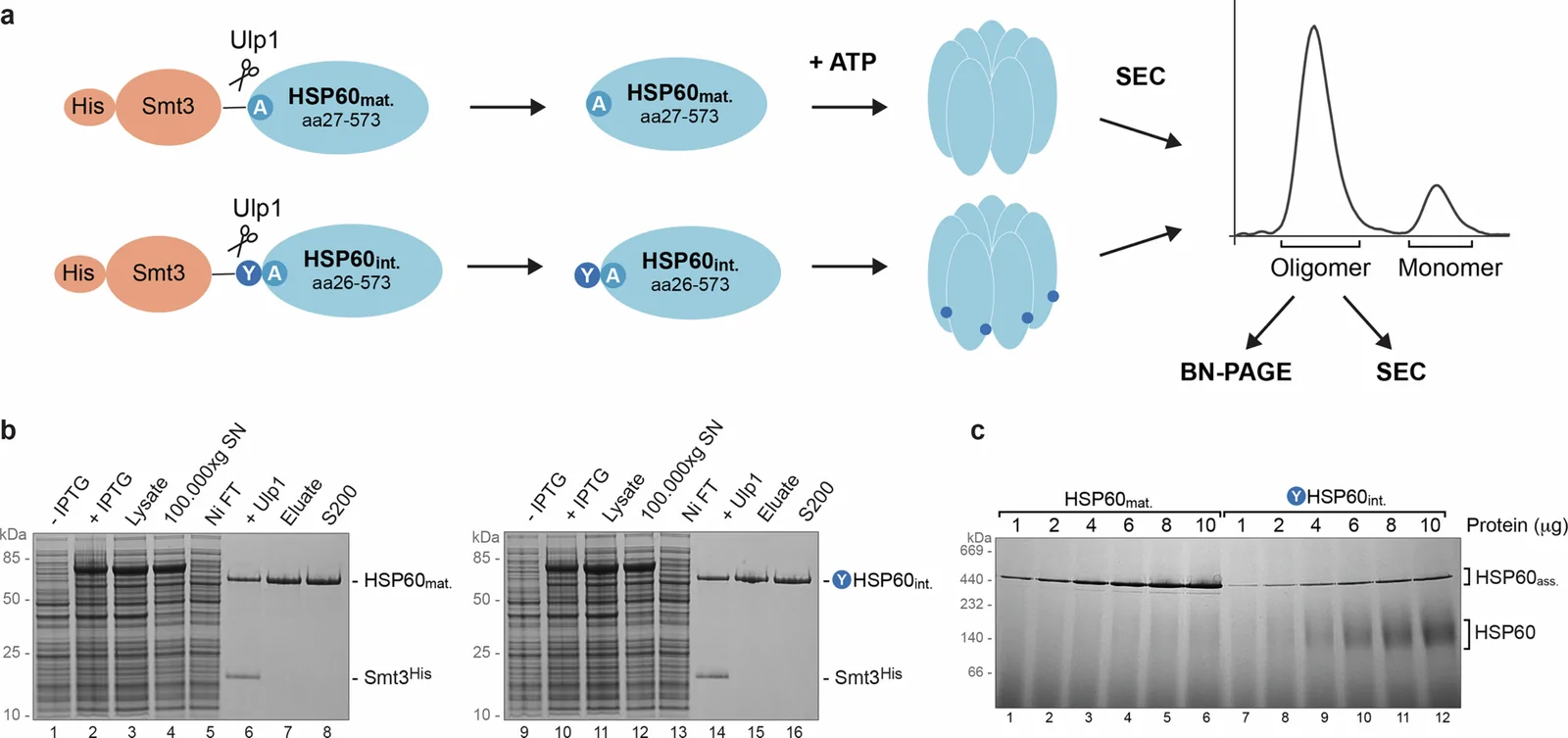
Purification of mature and intermediate HSP60 for *in vitro* analysis. **a**) Schematic representation of the purification and *in vitro* assembly strategy. **b**) Expression and purification of HSP60 (mature and intermediate form) from *E. coli*. Expression was induced by addition of IPTG. The bacterial lysate was cleared by centrifugation at 100,000 x g, and the supernatant was applied to a Ni-NTA column. Bound HisSmt3-HSP60 was cleaved by incubation with Ulp1-GST and the released protein was cleared over a GSH agarose column. The flowthrough containing HSP60 was incubated with ATP and separated by size exclusion chromatography (SEC; S200 column). Samples representing the different steps of the purification procedure were analyzed by SDS–PAGE and Coomassie staining. The purification was performed once, and the gels shown correspond to that experiment. **c**) Oligomeric HSP60 (mature and intermediate form) was analyzed on BN–PAGE and protein was visualized by Coomassie staining. HSP60_ass._, assembled HSP60 oligomer.

To experimentally test whether a single additional N-terminal residue is sufficient to destabilize this mitochondrial protein complex, we established an in vitro assembly system using recombinant HSP60. We generated variants that differ exclusively in their N-terminal residue, producing proteins that begin either with alanine (mature form) or tyrosine (ICP55 intermediate; Extended Data Fig. 10a,b). Following ATP-dependent assembly^42^, assembled and non-assembled species were separated by size exclusion chromatography (SEC), and the oligomeric HSP60 fraction was selectively collected for further analysis. We then re-analyzed these isolated oligomeric fractions by BN–PAGE and a second round of SEC to assess the stability of the pre-assembled complexes. Strikingly, whereas the mature HSP60 remained as stable oligomeric complexes, the variant carrying the additional N-terminal tyrosine was intrinsically unstable. Although the intermediate HSP60 had initially been recovered in the oligomeric fraction, re-analysis revealed rapid disassembly of this pre-assembled complex, as evidenced by a pronounced loss of oligomeric and the concomitant accumulation of monomeric species (Fig. 6b–e and Extended Data Fig. 10c). Analysis of the eluted fractions from the second size exclusion run by BN–PAGE confirmed the presence of intact oligomeric HSP60 for the mature form, but not for the intermediate form, demonstrating that the observed disassembly does not arise during BN–PAGE but reflects the intrinsic instability of the intermediate complex. Together, these results provide direct mechanistic evidence that the retention of a single additional N-terminal residue is sufficient to destabilize a mitochondrial protein complex.

## Discussion

Our study establishes N-terminal processing of mitochondrial precursor proteins as a central determinant of mitochondrial complex integrity. By providing a systematic, high-confidence definition of the human mitochondrial N-proteome, we identify the authentic ICP55 substrate repertoire and uncover R and Q as additional substrate-defining residues, a finding not predicted by existing in silico tools, extending the cleavage motifs beyond the canonical bulky hydrophobic residues identified in yeast and plants. Our data indicate that ICP55 substrates are more prevalent than previously anticipated and that human ICP55 recognizes a broader range of sequence motifs, suggesting an evolutionary divergence in substrate specificity that may reflect adaptations to the human mitochondrial proteome. Importantly, our comparison with targeting predictions shows that nearly one-third of experimentally validated substrates would have been missed by computational approaches alone, highlighting the necessity of direct experimental approaches.

**Fig. 7:**
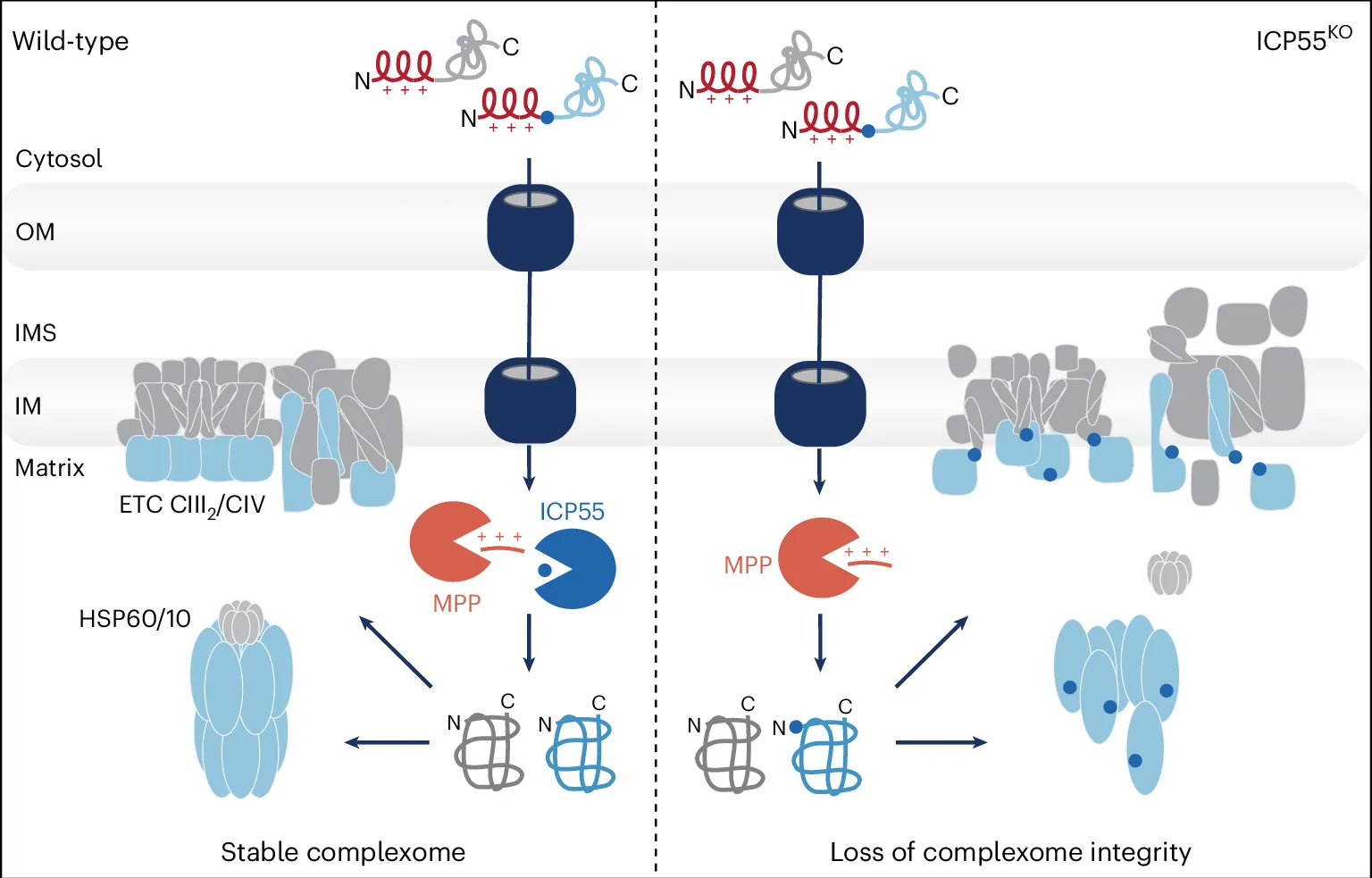
ICP55-mediated N-terminal processing safeguards mitochondrial protein complex integrity. Following mitochondrial import, precursor proteins are processed by MPP, with ICP55 subsequently removing an additional N-terminal amino acid from its substrates before their assembly into multimeric protein complexes. Loss of ICP55 causes retention of this single additional N-terminal residue, destabilizing protein-protein interactions and shifting mitochondrial protein assemblies from mature complexes toward smaller subcomplexes, thereby compromising mitochondrial proteostasis.

Mechanistically, our findings reveal that defective ICP55-mediated processing has consequences for mitochondrial protein complex integrity across a broad set of assemblies (Fig. 7). The decreased abundance and increased urea sensitivity of ETC complexes and HSP60 assemblies indicate that the retention of a single N-terminal residue can weaken protein-protein interfaces, resulting in both impaired assembly of nascent complexes and accelerated disassembly of mature structures. The sensitivity of protein complexes to ICP55 processing defects might be due to the tight packing of their subunit interfaces or the structural constraints near their N termini, where even minimal steric alterations can compromise inter-subunit interactions. In this context, the marked increase of the respiratory chain assembly factor COX7A2L in ICP55-deficient mitochondria is notable. COX7A2L overexpression in myotubes has been shown to promote supercomplex formation and increase respiratory capacity^43^. The upregulation of COX7A2L in ICP55^KO^ cells is therefore probably a compensatory response aimed at restoring supercomplex assembly in the face of reduced complex stability. However, despite this strong induction, neither supercomplex abundance nor respiration are rescued in the knockout, indicating that impaired N-terminal processing exerts a dominant destabilizing effect that cannot be overcome by elevated COX7A2L levels.

Similarly, the destabilization of HSP60 in ICP55-deficient mitochondria is of particular interest in light of human genetic data. A homozygous D3G mutation at the mature N terminus of HSP60 causes MitCHAP-60 disease, a severe neurodegenerative disorder^44^. Analysis of the D3G mutant demonstrated profound destabilization of the heptameric barrel in vitro, leading to oligomer disassembly and impaired folding activity^45^. In direct agreement with these findings, in vitro assembly assays using recombinant HSP60 variants that differ exclusively in their N-terminal residue revealed that the variant carrying an additional N-terminal tyrosine is markedly destabilized and rapidly disassembles, whereas the mature form remains stably assembled. These data provide direct mechanistic evidence that even a single N-terminal residue is sufficient to destabilize the HSP60 complex. Importantly, these findings explain the reduced stability of HSP60 observed in ICP55-deficient mitochondria and establish a direct mechanistic parallel to the patient mutation, demonstrating that even subtle N-terminal alterations can undermine HSP60 stability and chaperone function.

Previous work in yeast demonstrated that Icp55 substrates carrying the N-terminal residues Y, L or F exhibited reduced half-lives and increased turnover, consistent with principles of the bacterial N-end rule^3^. This suggested that Icp55-mediated processing can influence protein degradation by removing destabilizing N-terminal residues. In the absence of Icp55, mitochondrial proteins retaining such destabilizing N-terminal residues would be more prone to degradation. However, the existence of a defined N-end rule pathway in mitochondria and the identity of the responsible proteases remain to be established. By integrating these previous findings with our current human dataset, we propose a model in which ICP55-mediated N-terminal processing links protein maturation to both protein complex stability and protein turnover: impaired ICP55 processing leads to destabilization and increased disassembly of mitochondrial protein complexes, as observed in our study. The resulting unassembled subunits, which retain an additional N-terminal residue, may be preferentially recognized and degraded, potentially via an N-end-rule-like mechanism.

Consistent with this model, the mechanistic parallels of incomplete N-terminal processing with HSP60 disease provide a conceptual basis for understanding the clinical manifestations of ICP55 deficiency. Patients with mutations in ICP55 present with neurological, cardiac and renal symptoms^17,46–48^, which we propose may be linked to impaired stability of essential mitochondrial complexes and a consequent disruption of mitochondrial proteostasis. Given the presence of ICP55 substrates in several complexes involved in energy conversion, various metabolic pathways and mitochondrial gene expression, even partial loss of ICP55 function could have cumulative effects over time. This raises the possibility that milder processing defects contribute to late-onset mitochondrial disorders or ageing-associated mitochondrial decline, a hypothesis that will require further validation in patient-derived cells or in vivo models.

It is now well established that proteins rarely function as isolated subunits. Instead, the vast majority are incorporated into large, multimeric machineries that execute core biological processes, from oxidative phosphorylation to protein-folding and gene expression^49,50^. This is also underscored for human mitochondrial proteins by our complexome profiling, which shows that the majority of mitochondrial proteins migrate at a higher molecular weight than their predicted monomeric mass, indicating their incorporation into stable assemblies. Beyond providing a snapshot of native organization, our SILAC-based quantitative complexome profiling offers an unbiased, proteome-wide assessment of complex integrity in WT and ICP55-deficient mitochondria. The analysis reveals a pronounced shift in ICP55 knockout mitochondria toward additional peaks at lower molecular weights, reflecting an overall tendency to form smaller assemblies and fewer fully assembled mature complexes. This destabilization is most prominent for proteins of the IM and matrix, the compartments that harbor the majority of mitochondrial protein complexes and where ICP55 substrates are localized, highlighting a broad structural impact of impaired N-terminal processing on the mitochondrial protein interaction network. Our data demonstrate that proper proteolytic maturation of complex subunits is an essential prerequisite for correct and stable assembly, and that the integrity of protein assemblies is critical for mitochondrial function.

Although our work establishes a direct link between ICP55-mediated processing and mitochondrial protein complex stability, it is based on a complete knockout cell line, which may not fully capture the subtler phenotypes of patient mutations or tissue-specific proteomes. Moreover, although HUNTER N-terminomics provides high coverage, very hydrophobic or low-abundance proteins may remain underrepresented, likely leading to an underestimation of the total number of ICP55 substrates. Future studies should address these limitations by analyzing patient-derived cells or in vivo models, and by applying structural approaches to directly visualize how extra N-terminal residues perturb subunit interfaces.

Taken together, our results position ICP55 as a key proteostatic safeguard in human mitochondria, ensuring the structural stability of multimeric matrix and inner membrane protein assemblies through precise N-terminal maturation. We propose that N-terminal proteoform control represents an underappreciated layer of mitochondrial proteostasis, with broad implications for organellar health and disease. Moreover, our integrated in silico and experimental approach provides a versatile blueprint for mapping substrate repertoires of other organellar processing proteases, enabling a deeper understanding of how protease specificity co-evolves with complex protein networks.

## Methods

### Cell culture

Human embryonic kidney (HEK293T, ATCC, CRL-3216) cells were cultured in DMEM (Gibco) containing 4.5 g l^−1^ (high-glucose medium) or 1 g l^−1^ (low-glucose medium) glucose supplemented with 10% (vol/vol) fetal bovine serum (Sigma) and 2 mM L-glutamine at 37 °C in a humidified incubator with 5% CO_2_. All functional analyses were performed with cells cultured in high-glucose medium.

### Generation of knockout and stable cell lines

ICP55 knockout HEK293T (ICP55^KO^) cells were generated by CRISPR-Cas9 genome editing as described previously^56^. The guide RNA sequence (5′-AGGTGCGGTGAACCTGGGGA-3′) targeting human *XPNPEP3* exon 8 was cloned into the pSpCas9(BB)-2A-GFP vector (gift from F. Zhang; Addgene plasmid 48138). HEK293T cells were transfected by Lipofectamine 2000 (Invitrogen, 11668027). Green fluorescent protein (GFP)-positive cells were single-cell-sorted into 96-well plates by fluorescence-activated cell sorting (FACS). Clones were expanded and screened for the loss of ICP55 by western blotting, and indel mutations were confirmed by sequencing. ICP55^KO^ cells were complemented by retroviral transduction. *XPNPEP3* cDNA with an additional C-terminal FLAG tag was cloned into the pBABE-puromycin vector. NIH239T cells were transfected by retroviral constructs using Lipofectamine LTX (Invitrogen, 15338-100). Viral supernatant was collected after 48 h and used to infect ICP55^KO^ cells in the presence of 8 μg ml^−1^ of polybrene. Positive clones were selected using 0.5 μg ml^−1^ puromycin.

### Cell proliferation assay

The cell proliferation assay was recorded with an Omni system (Axion Biosystems). A total of 10,000 cells for high-glucose medium and 15,000 cells for low-glucose medium were seeded on a poly-D-lysine-coated 48-well plate and incubated at 37 °C. The medium was changed every two days, and every 4 h the coverage of each well was scanned.

### Isolation of mitochondria

Mitochondria were isolated as previously described^57^. Cells were collected and resuspended in solution A (220 mM mannitol, 70 mM sucrose, 20 mM HEPES-KOH (pH 7.6), 1 mM EDTA, 0.5 mM PMSF and 2 mg ml^−1^ BSA) and homogenized using a glass potter. Samples were centrifuged at 800*g* for 5 min at 4 °C to remove cellular debris. Subsequently, the supernatant was subjected to centrifugation at 10,000*g* for 15 min at 4 °C. Mitochondrial pellet was resuspended in solution B (solution A without BSA).

### SDS–PAGE and BN–PAGE

Proteins were separated by non-continuous vertical SDS–PAGE. To achieve resolution down to a single amino-acid-residue difference, the acrylamide concentration in the separation gel was varied depending on the molecular weight of the protein being analyzed. To estimate protein molecular weights, the PageRuler Plus Prestained Protein Ladder (Thermo Fisher Scientific, 26619) and the Unstained Protein Standard, Broad Range Ladder (NEB, P7717) were used as molecular weight markers. Mitochondrial complexes were analyzed by BN–PAGE. Mitochondria were solubilized in ice-cold solubilization buffer (with or without urea) containing 1% (wt/vol) digitonin, 20 mM Tris-HCl (pH 7.4), 0.5 mM EDTA, 10% glycerol and 50 mM NaCl, and incubated on ice for 15 min. Samples were centrifuged at 20,000*g* for 5 min at 4°C and supernatant was loaded on 4–10% or 4–13% native gradient gel. Samples were further analyzed by autoradiography or immunoblotting.

### In organello import of radiolabeled precursor proteins

Radiolabeled HSP60 precursor proteins were synthesized in vitro using the rabbit reticulocyte lysate system in the presence of ^35^S-methionine. Radiolabeled HSP60 precursor and isolated mitochondria were incubated in import buffer (250 mM sucrose, 5 mM Mg(OAc)_2_, 80 mM KOAc, 10 mM sodium succinate, 20 mM HEPES-KOH (pH 7.4)) supplemented with 1 mM DTT and 5 mM ATP for the indicated time points at 37 °C. Membrane potential was disrupted by the addition of AVO (8 μM antimycin, 1 μM valinomycin, 20 μM oligomycin) prior to the import reaction. Samples were treated with 20 μg ml^−1^ Proteinase K to digest non-imported precursor protein. Samples were analyzed by SDS– or BN–PAGE followed by autoradiography.

For immunoblotting, primary antibodies against TOM40 (Proteintech, 18409-1-AP, 1:1,500), ATP5D (Novus Biologicals, NBP1-32365, 1:500), CLPP (Proteintech, 15698-1-AP, 1:4,000), TFAM (Proteintech, 22586-1-AP, 1:1,000), COQ7 (Proteintech, 15083-1-AP, 1:500), MMAB (GeneTex, GTX106767, 1:1,000), SDHAF2 (Cell Signaling Technology, 45849, clone D1S8D, 1:1,000), COX7A2L (Proteintech, 11416-1-AP, 1:500), SDHA (Cell Signaling Technology, #5839, 1:1,000), HSP60 (Proteintech, 15282-1-AP, 1:6,000), ATP5A (Abcam, ab14748, clone 15H4C4, 1:1,000), COX1(GR2034-7, 1:250), TOM70 (GR5005-3, 1:250), TOM20 (GR5002-4, 1:250), Rieske (GR1512-7, 1:250), VDAC (GR1517-7, 1:250), NDUFA9 (GR1524-7, 1:250) and ICP55 (GR5133-5, 1:250) were used.

### Mitochondrial respiration

Mitochondrial respiration was measured using the Seahorse XF Pro analyzer. A total of 20,000 cells were seeded per well on a poly-D-lysine-coated 96-well Seahorse plate the day before the measurement. Basal levels of oxygen consumption rate (OCR) and OCR in the presence of ETC inhibitors and uncouplers (2 μM oligomycin, 0.3 μM carbonyl cyanide-4-(trifluoromethoxy) phenylhydrazone, 1 μM antimycin and 1 μM rotenone) were measured. A Bradford assay was performed after the assay, and OCR results were normalized to total protein content.

### AlphaFold structure prediction

HSP60/HSP10 complex models were generated with AlphaFold3^40,41^ using the web interface with standard settings. Predicted aligned error (PAE) plots were visualized using UCSF ChimeraX^58^ and structures using PyMOL (www.pymol.org).

### Expression and purification of recombinant HSP60

Mature HSP60 (aa 27–573) and intermediate HSP60 (aa 26–573) were cloned into BamHI/XhoI-digested pHisSmt3 vector^59^ by HiFi DNA assembly with the following primers: mature HSP60 forward, TCACAGAGAACAGATTGGTGGAGCCAAAGATGTAAAATTTGGTGC; mature HSP60 reverse, AGTGGTGGTGGTGGTGGTGCTTAGAACATGCCACCTCC; intermediate HSP60 forward, TCACAGAGAACAGATTGGTGGATATGCCAAAGATGTAAAATTTGG; intermediate HSP60 reverse, AGTGGTGGTGGTGGTGGTGCTTAGAACATGCCACCTCC.

Expression and purification were performed by combining protocols for HisSmt3-tagged proteins with an HSP60-specific procedure^42,59^. The HisSmt3-tagged HSP60 variants were expressed in Rosetta (DE3) cells grown in LB medium containing 30 μg ml^−1^ kanamycin, 1 mM MgCl_2_ and 0.1% (wt/vol) glucose by addition of 0.5 mM IPTG for 4 h at 25 °C.

Cells were lysed in 50 mM Tris-HCl (pH 7.5), 200 mM NaCl, 5% glycerol and 2 mM β-mercaptoethanol supplemented with 1 mM Pefabloc, 1 μg ml^−1^ aprotinin, 1 mM CaCl_2_, 2.5 mM MgCl_2_, 10 mM MgSO_4_, 10 μg ml^−1^ DNase I and 10 mM imidazole using an EmulsiFlex C5 homogenizer (Avestin). Lysates were clarified by centrifugation at 100,000*g* for 1 h at 4 °C, and the supernatant was applied to a Ni-NTA agarose column. The column was washed with 50 mM Tris-HCl (pH 7.5), 200 mM NaCl, 2 mM β-mercaptoethanol, 1 μg ml^−1^ aprotinin and 20 mM imidazole, followed by a brief wash with the same buffer lacking imidazole. Bound protein was cleaved by incubation with 1 μM Ulp1-GST in the liquid phase overnight at 4 °C. The eluate was subsequently passed through a glutathione agarose column to remove Ulp1-GST.

### Reconstitution and purification of oligomeric HSP60

To induce oligomerization, purified untagged mature and intermediate HSP60 (11 mg at 22 mg ml^−1^ final concentration) in gel filtration buffer (50 mM Tris-HCl (pH 7.5), 200 mM NaCl, 5% glycerol, 10 mM MgCl_2_) was supplemented with 4 mM ATP, 20 mM KCl and 20 mM Mg(OAc)_2_ and incubated for 1 h at room temperature. The oligomerization reactions were subsequently subjected to SEC in an S200 Increase 10/300 GL column in gel filtration buffer, and fractions corresponding to heptameric HSP60 were collected and flash-frozen in liquid nitrogen as described previously^42^.

### Analysis of the HSP60 oligomeric state

To assess the oligomeric state of recombinant HSP60, purified heptameric HSP60 was thawed rapidly, kept at room temperature until analysis, and subjected to analysis within a few minutes. For analysis by SEC, 30 μg (for quantification, performed in triplicate) or 60 μg (for subsequent BN–PAGE) HSP60 oligomer was injected onto a S200 Increase 5/150 GL column (3-ml column volume) and eluted in 20 mM Tris-HCl (pH 7.5), 50 mM NaCl, 5% glycerol, 1 mM DTT at a flow rate of 0.4 ml min^−1^, and eluting species were monitored at 280, 260 and 205 nm. For quantification, the peak areas of the 205-nm absorption curve corresponding to the heptamer (at 1.28-ml mature, 1.27-ml intermediate HSP60) and monomer (at 1.73-ml mature, 1.72-ml intermediate HSP60) were calculated using the evaluation module of Unicorn 5.31 software (GE Healthcare, now Cytiva) based on a zero baseline. For analysis by BN–PAGE, 12 μg of HSP60 oligomer or 66 μl of S200 elution fraction was mixed with 10× BN–PAGE loading dye, separated on a 4–13% discontinuous BN–PAGE system, and the Coomassie background was destained to reveal the protein bands.

### Quantitative proteome analysis

Proteins were extracted from isolated WT and ICP55^KO^ mitochondria in denaturing extraction buffer (4% SDS, 100 mM HEPES, 5 mM EDTA, pH 7.5), heated to 90 °C for 15 min, before reduction with 30 mM DTT and alkylation with 30 mM chloroacetamide (CAA), then 100 μg of proteome was purified using SP3 beads and digested with trypsin (1:100 (w/w)). Peptides were purified with self-packed C18 Stage tips, dried in a vacuum concentrator and re-dissolved in 2% acetonitrile, 0.5% acetic acid. An estimated 1 μg of peptides was analyzed per sample using an UltiMate 3000 RSLC nano-HPLC system (Thermo) operated in two-column set-up (a 2-cm μPAC C18 trap column, 50-cm μPAC C18 microarray pillar column, Thermo) online-coupled to an Impact II Q-TOF mass spectrometer (Bruker) with CaptiveSpray source (Bruker) as described in ref. ^60^. Peptides were separated over 90 min with a gradient of 2 to 30% solvent B (100% acetonitrile with 0.1% formic acid (FA)) in solvent A (HPLC-water, 0.1% FA) at a flow rate of 600 nl min^−1^. MS data were acquired in data-independent acquisition (DIA) mode using Compass HyStar v5.1 (Bruker) with OTOF control v 5.2 (Bruker) and the Chromeleon Plugin v 7.21 (Thermo). The quadrupole was set to filter precursor ions of a set mass window of 25 *m*/*z* with an overlap of 0.5 *m*/*z* on either side of the window. Each cycle consisted of one MS1 acquisition followed by 32 MS2 windows of 25 *m*/*z* with an overlap of 1 *m*/*z* acquired at 4 Hz. Bruker files were converted to mzML format using MSConvert (v 3.0.21229-9668f52)^61^ with the options ‘Write index’, ‘TPP compatibility’ and ‘Use zlib compression’ enabled. The default ‘peakPicking’ filter was applied first, followed by the ‘zeroSamples’ filter. Raw DIA spectra were matched to peptide sequences with the UniProt canonical human reference proteome (downloaded 2024-12-12, 20644 entries) using FragPipe (v22.0)^62^ with the integrated MSFragger (v4.1) search engine and DIA-NN (v1.8.2 beta 8) for quantification^63^. Searches considered fully tryptic peptides with up to two missed cleavages, peptide length 7–45 amino acids, peptide mass range 500–5,000 Da, carbamidomethylation of cysteine residues (+57.02146 Da) as fixed modification, oxidation of methionine (+15.9949 Da) and N-terminal acetylation (+42.0106 Da) as variable modifications. Peptide and protein identifications were filtered at a 1% false discovery rate (FDR) at both the peptide-spectrum match and protein level using Philosopher (v5.1.1). Resulting protein identifications were filtered for proteins quantified in ≥3 of 5 replicates of at least one condition and restricted to proteins listed in MitoCarta3.0 to focus on known mitochondrial proteins. Differences in protein abundance with a LIMMA-moderated, FDR-adjusted *t*-test^64^ *P* < 0.05 were considered significant.

### N-terminome analysis

Proteins were extracted from isolated WT and ICP55^KO^ mitochondria in denaturing extraction buffer (4% SDS, 100 mM HEPES, 5 mM EDTA, pH 7.5). N-terminal peptides were enriched essentially as described in ref. ^65^. In brief, proteins were reduced, alkylated and labeled by reductive dimethylation with heavy formaldehyde (^12^CD_2_O for digestion with trypsin and ^13^CD_2_O for digestion with legumain). Each sample was independently digested with trypsin (1:100 (w/w)) and legumain (1:50 (w/w)) overnight to increase N-terminome coverage^66^. 10 μg of digested peptides were retained to determine label efficiency (pre-HUNTER). Free α-amines were subsequently modified with undecanal and separated from N-terminal peptides by retention on reversed-phase spin cartridges (HR-X spin cartridges, Macherey and Nagel). N-terminal peptides remained in the flowthrough, were concentrated in a vacuum concentrator, desalted using homemade C18 stage tips with three layers^67^, and reconstituted in 0.1% FA.

For trypsin-digested samples, 500-ng enriched peptides were loaded on Evotips (Evosep) and separated on an Evosep One system coupled online to an Orbitrap Exploris 480 mass spectrometer (Thermo) using a 44-min gradient (30 SPD workflow, Evosep; solvent A, 0.1% FA; solvent B, 100% acetonitrile, 0.1% FA) with a nano-LC column (EV1137, 15 cm × 150 µm performance column, Evosep) maintained at 40 °C with a column oven (PRSVO-V2, Sonation). Peptides were ionized with a stainless-steel emitter with an integrated liquid junction (EV1072, Evosep) and mounted into an EasySpray adapter (EV1072, Evosep) in a NanoSpray Flex ion source (Thermo). A spray voltage of +2.0 kV was applied, and the ion transfer tube was set to 275 °C. For legumain-digested samples, 500-ng peptides were analyzed with an UltiMate 3000 RSLCnano system (Thermo) coupled online to an Exploris 480 mass spectrometer (Thermo). Peptides were loaded onto a μPAC C18 trapping column (Thermo) with a flow rate of 10 μl min^−1^, then separated on a μPAC C18 pillar array column (50-cm bed length, Thermo) using a binary solvent system (solvent A, 0.1% formic acid; solvent B, 86% acetonitrile, 0.1% formic acid). The flow rate was 500 nl min^−1^, and peptides were eluted with a gradient of 1–4% B in 2 min, 4–25% B in 24 min, 25–44% B in 12 min, 44–90% B in 2 min, followed by 4 min at 90% B. Peptides were ionized with a Nanospray Flex ion source equipped with a μPAC Flex iON Connector (Thermo) and a fused-silica emitter (20-μm inner diameter, 10-μm inner diameter of the tip, 360-μm outer diameter; MicroOmics Technologies), applying a +1.8-kV source voltage and an ion transfer tube temperature of 275 °C. Data were acquired in DIA mode. Each cycle comprised one MS1 survey scan (*m*/*z* 350–1,400, 120,000 resolution, RF lens 40%, AGC target 300%, maximum injection time 45 ms) followed by MS2 scans (profile mode, 30,000 resolution, RF lens 50%, AGC target 1,000%, maximum injection time 54 ms, variable window size with 1-*m*/*z* overlap).

Raw files were converted to mzML format as described above, and processed using the DIA-Umpire signal extraction module as implemented in FragPipe (v22.0) followed by MSFragger searches considering semi-specific cleavage (variable N terminus, C-terminal Arg for trypsin and C-terminal Asn or Asp for legumain-digested samples) with up to two missed cleavages, peptide length between 7 and 45 amino acids in a mass range of 200–5,000 Da, with up to five variable modifications per peptide. Dimethylation at protein N termini and Lys side chains (+32.0564 Da for trypsin, +34.0631 Da for legumain) and Cys carbamidomethylation (+57.0215 Da) were set as fixed modifications, and Met oxidation (+15.9949 Da), acetylation of peptide N termini and lysines (+9.9542 Da for trypsin, +7.9474 Da for legumain), N-terminal pyroglutamate formation from glutamine (−49.083 Da for trypsin, −51.090 Da for legumain) or glutamic acid (−50.067 Da for trypsin, −52.074 Da for legumain), and proline monomethylation (−16.028 Da for trypsin, −17.032 Da for legumain) as variable modifications. Peptide and protein identifications were filtered at 1% FDR at both the peptide-spectrum match and protein level using Philosopher. Quantification was performed with DIA-NN as implemented in FragPipe with unrelated runs enabled, N-terminal peptide intensities were used for downstream statistical analysis.

Peptide tables were filtered to retain peptides quantified in at least three of five replicates per condition; peptides consistently observed in only one condition (≥3 replicates) were also retained. N-terminally acetylated peptides and those starting beyond residue 120 were excluded, and N-terminal peptides with the same start position and N-terminal modifications, but different Met oxidation status were merged by summing intensities. To determine differentially abundant peptides between WT and ICP55^KO^ samples, peptide intensities were log_2_-transformed and analyzed using the LIMMA package, applying linear modeling with empirical Bayes moderation. Moderated *t* statistics and associated *P* values were calculated, FDR correction was applied, and log_2_ fold changes were computed from replicate means. Changes with a LIMMA-moderated, FDR-corrected *P* < 0.05 were considered significant.

### SILAC-based complexome profiling

For stable isotope labeling by amino acids in cell culture (SILAC)^68^, medium (Silantes) containing heavy (H) arginine (^13^C_6_/^15^N_4_; Arg10) and lysine (^13^C_6_/^15^N_2_; Lys8) and the light (L) variants (Arg0, Lys0) were used. Control HEK293T cells were cultured in heavy medium, and ICP55^KO^ cells were cultured in light medium. Efficient incorporation of heavy amino acids (95%) was verified by LC-MS. Mitochondria were isolated as described from heavy and light labeled cells (*n* = 4), then 75 μg of WT and 75 μg of ICP55^KO^ mitochondria were mixed and solubilized in 1% digitonin buffer. Samples were separated on a 4–13% BN gradient gel. The gel was cut into 44 pieces with 2.5-mm fraction length (*n* = 4).

### Complexome

#### Sample preparation and raw data processing

In-gel digestion was performed as described earlier^69,70^. Nanoflow LC-MS/MS analysis was performed using the Vanquish Neo system coupled to a QExactive HF instrument (Thermo Fisher Scientific). An in-house packed analytical column (75 μm × 200 mm, 1.9-μm ReprosilPur-AQ 120 C18 material (Dr. Maisch, Germany)) was used. The mobile phase solutions were prepared as follows: solvent A consisted of 0.1% FA, and solvent B contained 0.1% FA and 80% acetonitrile. Peptide separation was achieved using a 90-min linear gradient, starting at 4% solvent B and gradually increasing to 32% over the first 75 min, followed by a further increase to 49% over the next 15 min, and finally a wash step with 99% solvent B. The mass spectrometer was operated in data-dependent acquisition mode, automatically switching between MS and MS2. MS spectra (*m*/*z* 400–1,600) were acquired as described in ref. ^70^. The MS/MS spectra were searched against UniProt *H. sapiens* (UP000005640), and a customized contaminant database using Proteome Discoverer 3.1 with Sequest HT. The fragment ion mass tolerance was set to 0.02 Da and the parent ion mass tolerance to 5 ppm. Trypsin was specified as an enzyme. The following variable modifications were allowed: K (+8.0142), R (+10.00827), oxidation (M), deamidation (N, Q), acetylation (N-terminus), Met-loss (M) and a combination of Met-loss and acetylation (N terminus), and carbamidomethylation (C) was set as a fixed modification. Peptide quantification was done using a precursor ion quantifier node with the Top N Average (*n* = 3) method set for protein abundance calculation.

#### Data analysis

Raw abundances of 3,391 filtered proteins, each represented by at least two peptides and reported as master proteins within their respective protein groups, were normalized across individual fractions to correct for batch-specific effects. Zero values were replaced by missing values before normalization. Protein intensities within each fraction were median-normalized, and normalized values were recombined into a single data matrix for downstream analyses. This procedure ensured equal median intensities of corresponding fractions across all replicates and conditions. The dataset, arranged by increasing fraction number, was then processed using the PROMISed ShinyApp^71^.

Initial processing involved the removal of single occurrences of proteins that were not considered peaks, defined as intensities recorded in only one fraction. Protein profiles were smoothed using LOESS smoothing with a span value of 0.15. A detailed pipeline is available in ref. ^71^. The processed profiles were compiled into a dataframe, which is available as Supplementary Table 5. After processing, 2,989 protein profiles remained.

The dataset was then trimmed to include only previously reported mitochondrial proteins, resulting in 933 migration profiles (Supplementary Table 5). A reference list of mitochondrial proteins was acquired from ref. ^21^.

To assess reproducibility, pairwise distance metrics were computed for each protein migration profile across replicates and conditions. For each protein, migration profiles were extracted, and distances between sample pairs were calculated using the Manhattan metric. Comparisons were stratified into intra-group (within replicates of the same condition) and inter-group (between different conditions). Average intra- and inter-group distances were determined, and statistical significance was assessed using a two-sided *t*-test. This approach was based on methodology established by Schlossarek et al.^71^ and allowed for the identification of 177 proteins with significantly affected migration profiles (nominal *P* ≤ 0.05) in ICP55^KO^ mitochondria. To control for multiple hypothesis testing across all analyzed proteins, *P* values were adjusted using the Benjamini–Hochberg (BH) procedure, resulting in the identification of 115 proteins with significantly affected migration profiles (adjusted *P* ≤ 0.05). To further evaluate the robustness of these findings, we performed an exact permutation analysis by enumerating all possible assignments of the eight elution profiles into two groups (*n* = 70). For each permutation, the complete analysis pipeline, including distance calculation and statistical testing, was repeated. The number of significant proteins obtained under random label assignments was used to estimate the expected number of false positives and to derive an empirical FDR. Furthermore, an empirical global *P* value was calculated as the fraction of permutations yielding at least as many significant proteins as observed in the real dataset. Under random label assignments, an average of 30.5 (raw *P* value) and 8.5 (BH-adjusted) proteins were identified as significant, substantially fewer than observed in the real dataset. This corresponds to an empirical FDR of ~17% (raw *P* values) and 7% (BH-adjusted). Importantly, the number of significant proteins observed in the real dataset exceeded those obtained in permuted datasets in nearly all cases (66/69), yielding an empirical global *P* value of 0.0435.

Protein oligomeric state analysis was performed by pooling reproducible migration profiles using the PROMISed ShinyApp^71^. Replicates with Pearson correlation ≥ 0.7 were pooled, and single, non-reproducible replicates were excluded. Summed migration profiles were then deconvoluted using previously established tools^72^, with the following parameters: candidate peaks were required to reach ≥10% of the maximum profile intensity; the minimum drop and rise in intensity between adjacent peaks was set to >20% of the preceding peak’s intensity; and peak boundaries were defined until the signal decreased to ≤5% of the maximum intensity.

Apparent molecular weights of proteins (AppMW) were estimated from the BN–PAGE protein markers by identifying, for each deconvoluted peak, the fraction with the highest intensity and mapping this fraction to its corresponding estimated molecular weight. Theoretical protein masses (ThMW) were obtained from UniProt accessions and used to compute the oligomeric state ratio (OSR = AppMW/ThMW) for each peak. Analyses were performed separately for WT and ICP55^KO^ genotypes. Peaks with OSR ≥ 2 were classified as complex-associated^72,73^.

Trajectory plots were generated by pairing per-peak protein abundances between WT and ICP55^KO^. For each protein peak with measurements in both WT and ICP55^KO^, we assigned a trend label (upregulated in ICP55^KO^, or downregulated in ICP55^KO^). Individual trajectories were drawn by connecting the two genotype points for each protein peak; points were colored by genotype and scaled by the OSR. Median abundance per genotype was overlaid as a summary line. Protein peaks unique to one genotype were depicted as points.

Protein migration profiles visualized as line plots were prepared using ggplot2 R package. Fractions were mapped to calibrated molecular weights (kDa) using BN–PAGE marker proteins. For each condition and fraction, the mean intensity and its standard error (s.e. = s.d./√*n*) were computed and plotted as solid lines with ±s.e. ribbons, respectively. Migration profiles were scaled genotype-wise to a common 0–1 range.

Sub-mitochondrial localizations were assigned using MitoCarta3.0 annotations^21^. Proteins were grouped according to their annotated compartment, and distributions were visualized as pie charts for three datasets: the full MitoCarta3.0 reference, proteins detected in the complexome, and proteins with affected migration profiles. For each dataset, categories representing <1% of proteins were consolidated into ‘Other’. Percentages were calculated from protein counts and sorted in descending order for clearer visualization.

Heatmaps were produced using ComplexHeatmap R package^74^ using Euclidean distance and complete linkage for row clustering.

A Venn diagram was created using the ggVennDiagram R package, and the histogram displaying the distribution of the OSR per protein was created using the ggplot2 R package.

#### GO-terms enrichment analysis

The GO-terms enrichment analysis of the 177 altered proteins was performed using the online version of ShinyGO^75^. For the GO-terms enrichment analysis, 933 proteins were used as a background to correct for the enrichment of mitochondrial proteins during sample preparation. The results demonstrate representative enriched GO terms and were visualized as lollipop plots using ggplot2 R package^76^.

### Integration of N-terminomics and complexome data

To explore the potential influence of the disruption of ICP55 activity on complex assembly, we performed an intersection analysis between the combined list of HUNTER-validated (Supplementary Table 3) and predicted (via TargetP and MitoFates; Supplementary Table 1) ICP55 substates, and protein members of complexes in public databases. The list of complex members was obtained from a combination of the CORUM^77,78^ and STRING^34^ databases, only including complexes described with physical evidence.

### Statistics, data analysis and reproducibility

All experiments were independently replicated at least three times. Quantitative data are presented as mean ± standard error of the mean (s.e.m.). Statistical significance was assessed using unpaired, two-sided Student’s *t*-tests for comparisons between two groups and one-way ANOVA for multiple group comparisons. Statistical analyses were performed using Microsoft Excel, and graphs were generated with GraphPad Prism version 10.6.1 (GraphPad Software). Western blot and autoradiography images were processed using ImageJ v1.53a and Adobe Photoshop 2025 (Adobe). Details on replicate numbers and statistical tests are provided in the corresponding figure legends.

### Reporting Summary

Further information on research design is available in the Nature Portfolio Reporting Summary linked to this Article.

## Online content

Any methods, additional references, Nature Portfolio reporting summaries, source data, extended data, supplementary information, acknowledgements, peer review information; details of author contributions and competing interests; and statements of data and code availability are available at https://doi.org/10.1038/s41594-026-01876-7.

## Supplementary information

Reporting Summary

Peer Review File

Supplementary Table 1 **Prediction of mitochondrial targeting sequences and processing sites**. All proteins in the UniProt human proteome for which at least one of the prediction tools, MitoFates or TargetP 2.0, identified a mitochondrial targeting sequence (MTS) and corresponding cleavage site are listed.

Supplementary Table 2 Quantitative N-terminome analysis of mitochondria from HEK293T wild-type and ICP55^KO^ cells. N-terminal peptides derived from mitochondrial proteins are listed if quantified in at least three of five replicates per condition or consistently detected in only one condition (≥3 replicates). Peptides were mapped to the first 120 amino acids of the corresponding proteins. The table includes quantitative information for each peptide in each measurement, average intensity for each condition, and the comparison (fold change) with FDR-adjusted *P* values, and associated annotation information.

Supplementary Table 3 Experimentally determined high-confidence ICP55 substrate spectrum. The table lists all proteins identified as ICP55 substrates by the N-terminome data presented in Supplementary Table 2. Unknown, no protein sublocalization reported in MitoCarta3.0.

Supplementary Table 4 Quantitative proteome analysis mitochondria isolated from HEK293T wild-type and ICP55^KO^ cells. Listed are all mitochondrial proteins (defined as contained in MitoCarta3.0) with quantitative information in each run, average intensity for each condition, and the comparison (fold change) with *t*-test, *P* values and FDR-adjusted *P* values.

Supplementary Table 5 SILAC-based complexome profiling. The table contains filtered and smoothed protein profiles sorted according to decreasing MW. Migration profiles were acquired for four biological replicates for WT and ICP55^KO^ mitochondria. To determine affected protein complexes, changes in protein migration profiles were determined using the Manhattan distance metric, and significance was determined using a two-sided *t*-test. Resulting *P* values were adjusted for multiple testing using the Benjamini–Hochberg correction.

## Source data

Source Data Fig. 1 Statistical source data.

Source Data Fig. 2 Statistical source data.

Source Data Fig. 3 Statistical source data.

Source Data Fig. 4 Statistical source data.

Source Data Fig. 5 Statistical source data.

Source Data Fig. 6 Statistical source data.

Source Data Extended Data Fig. 1 Statistical source data.

Source Data Extended Data Fig. 2 Statistical source data.

Source Data Extended Data Fig. 3 Statistical source data.

Source Data Extended Data Fig. 4 Statistical source data.

Source Data Extended Data Fig. 5 Statistical source data.

Source Data Extended Data Fig. 6 Statistical source data.

Source Data Extended Data Fig. 7 Statistical source data.

Source Data Extended Data Fig. 8 Statistical source data.

Source Data Figs. 1–3 and 6 and Extended Data Figs. 4, 5 and 10 Unprocessed western blots and gels.

## Data Availability

MS/MS data are deposited to the ProteomeXchange Consortium via the PRIDE repository (https://www.ebi.ac.uk/pride) with the dataset identifiers PXD070326 for the N-terminome data, PXD070247 for the proteome data and PXD068519 for the quantitative complexome data. Source data are provided with this paper.

## Code availability

The code and supporting files used for the Complexome analysis have been deposited to the GitHub repository (https://github.com/Marcin-Luzarowski/CF-MS-ICP55.git). The following is a complete list of R packages used in the project: R version 4.4.1; R packages: tidyverse (v2.0.0), ggplot2 (v3.5.1), ggpubr_(v0.6.0), dplyr (v1.1.4), tidyr (v1.3.1), readr (v2.1.5), purrr (v1.0.2), tibble (v3.2.1), stringr (v1.5.1), forcats (v1.0.0), lubridate (v1.9.3), ggrepel (v0.9.5), plotly (v4.10.4), ComplexHeatmap (v2.20.0), circlize (v0.4.16), ggVennDiagram (v1.5.2), patchwork (v1.2.0), scales (v1.3.0), pastecs (v1.4.2), tidytext (v0.4.3), RColorBrewer (v1.1-3) and ShinyApp PROMISed (v1.0.2).
