# Supplementary material for: A single-amino-acid cleavage controls global mitochondrial complex integrity
Source: Nat Struct Mol Biol. 2026 Aug 31;33(10):1535–47. doi: 10.1038/s41594-026-01876-7 (PMC13672106; doi:10.1038/s41594-026-01876-7)

Figure 1a

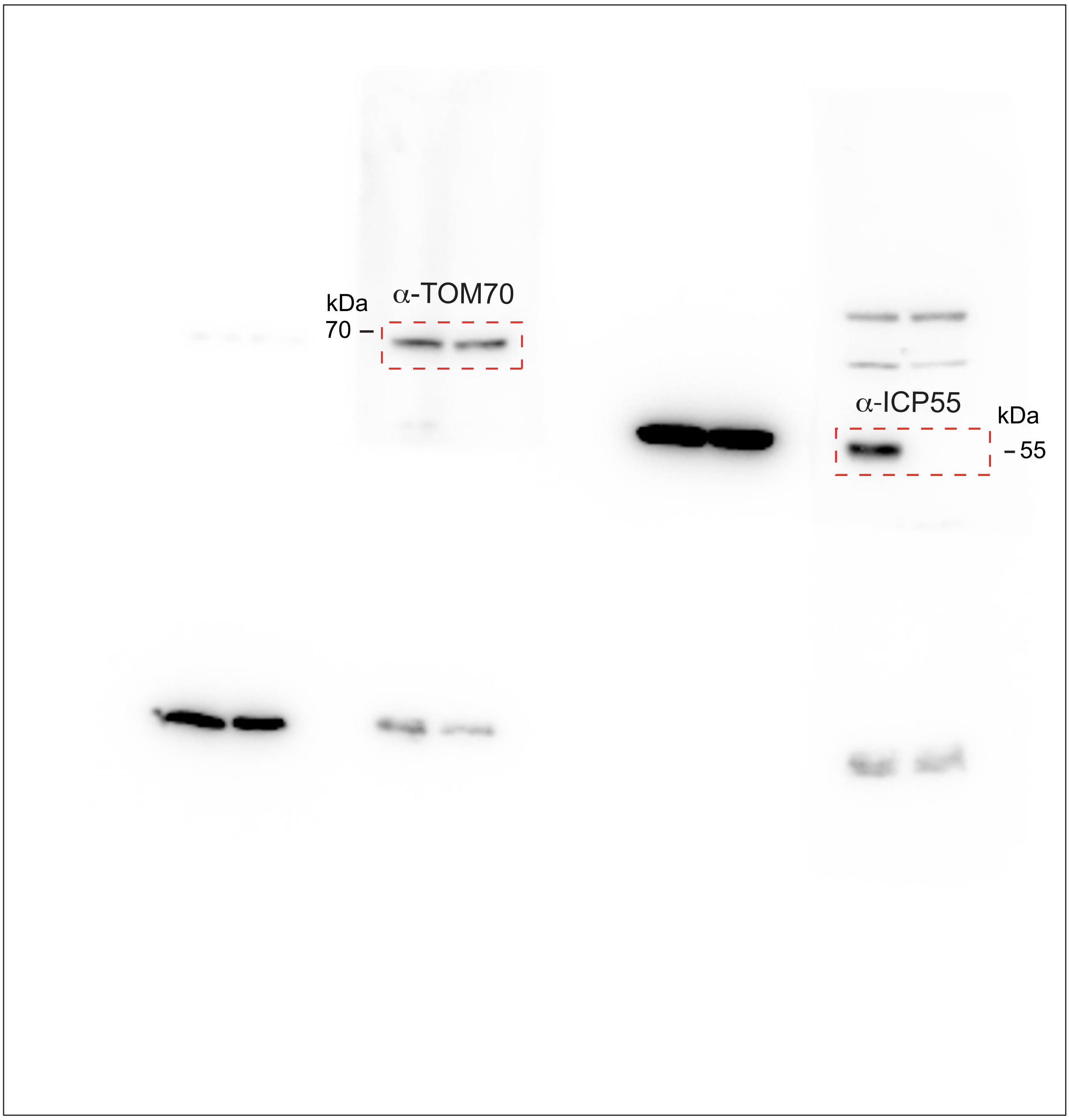

Figure 1g

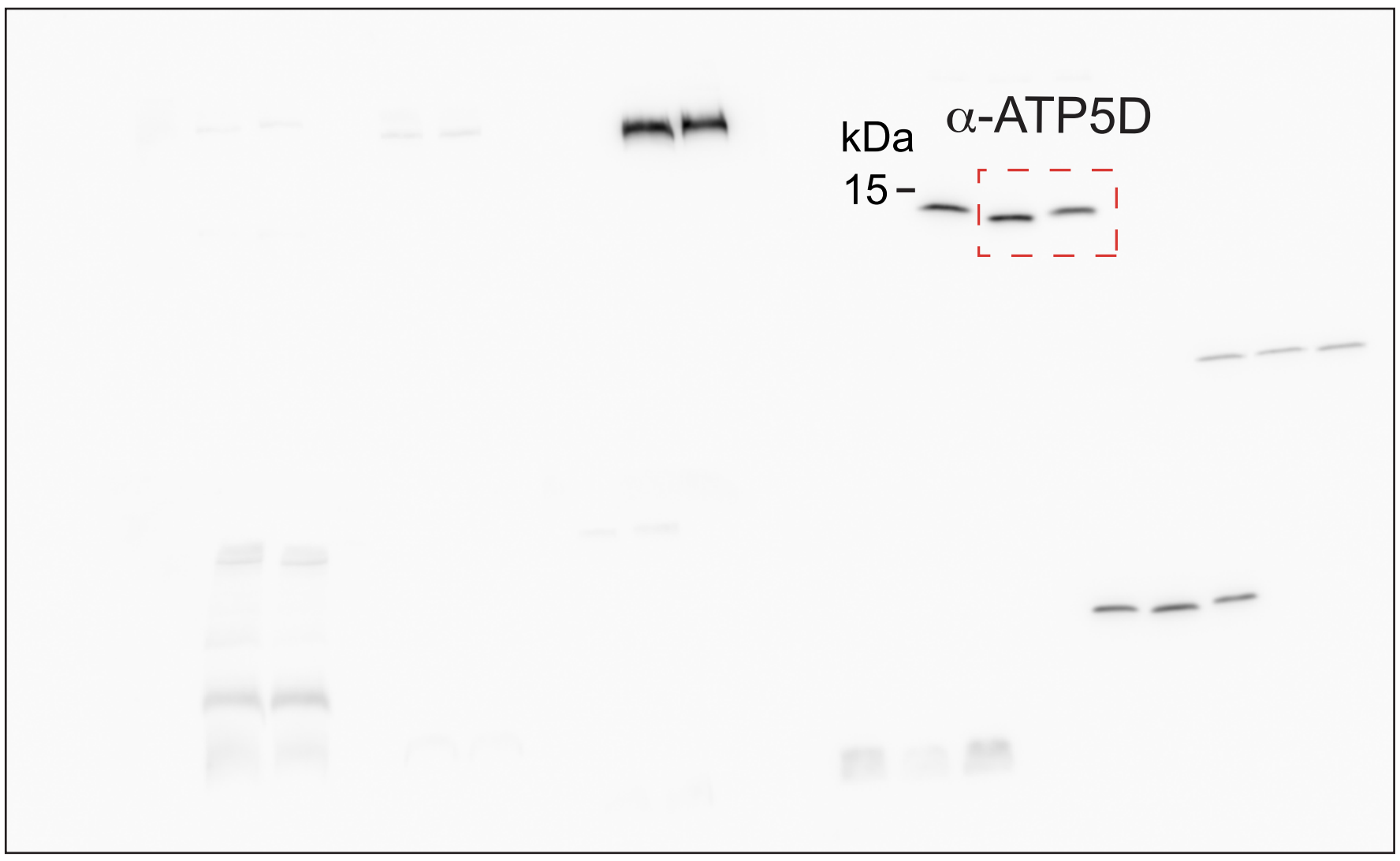

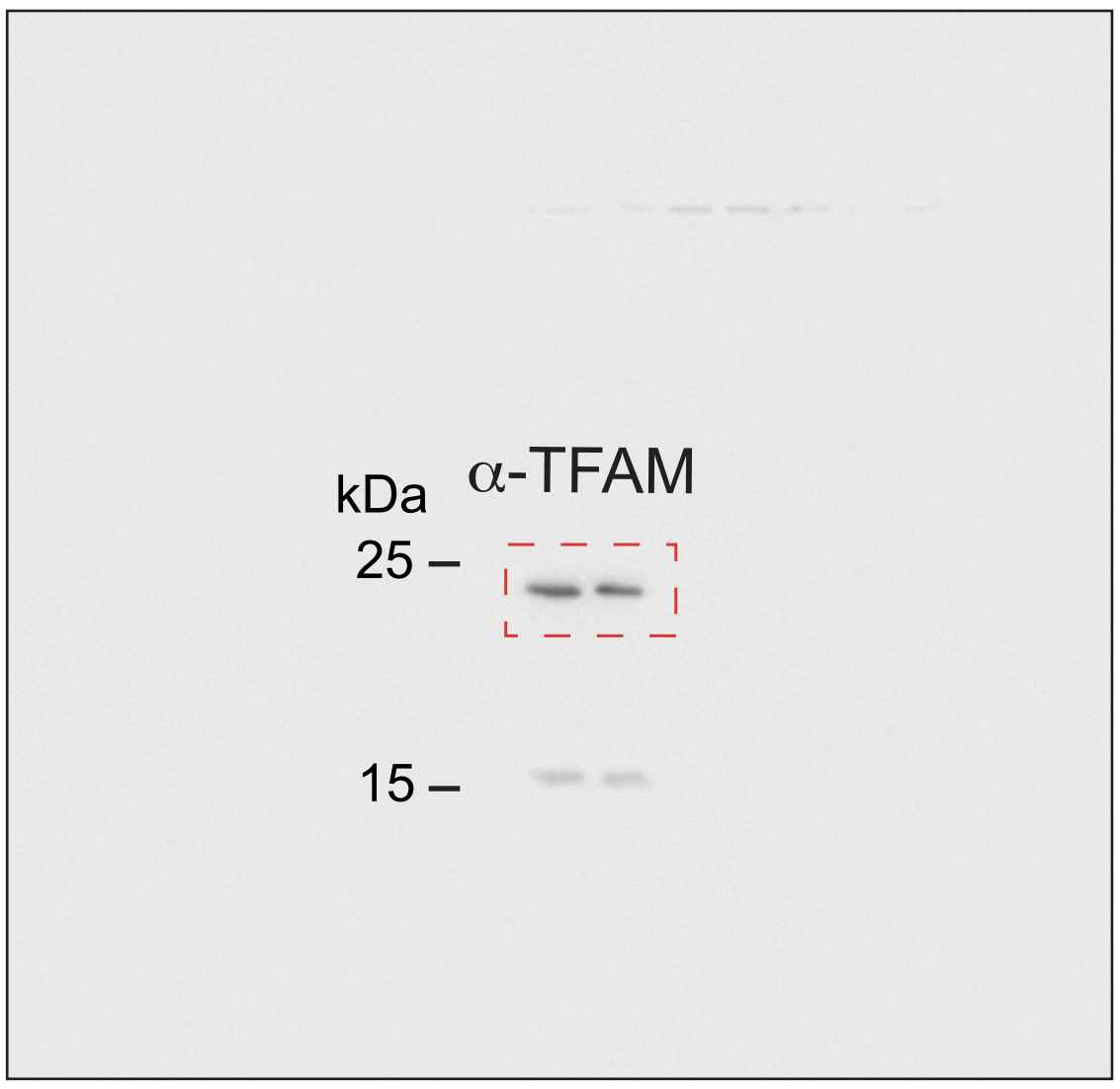

Figure 1g

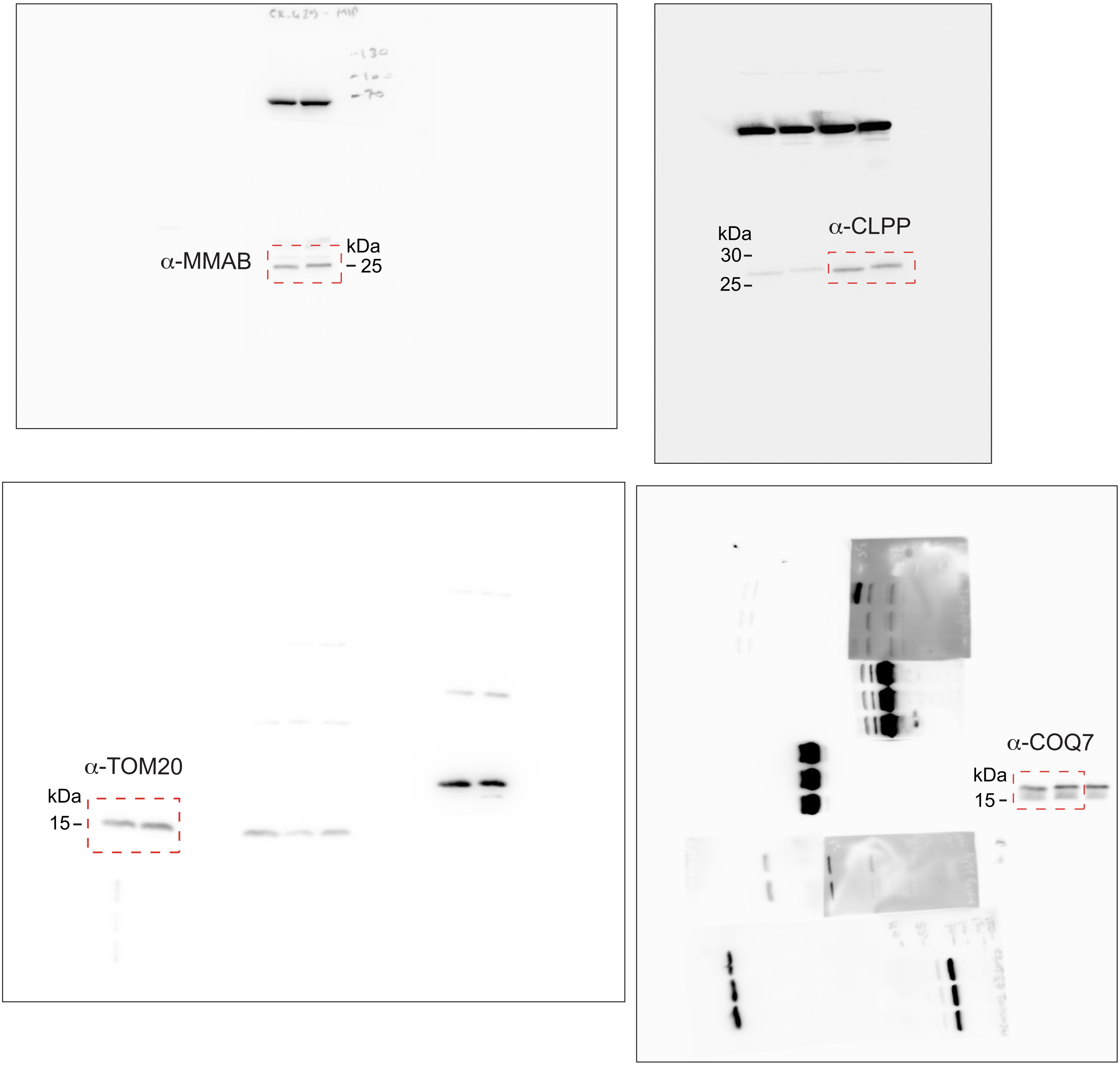

Figure 1h

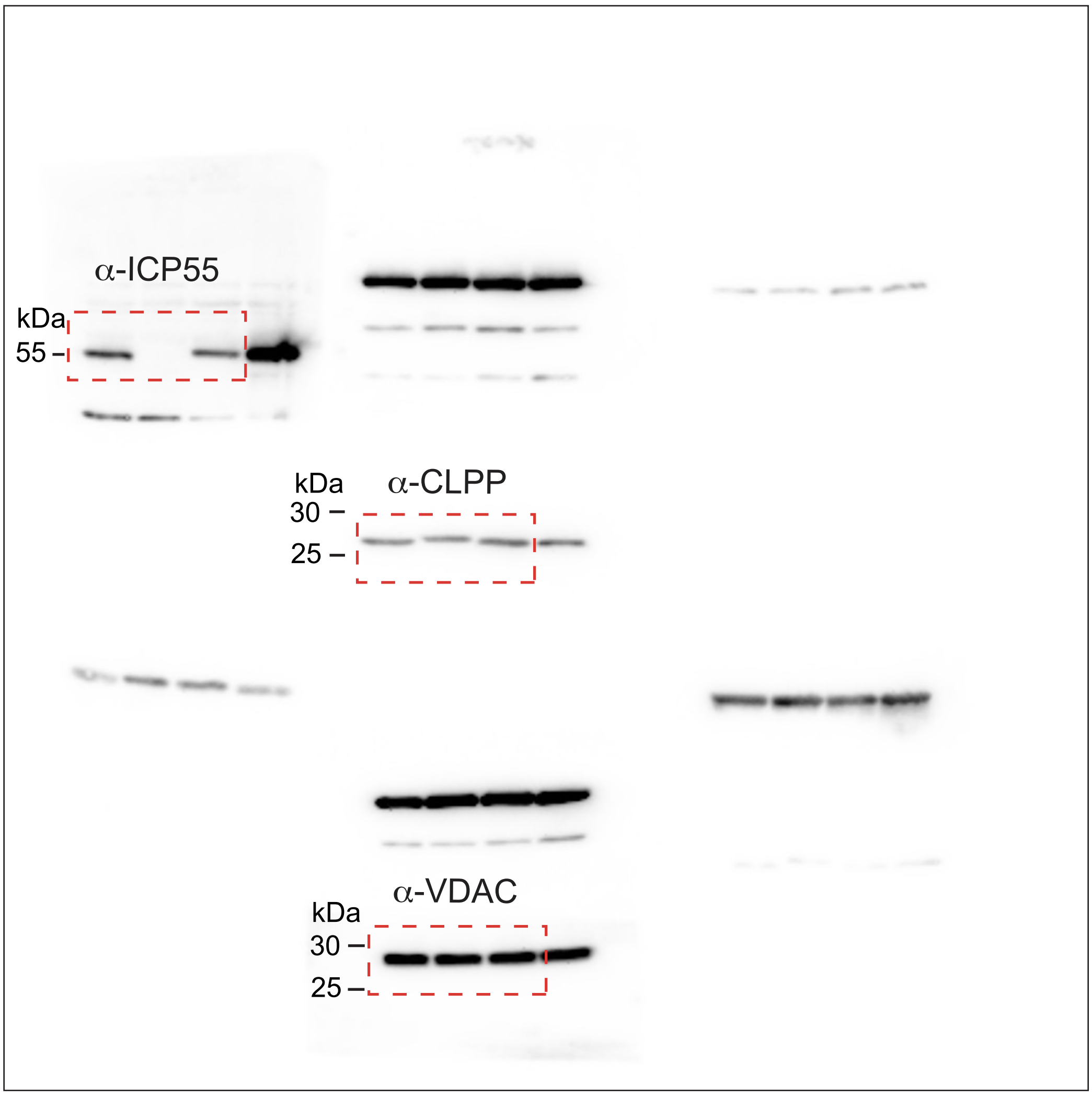

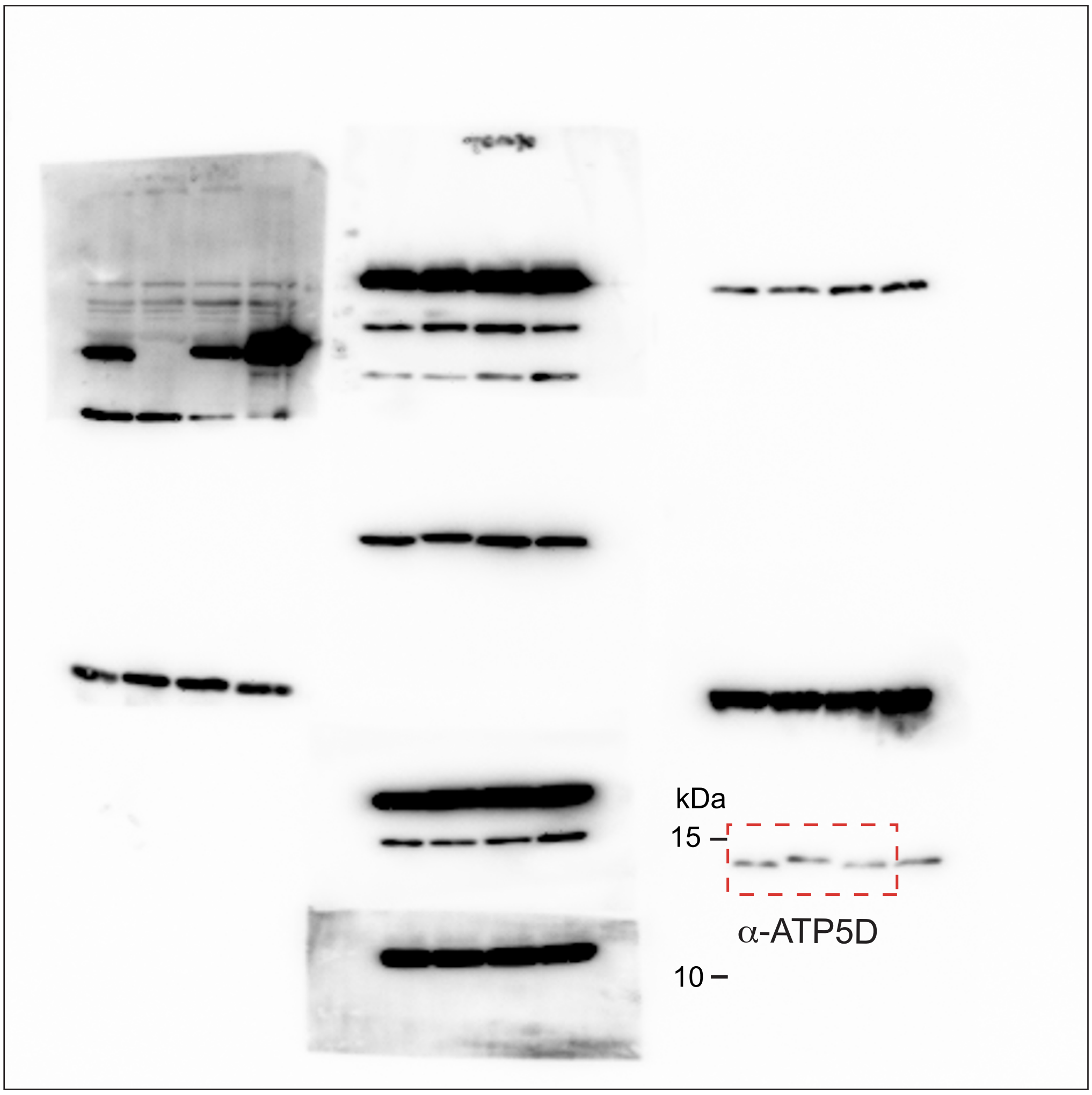

Figure 2b

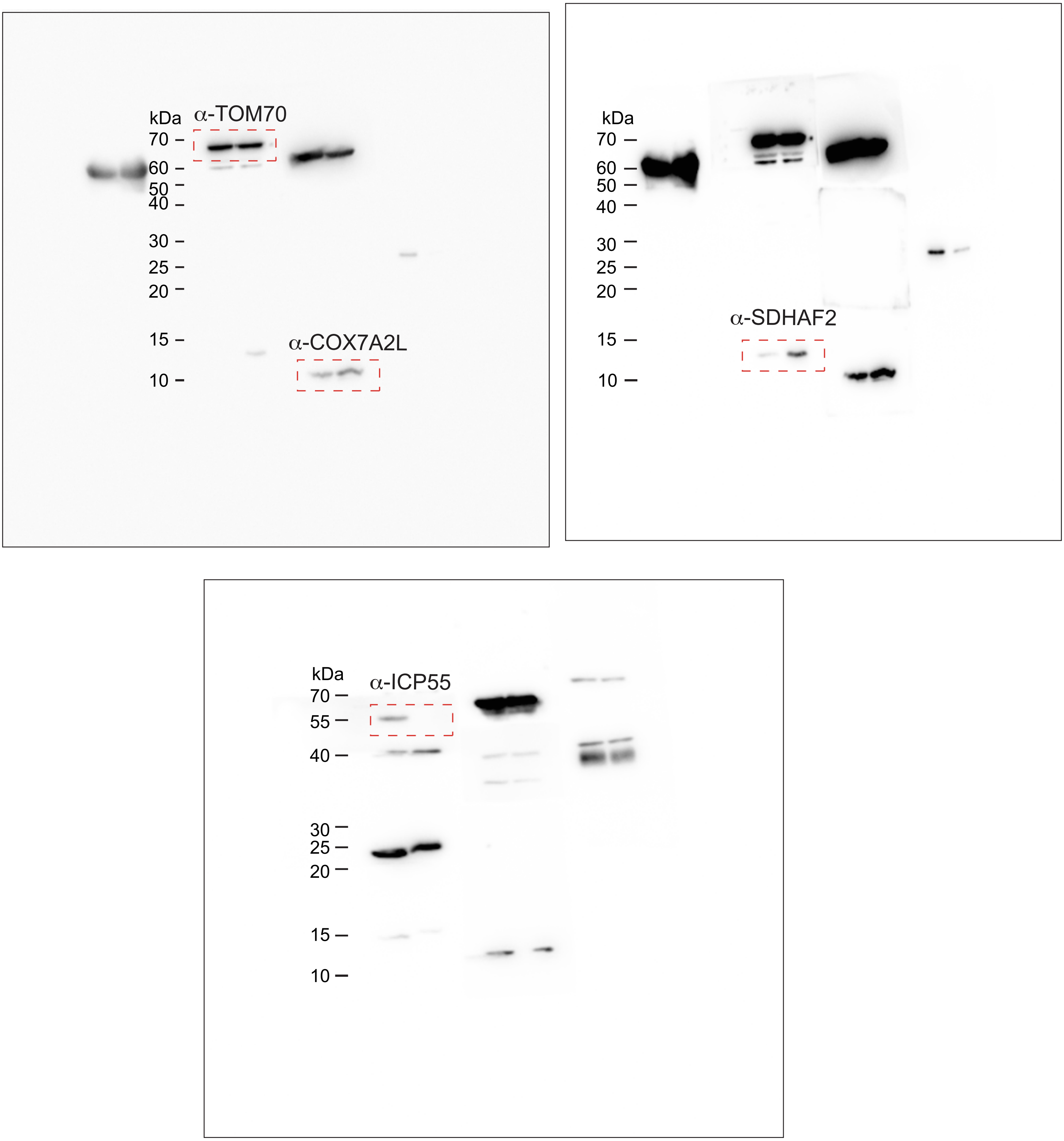
kDa
α-TOM70
70
60
50
40
30
25
20
15
α-COX7A2L
10
kDa
70
60
50
40
30
25
20
α-SDHAF2
15
10
kDa
α-ICP55
70
55
40
30
25
20
15
10

Figure 2c

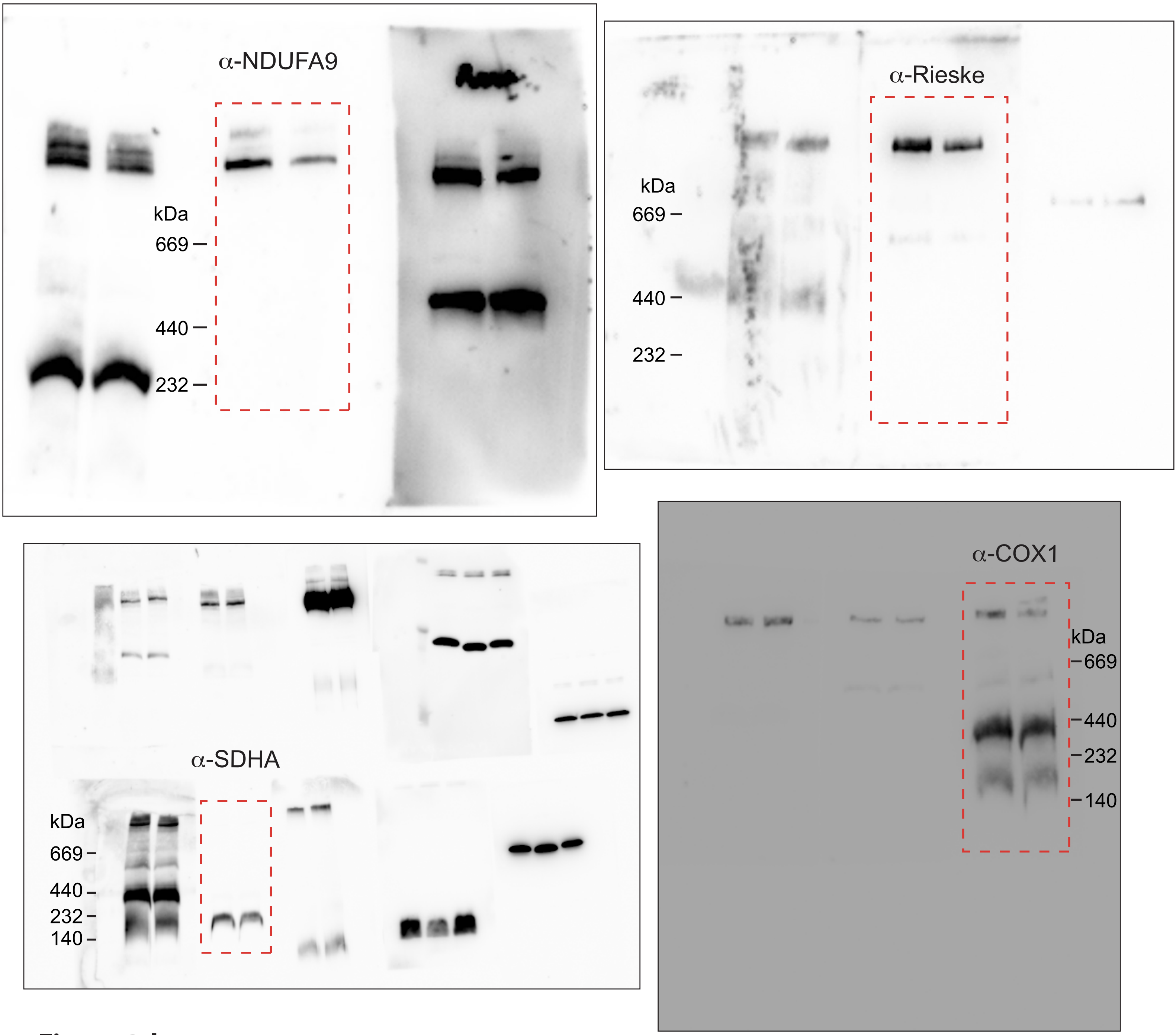

Figure 2d

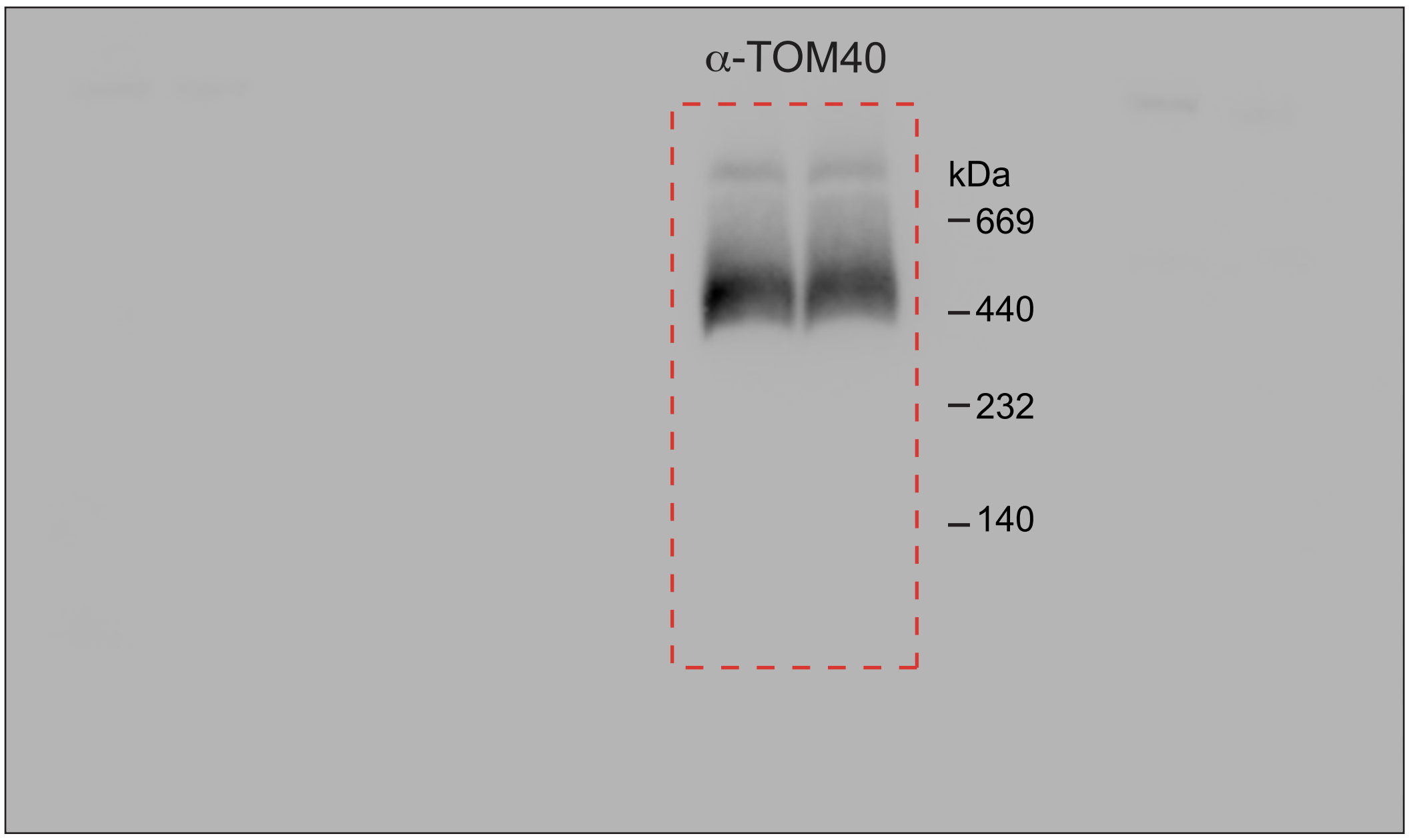

Figure 2g

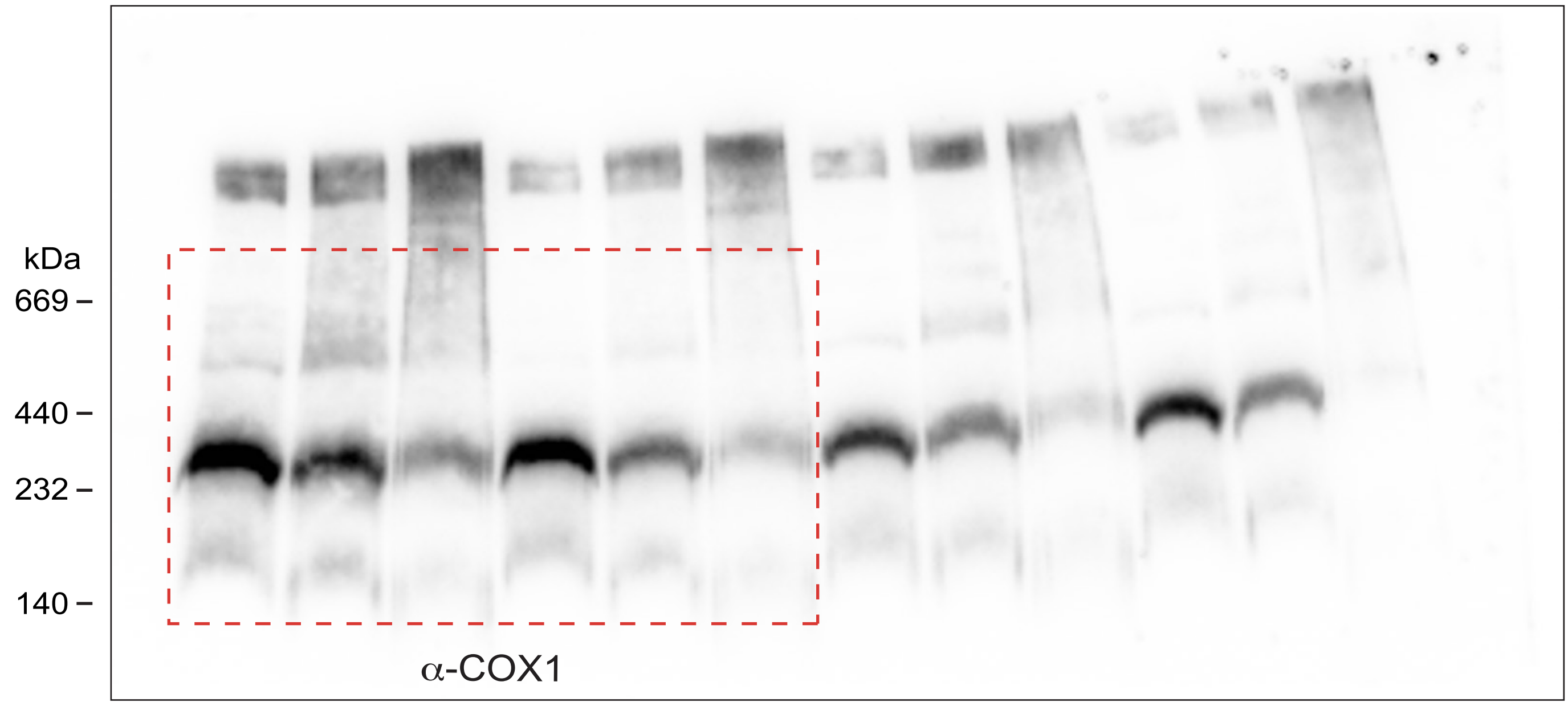

Figure 3b

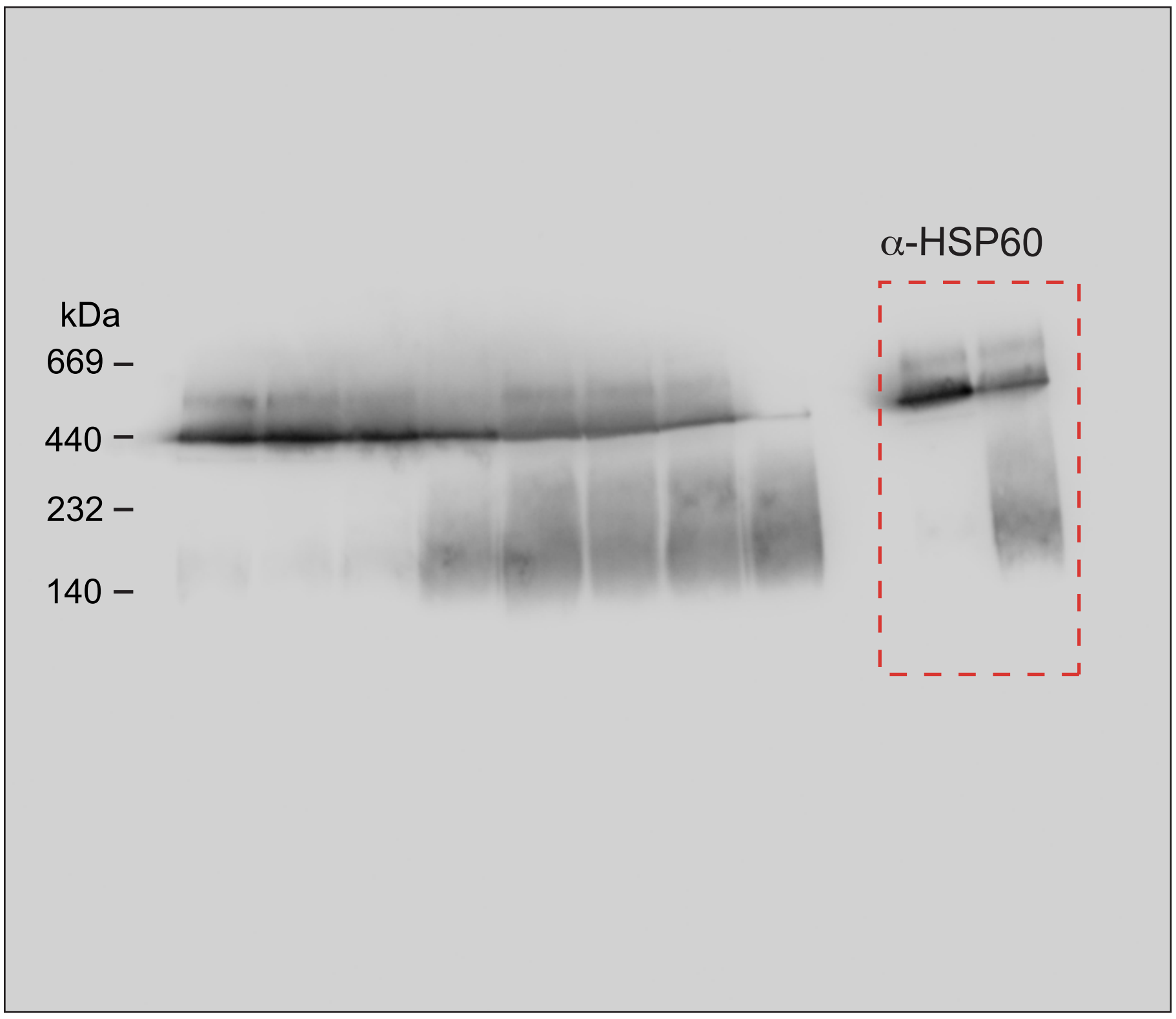

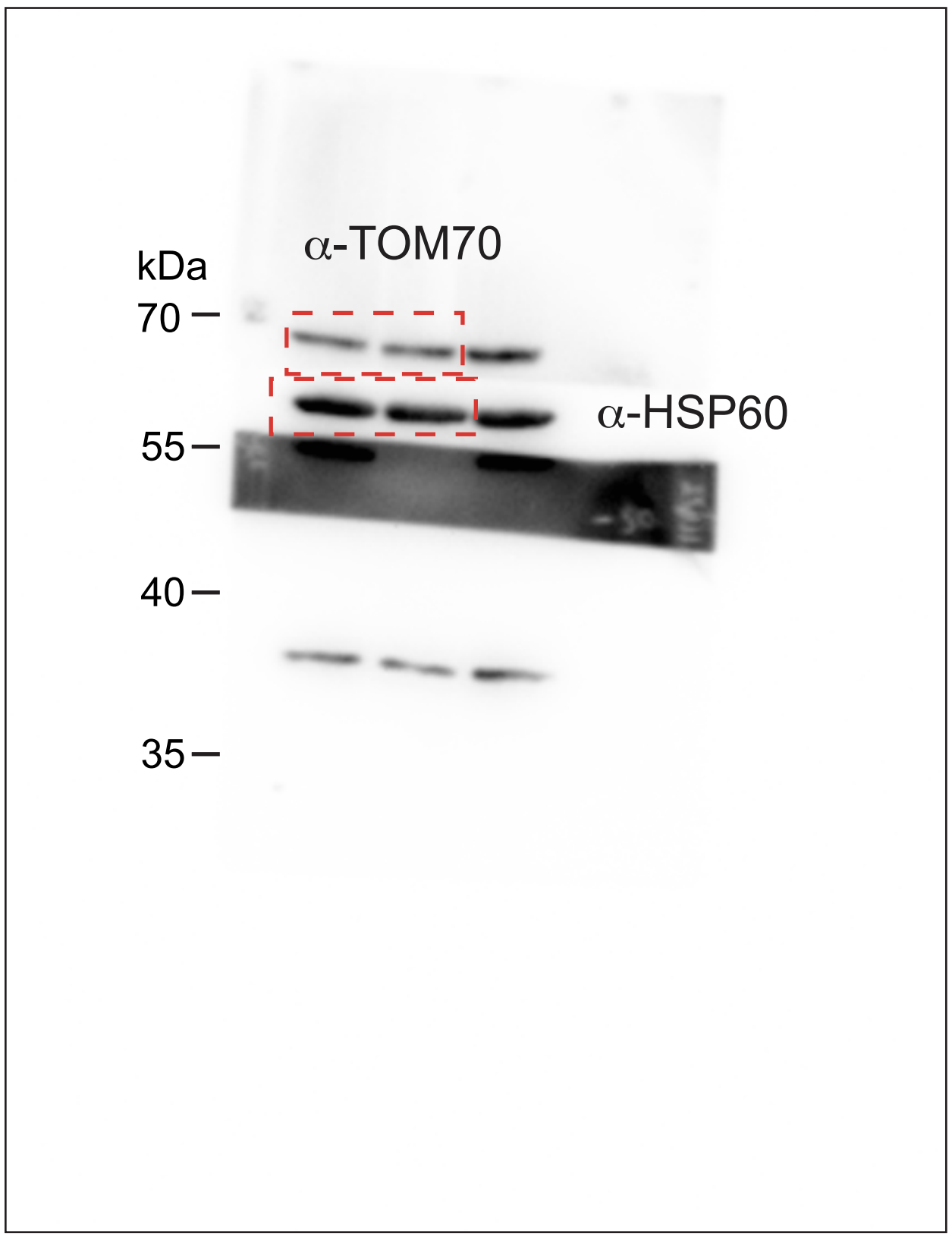

Figure 3d

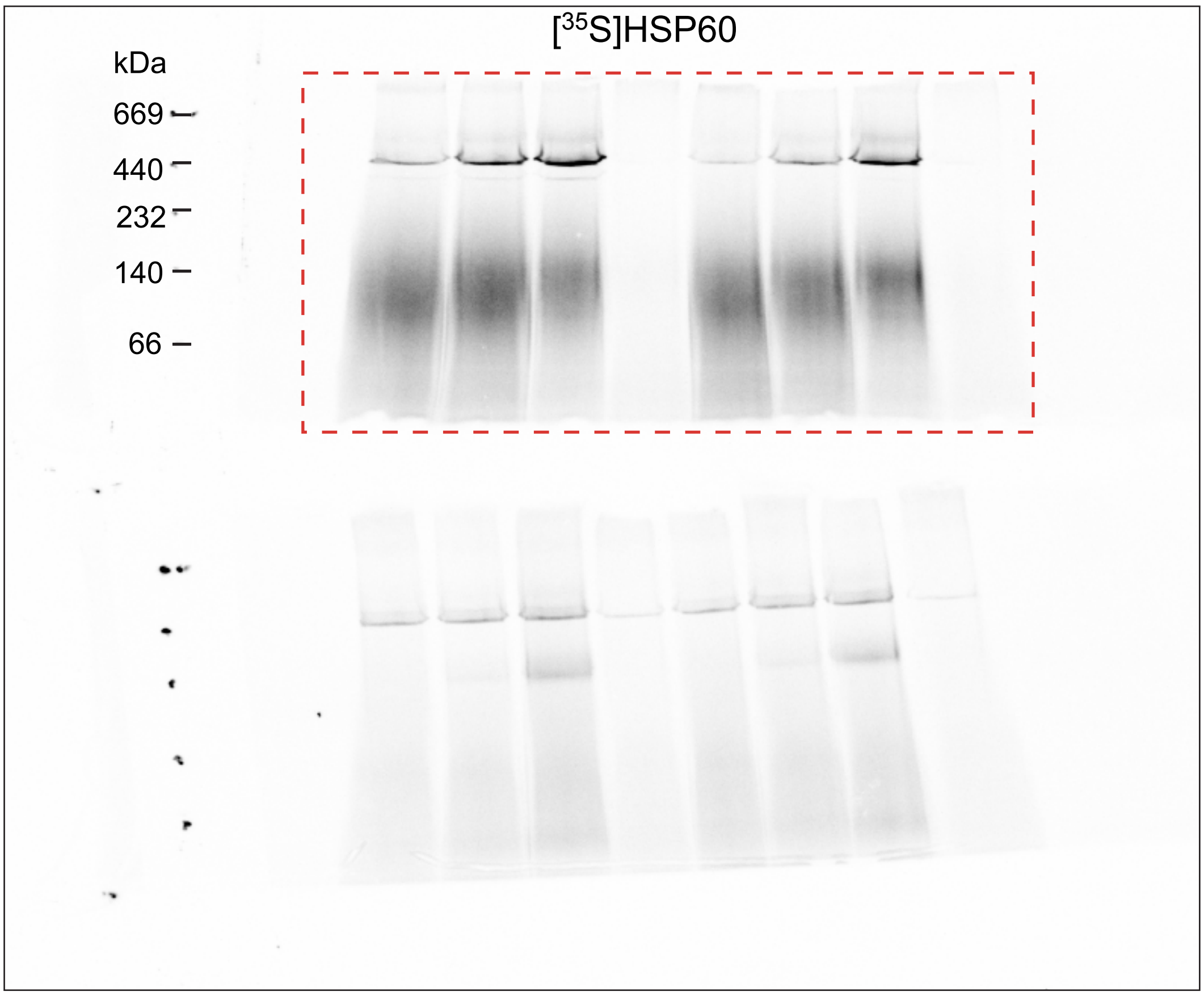

Figure 3f

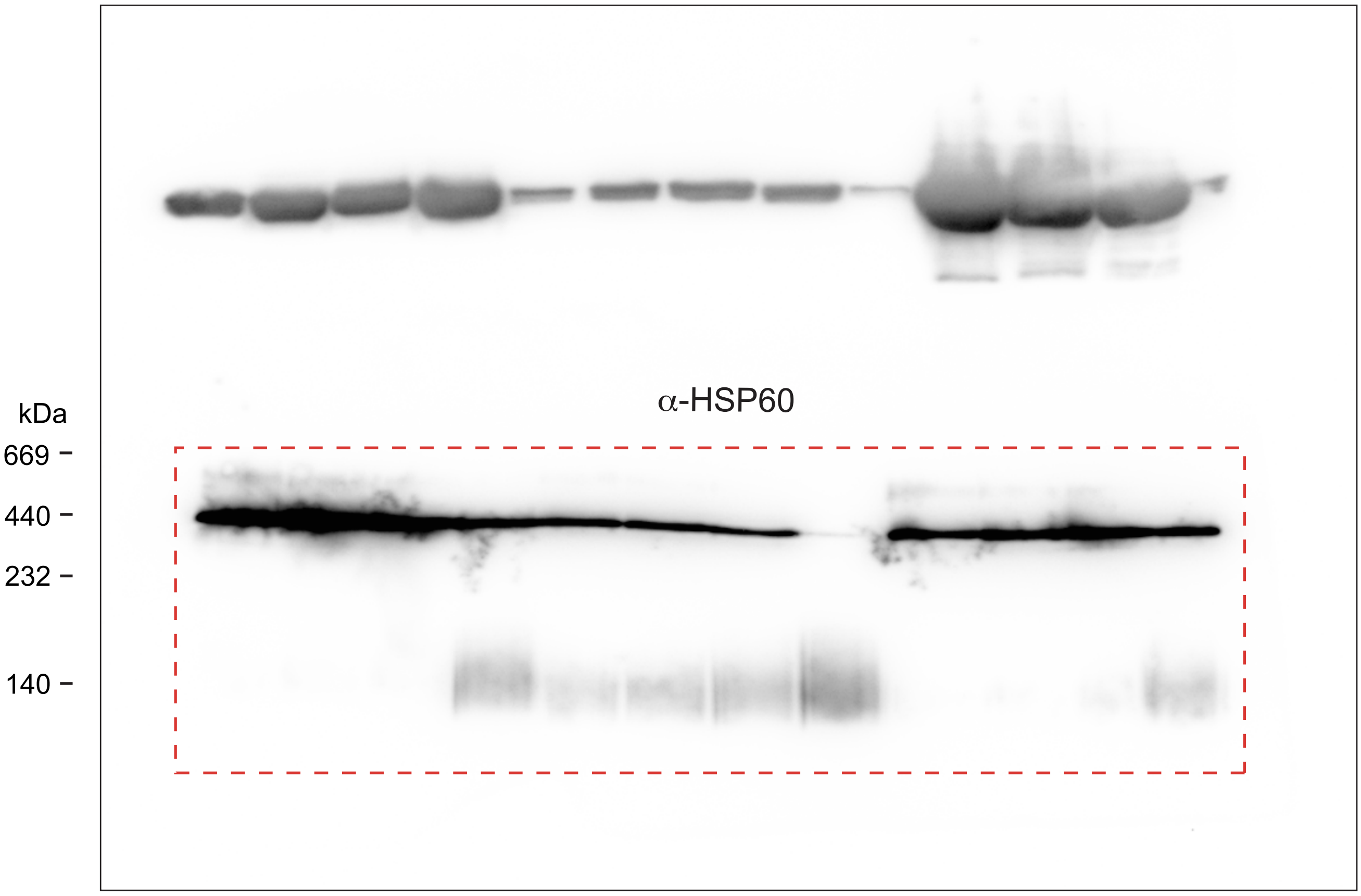

Figure 6b

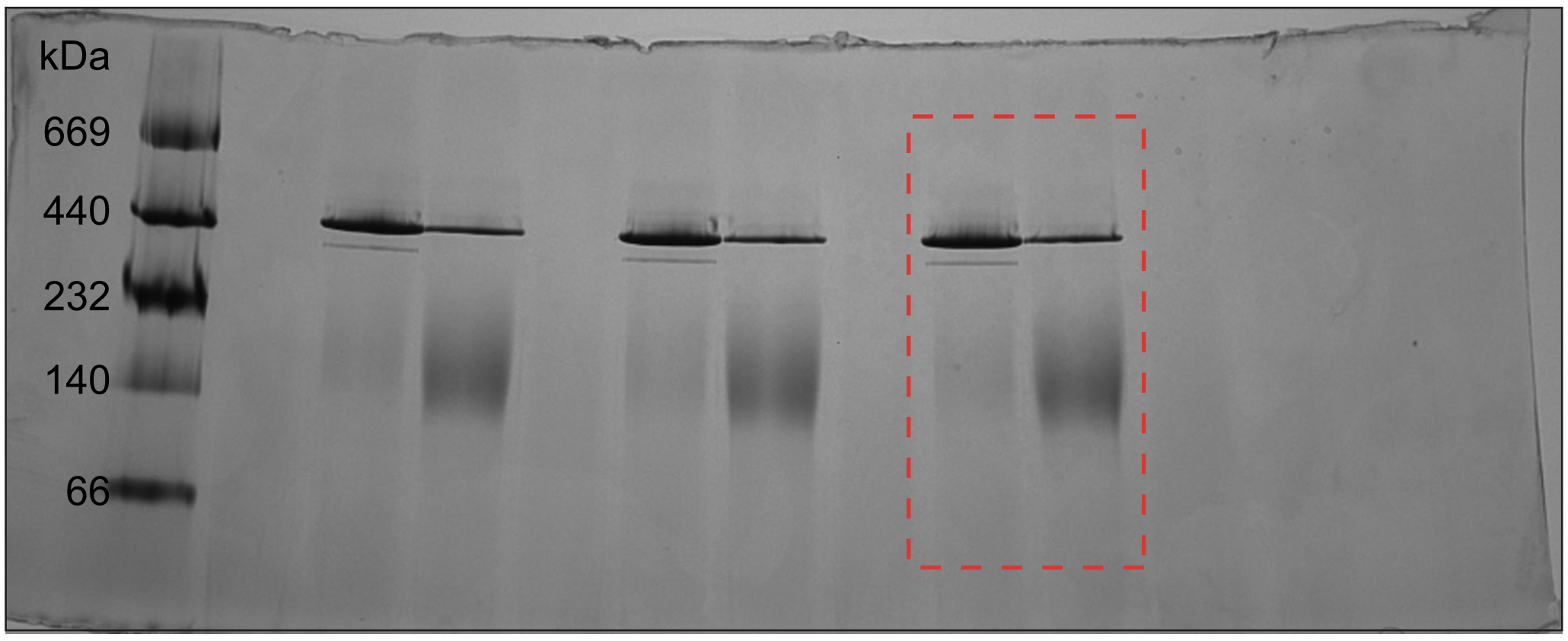

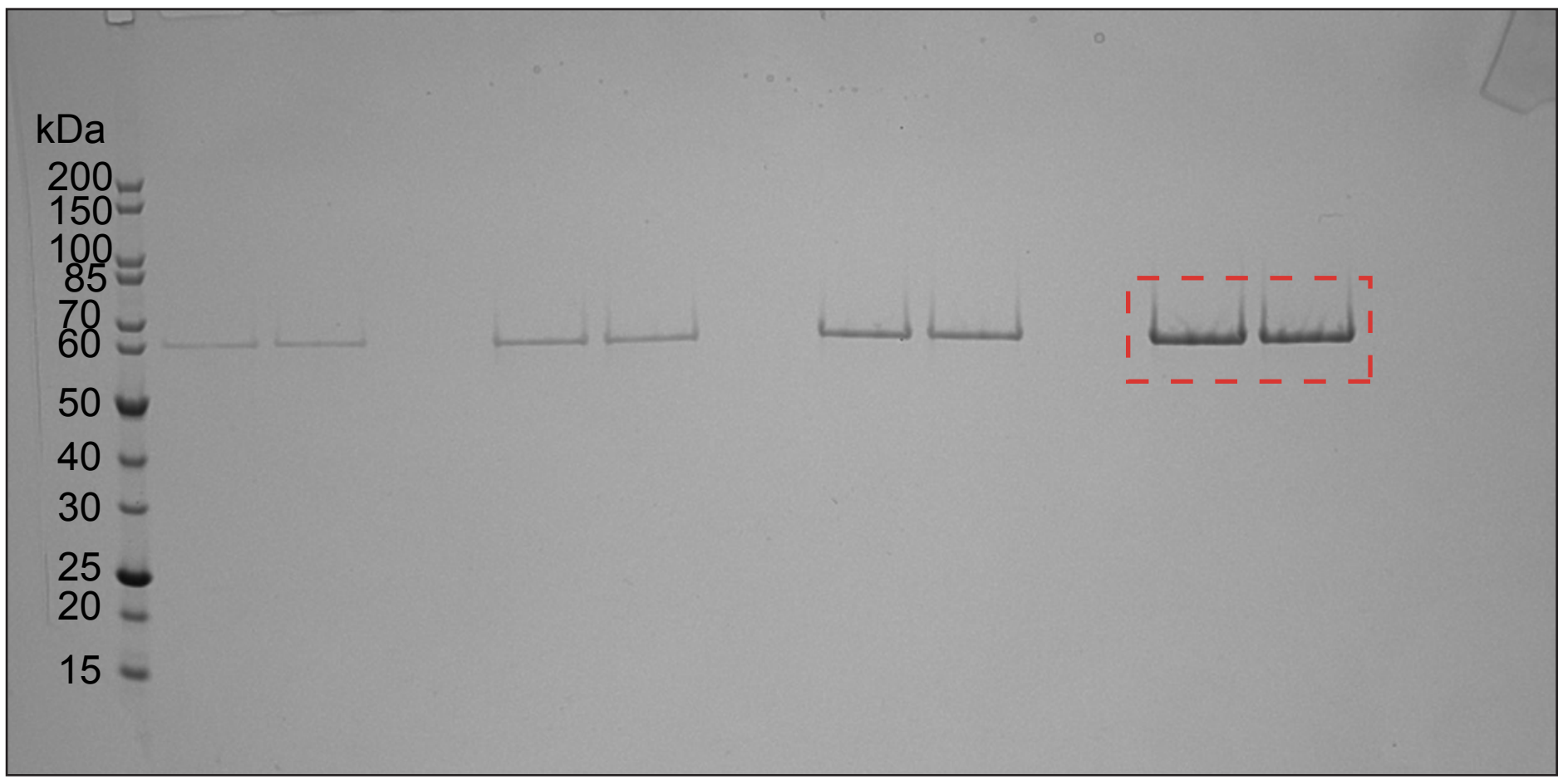

Figure 6d

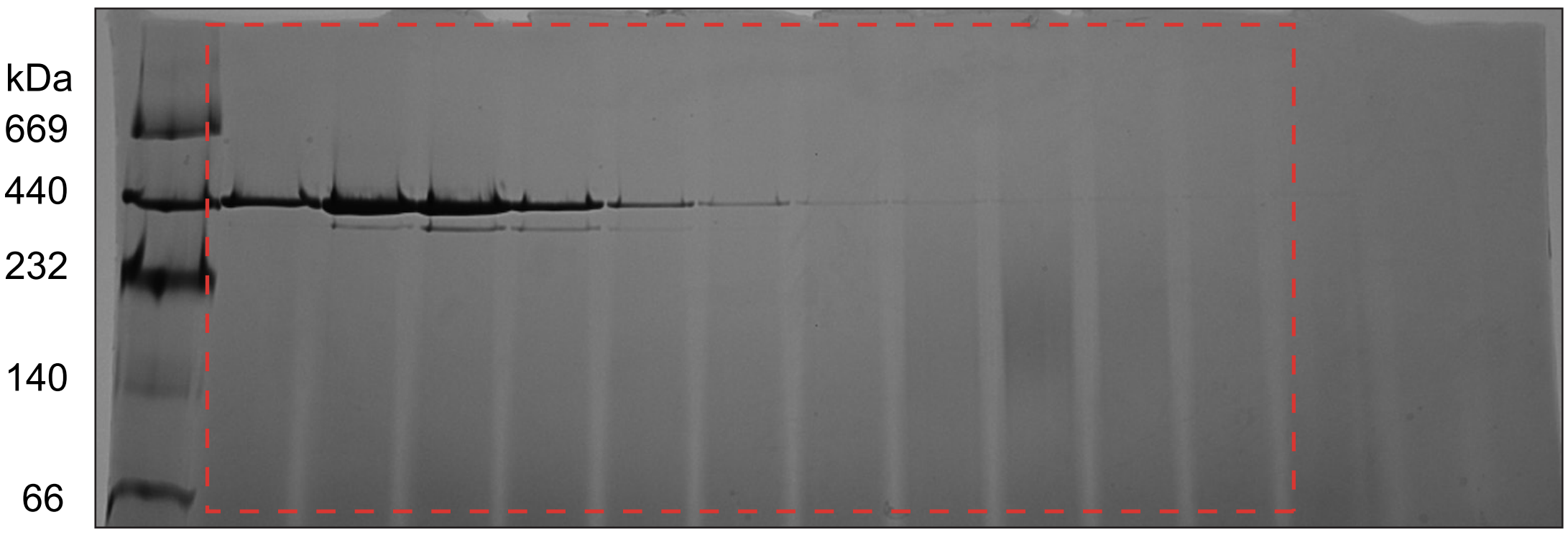

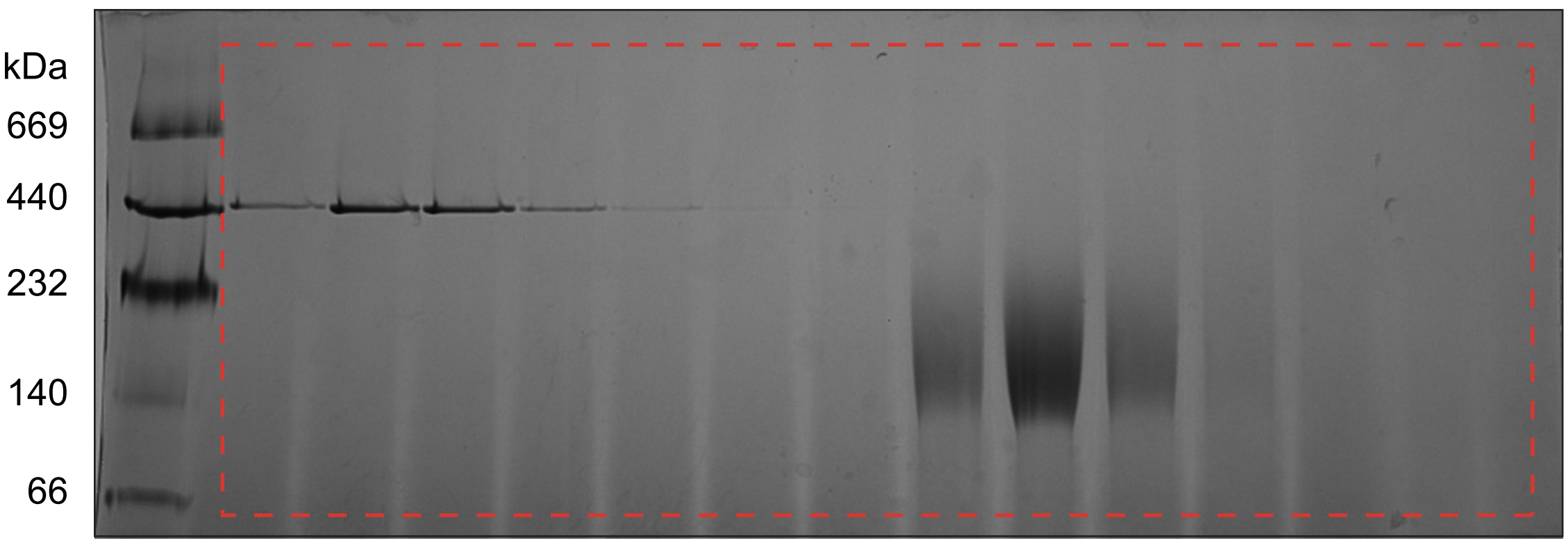

Extended Data Figure 4a

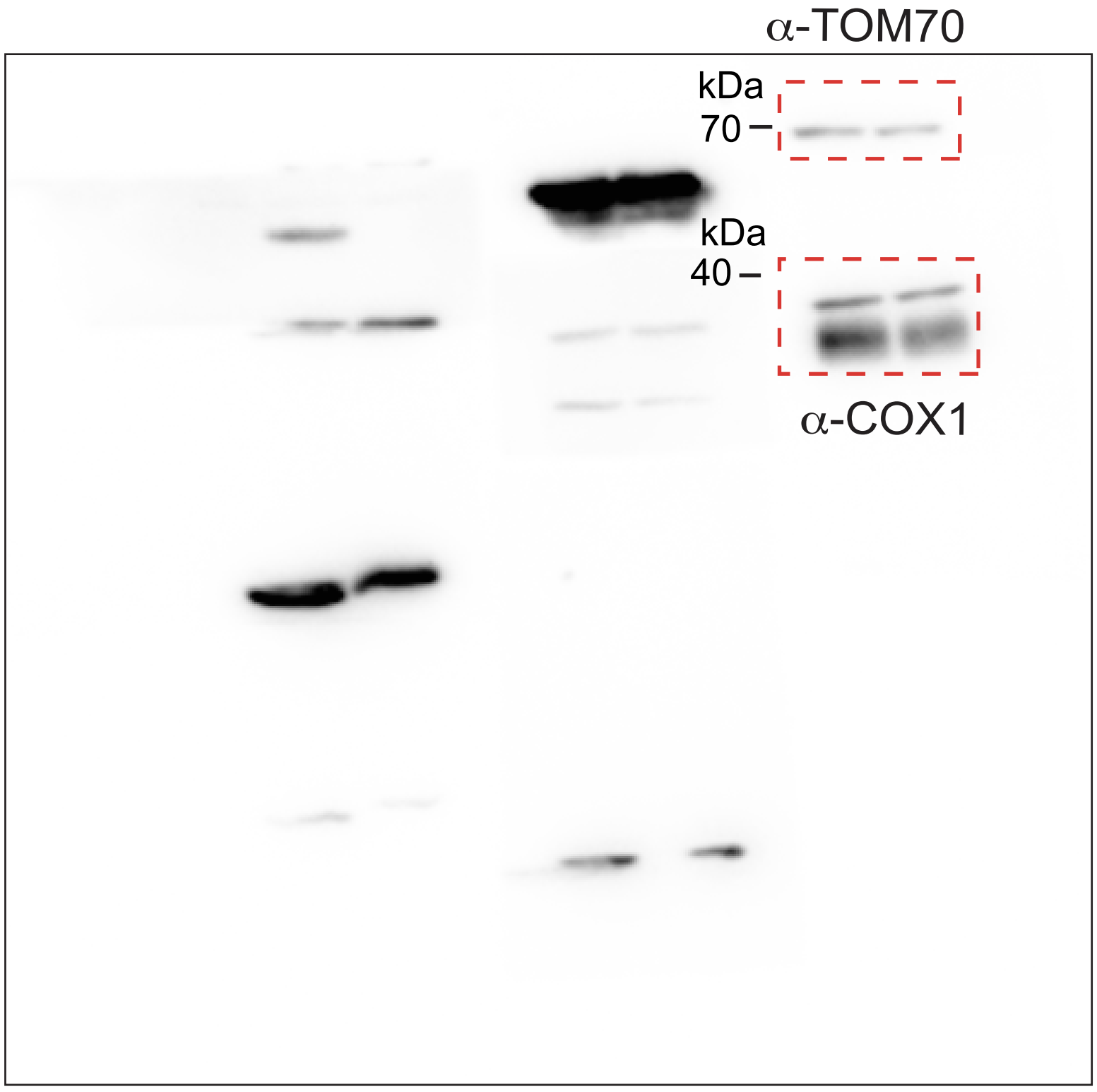

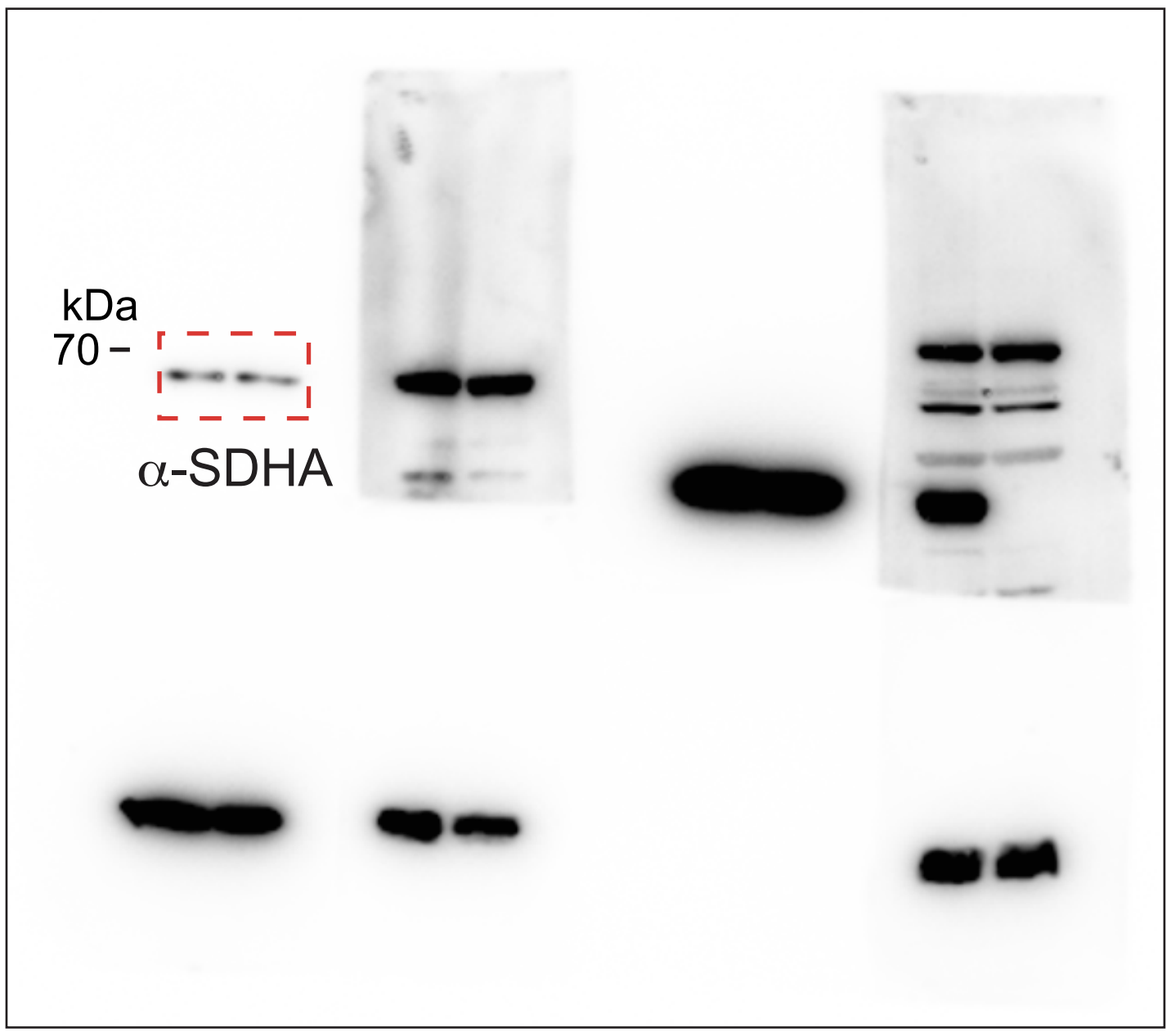

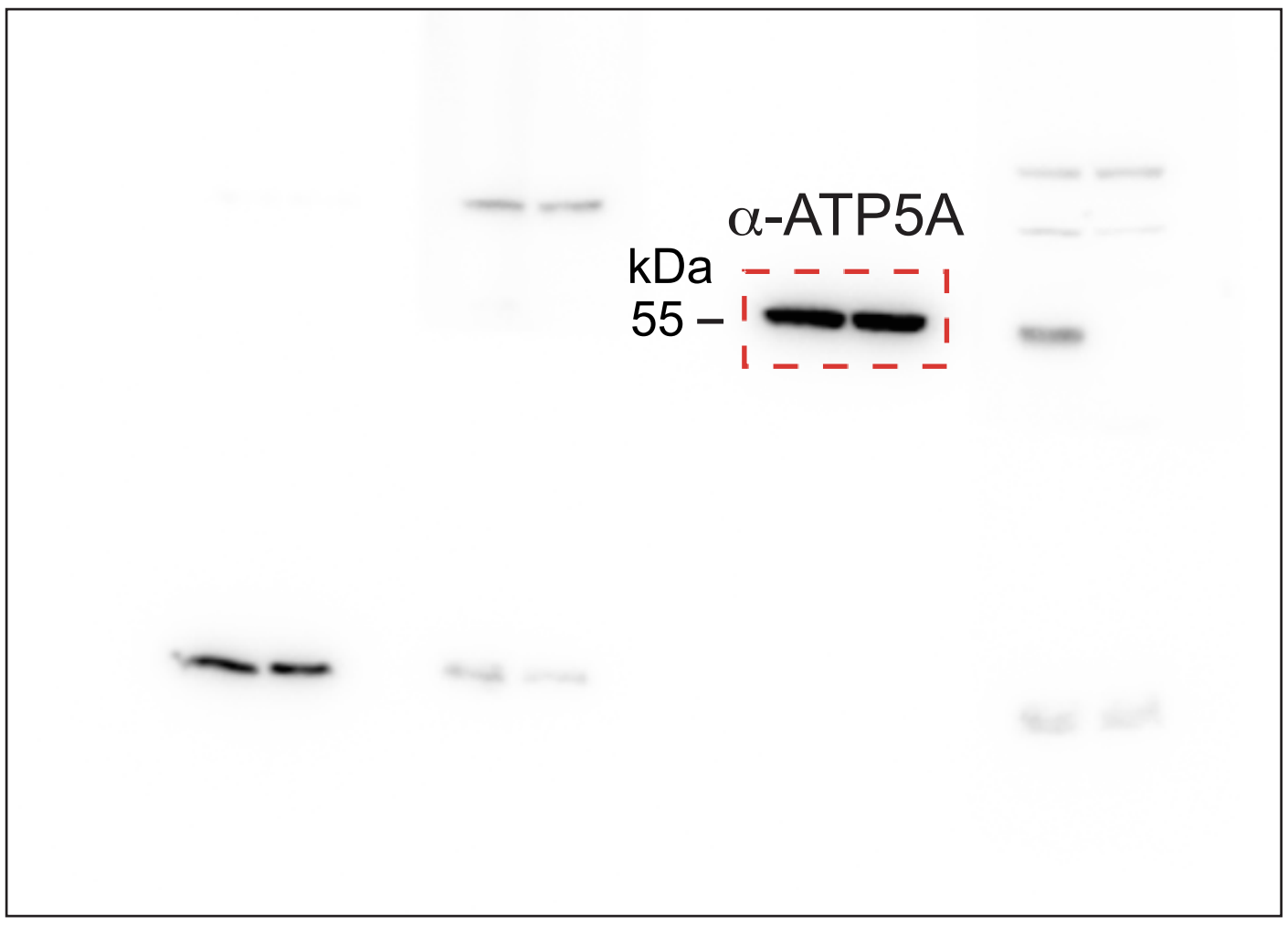

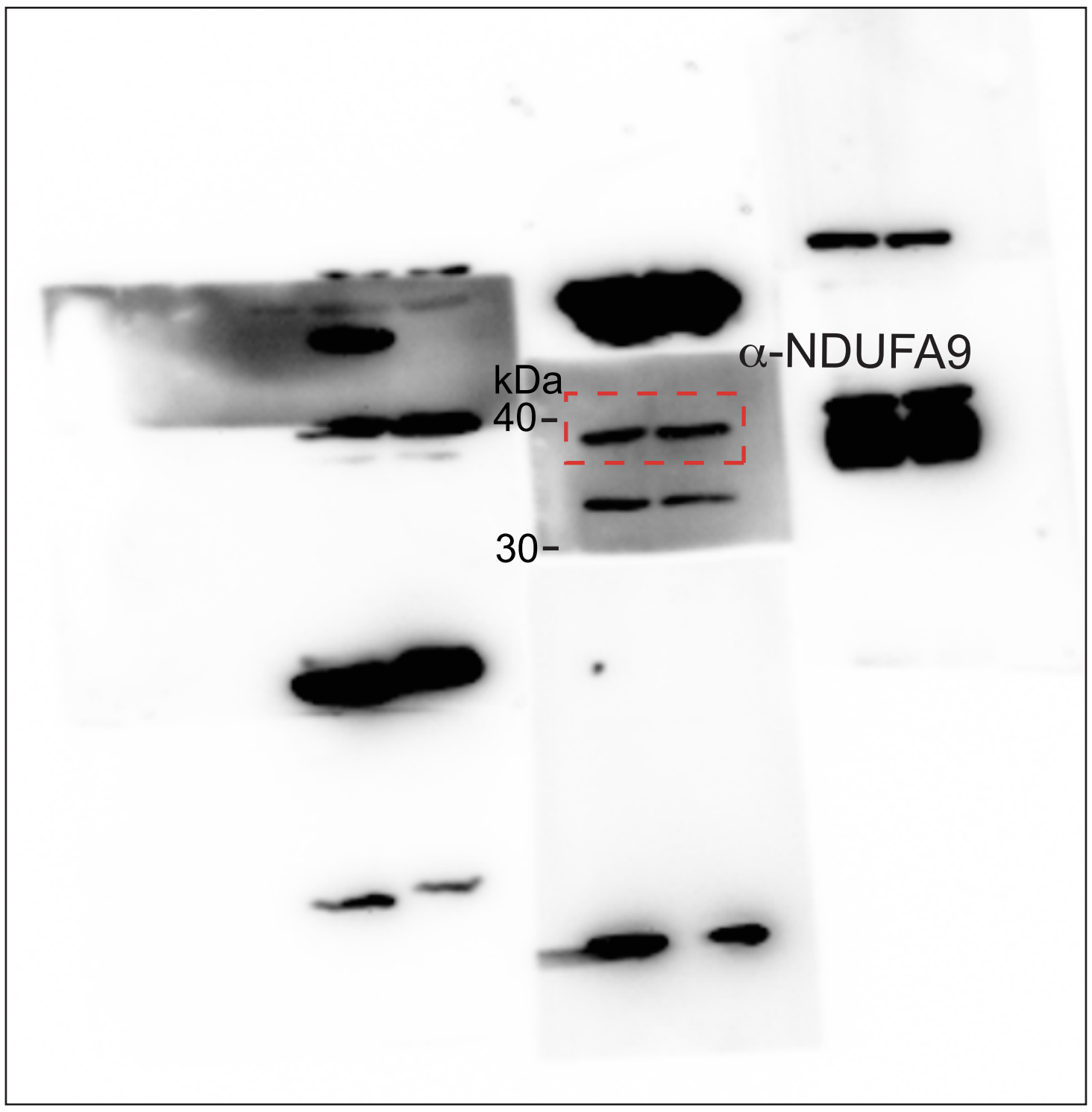

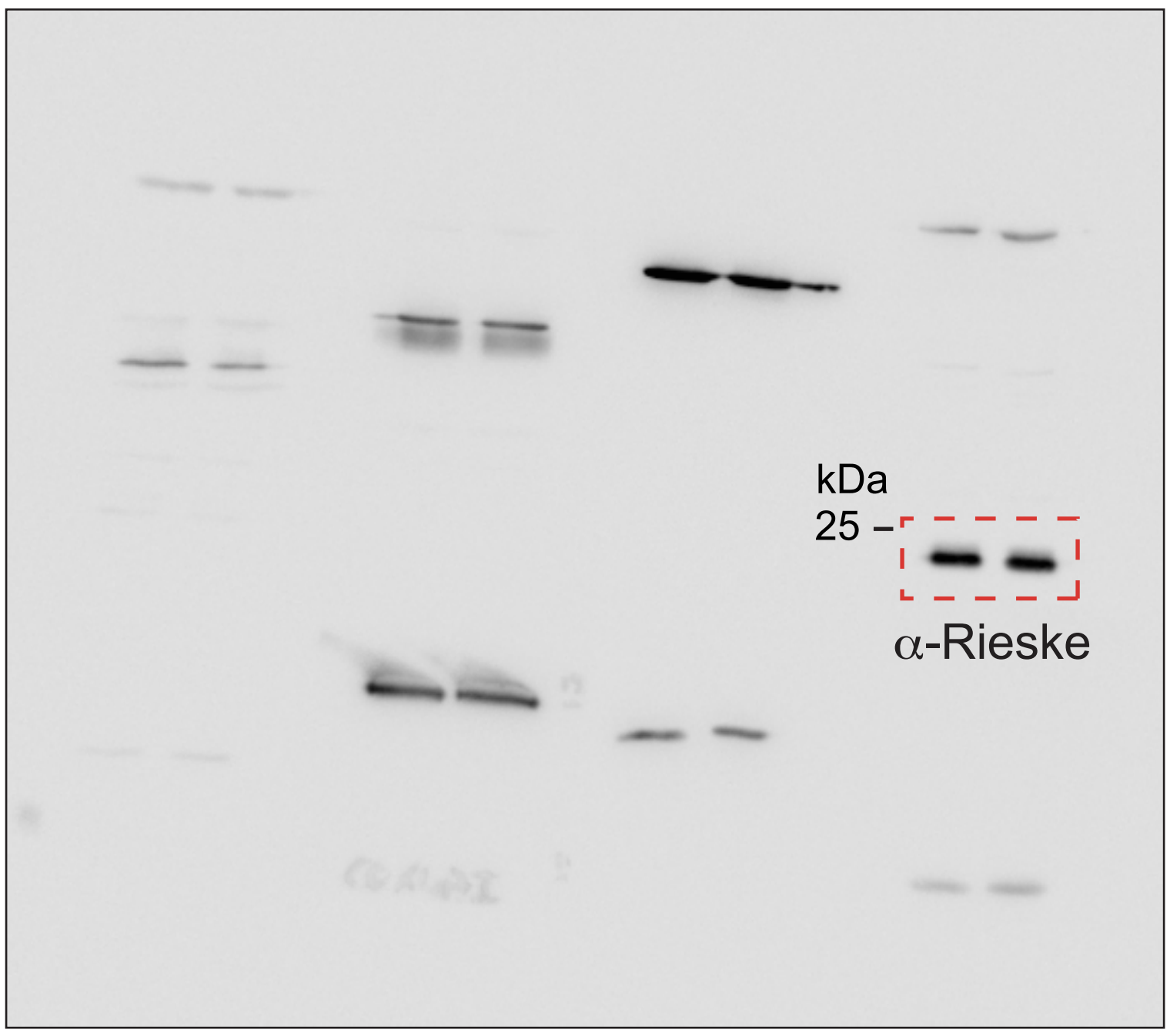

Extended Data Figure 4c

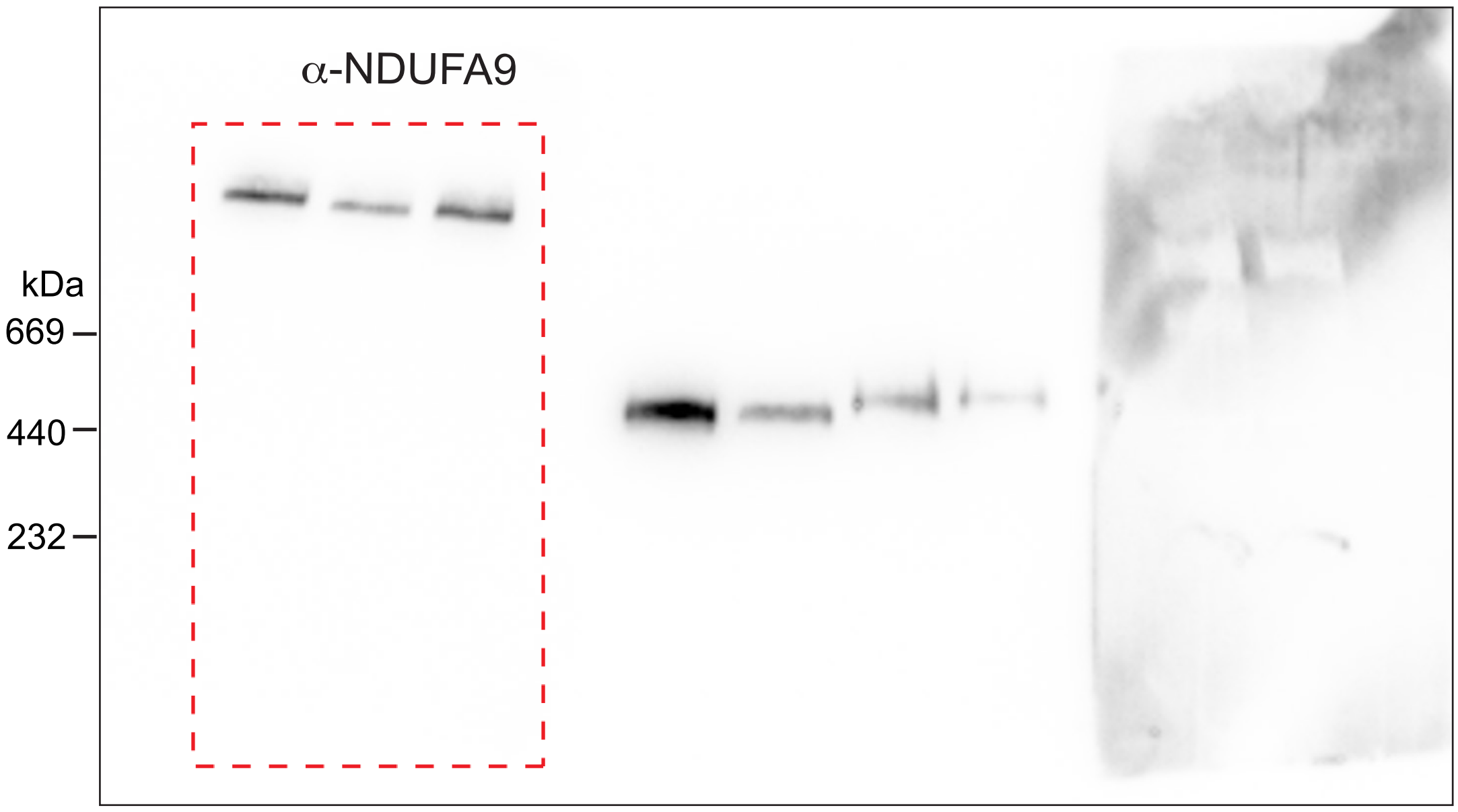

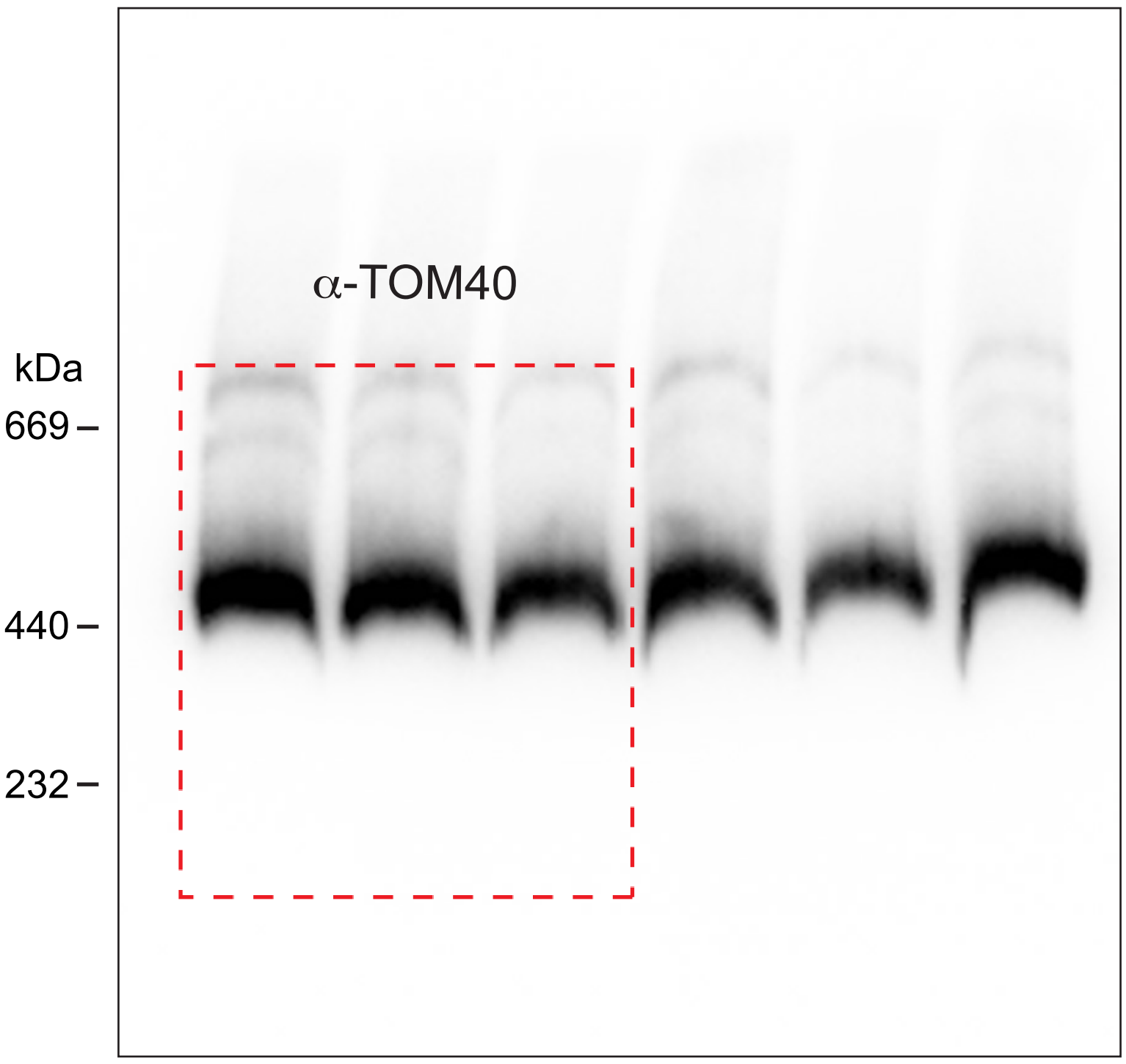

Extended Data Figure 5a

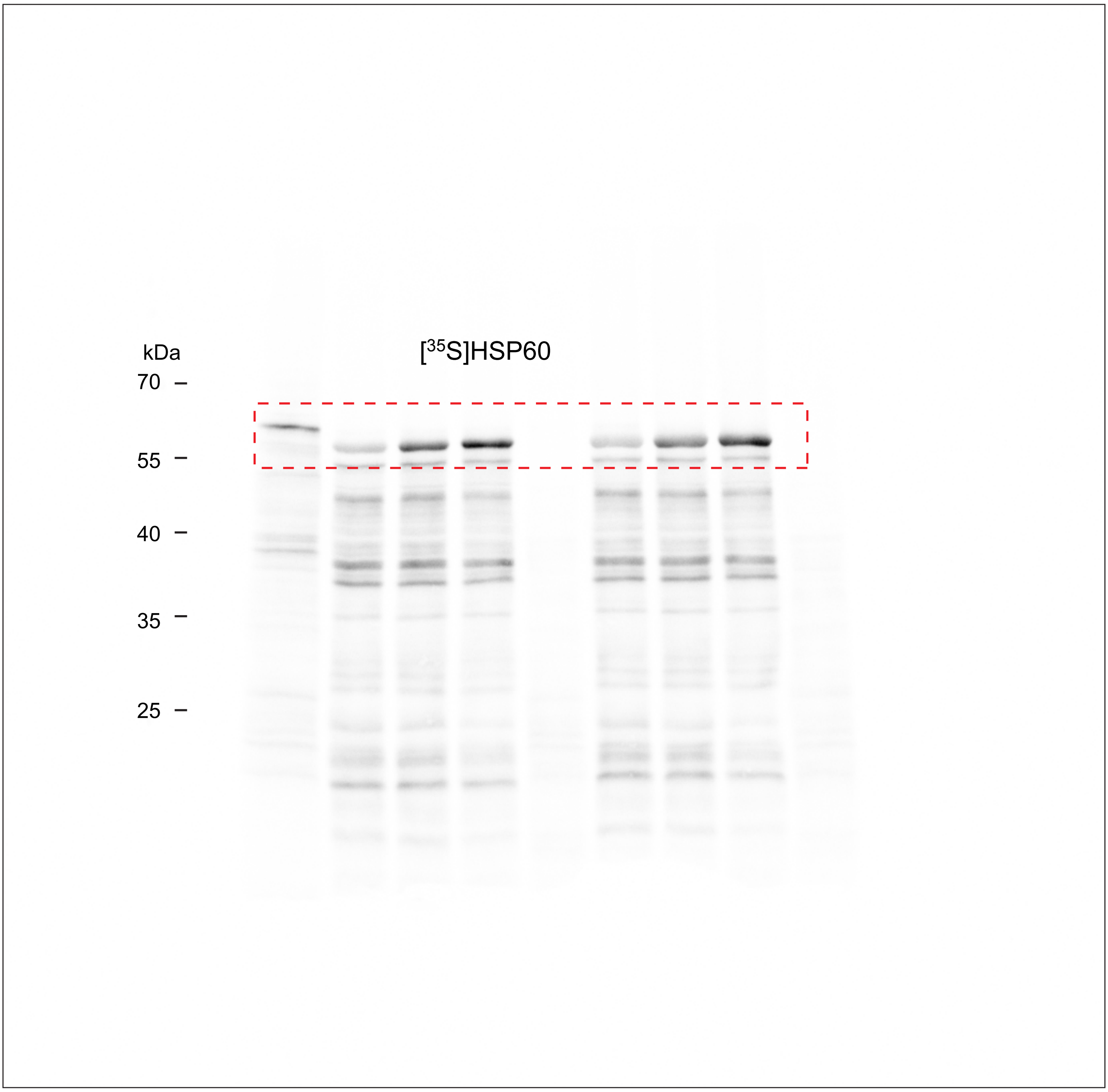

Extended Data Figure 10b

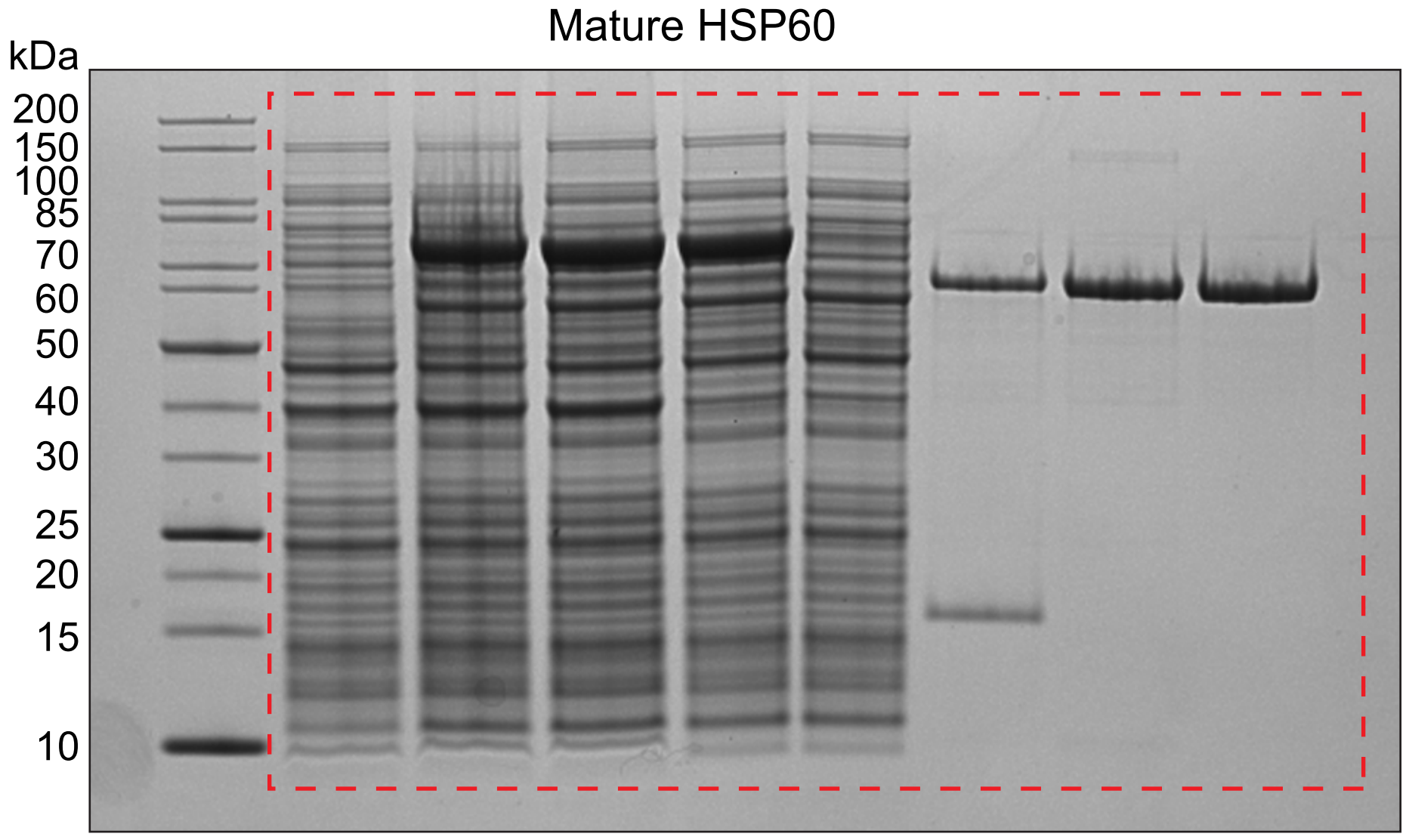

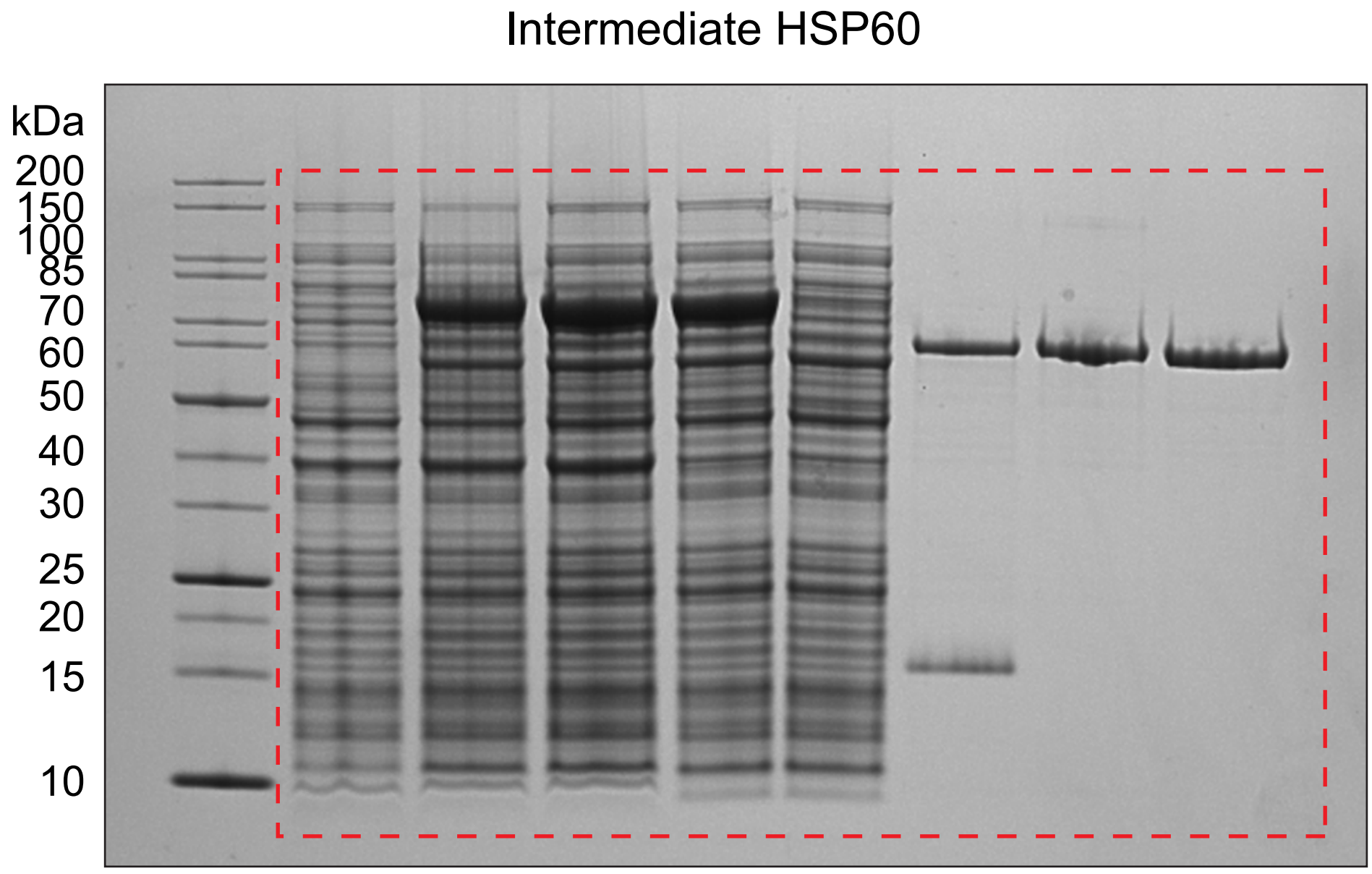

Extended Data Figure 10c

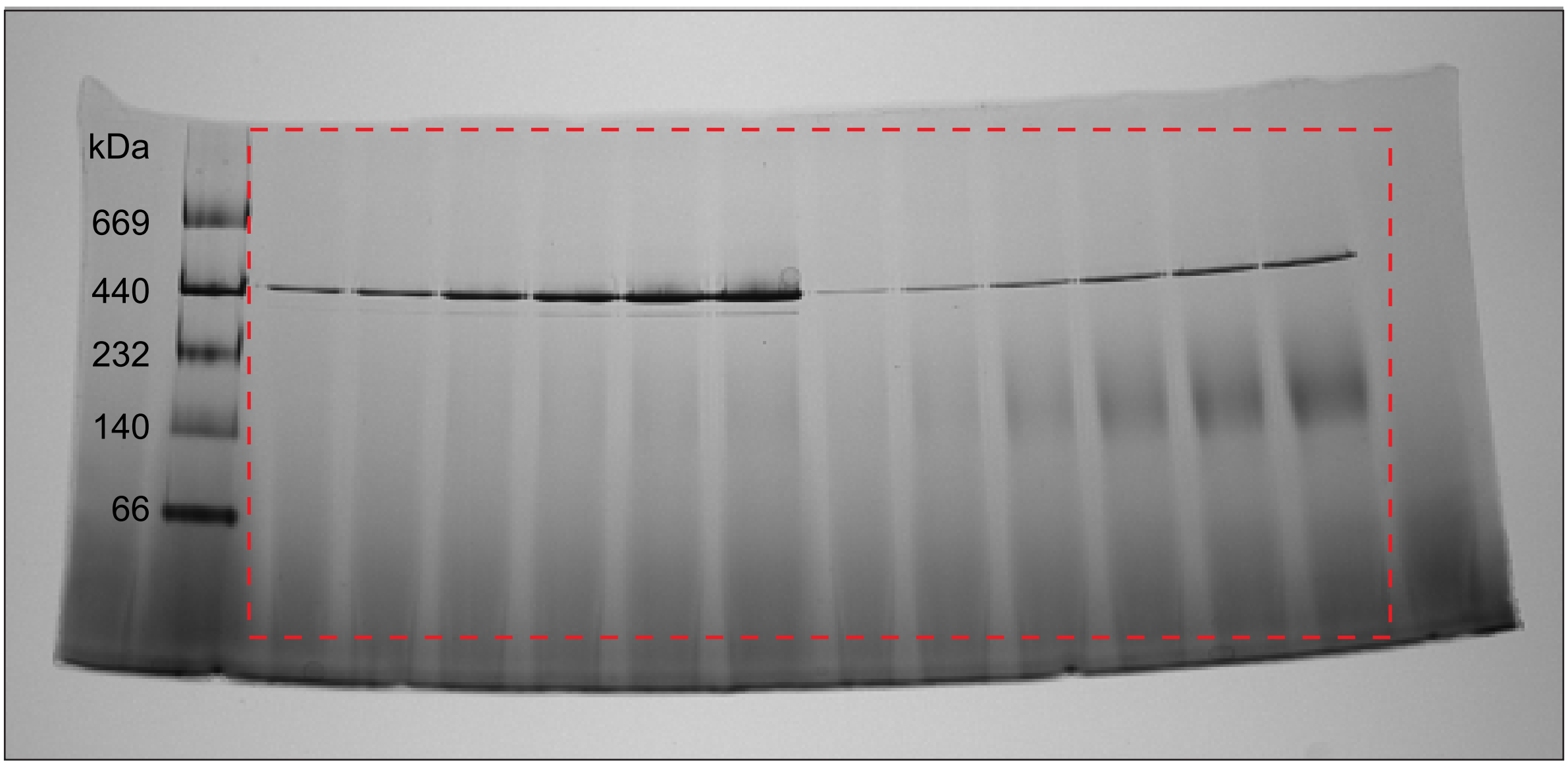

Supplement: Supplementary file 2 [file 41594_2026_1876_MOESM22_ESM.pdf]
