# Supplementary material for: A single-amino-acid cleavage controls global mitochondrial complex integrity
Source: Nat Struct Mol Biol. 2026 Aug 31;33(10):1535–47. doi: 10.1038/s41594-026-01876-7 (PMC13672106; doi:10.1038/s41594-026-01876-7)
Supplement: Supplementary file 3 [file 41594_2026_1876_MOESM2_ESM.pdf]

nature portfolio

Peer Review File

A single-amino-acid cleavage controls global mitochondrial complex integrity

Corresponding Author: Professor F-Nora Vögtle

Version 0:

Decision Letter:

30th Jan 2026

Dear Professor Vögtle,

Thank you again for submitting your manuscript "A single-amino-acid cleavage controls global mitochondrial complex integrity". I apologize for the delay in responding, which resulted from the difficulty in obtaining suitable referee reports. We now have comments (below) from the 3 reviewers who evaluated your paper. In light of those reports, we remain interested in your study and would like to see your response to the comments of the referees in the form of a revised manuscript.

You will see that while reviewers appreciate the results, they raise several concerns which will need to be addressed in a revision. Specifically, please revise the statistics and provide replicates and quantifications requested by reviewer #3 (points 3 and 4). Furthermore, we agree with reviewer #3 that further direct evidence to support that single N-term residue of substrates can affect protein complex integrity (point 1) will be essential to address. You will also see that reviewer #2 asks to elaborate on the potential mech of ICP55 regulating specific mitochondrial protein level in steady-state (points 2-3) and asks if ICP55 deficiency could have broader effects (pt 4) - the editorial team agrees that providing additional data to address and clarify these points would significantly strengthen the study. Please also, in line with comments of reviewers #1 and #3 clarify the number of substrates in the dataset, and provide a list. Please clearly discuss the caveats of the approaches and the experimental system, especially regarding the claims of ICP55 as global determinant of mitochondrial complex integrity (Reviewer #3 pt 2), if these cannot be strengthened experimentally.

Please be sure to address/respond to all concerns of the referees in full in a point-by-point response and highlight all changes in the revised manuscript text file.

Our standard revision period is 4 months. We appreciate that the requested revisions are extensive. We are committed to providing a fair and constructive peer-review process and encourage you to contact us if you would like to discuss the reviews or anticipate any delay with the resubmission.

We will be happy to consider your revision as long as nothing similar has been accepted for publication at NSMB or published elsewhere. Should your manuscript be substantially delayed without notifying us in advance and your article is eventually published, the received date would be that of the revised, not the original, version.

REPORTING AND DATA AVAILABILITY: Please provide updated files as follows with your revision. As you already know, we put great emphasis on ensuring that the methods and statistics reported in our papers are correct and accurate.

1. REPORTING SUMMARY: Please provide an updated file:

https://www.nature.com/documents/nr-reporting-summary.pdf

This form is a dynamic ‘smart pdf’ and must be downloaded and completed in Adobe Reader.

2. REPORTING OF STRUCTURAL DATA: If there are additional or modified structures in the revision, please submit the corresponding PDB validation reports with your other files, and use the figshare integration system to provide access to maps, half-maps, and models.

Manuscripts reporting new structures should contain a table summarizing structural and refinement statistics. Please include

these tables for new cryo-EM or -ET (https://www.nature.com/documents/nr-tables-cryo-em.doc and modifying this file for ET), NMR (https://www.nature.com/documents/nr-tables-nmr.doc) and X-ray crystallography (https://www.nature.com/documents/nr-tables-xray.doc). To facilitate assessment of the quality of the structural data, a stereo image of a portion of the electron density map (for crystallography papers) or of the superimposed lowest energy structures (>10; for NMR papers) should be provided with the submitted manuscript. If the reported structure represents a novel overall fold, a stereo image of the entire structure (as a backbone trace) should also be provided. For cryo-EM structures, a representative micrograph showing individual particles should be provided in the figures alongside a processing workflow (please see our Editorial for more information: https://www.nature.com/articles/s41594-025-01567-9).

DATA DEPOSITION: We require deposition of coordinates (and, in the case of crystal structures, structure factors) into the Protein Data Bank with the designation of immediate release upon publication (HPUB). Electron microscopy-derived density maps and coordinate data must be deposited in EMDB and released upon publication. Deposition and immediate release of NMR chemical shift assignments are requested. To avoid delays in publication, dataset accession numbers must be supplied with the revised manuscript and appropriate release dates must be indicated at the galley proof stage.

3. REPORTING OF LIGHT MICROSCOPY DATA: For any revision that includes light microscopy data, we ask our authors to please include a completed light microscopy reporting table [https://www.nature.com/documents/Light_microscopy_reporting_table.xlsx] to ensure the methods are described thoroughly. The table will be available to reviewers and ultimately published, should the manuscript be accepted at the journal. When submitting the revised version of your manuscript, please pay close attention to our href="https://www.nature.com/nature-portfolio/editorial-policies/image-integrity">Digital Image Integrity Guidelines.

4. CODE AND COMPUTATIONAL WORK: If newly developed, unpublished code was used in this work, it must be provided with the revision and access to it must be disclosed in the Code Availability Statement in the manuscript. Please also provide the completed software submission checklist. This form is a dynamic ‘smart pdf’ and must be downloaded and completed in Adobe Reader, instead of opening it in a web browser: https://www.nature.com/documents/nr-software-policy.pdf

If molecular dynamics (MD) simulations were performed, please refer to this Editorial and provide a completed MD simulations checklist with your revision: https://www.nature.com/articles/s42003-023-04653-0

Lastly, if it applies, please complete and upload the completed machine learning checklist with your submission for re-review: https://www.nature.com/documents/machine-learning-checklist.pdf

5. SOURCE GEL IMAGES: Unprocessed scans should be provided for all gels and western blots presented in figures and should be clearly labelled with the figure number in the file title. The source gel images should be uncropped, unmodified images, with molecular weight markers. Uncropped, unmodified gel images from existing experimental repeats, in addition to the data shown in the figures, can also be presented in source data. Please provide these files as PDF with your revision.

Please ensure that all control panels for gels and western blots are appropriately described as loading or IP controls. Lastly, please ensure that all images in the paper are checked for duplication of panels and for splicing of gel lanes.

6. SOURCE NUMERICAL DATA: we urge authors to provide, in tabular form in excel files, the data underlying the graphical representations used in figures. This is to further increase transparency in data reporting, as detailed in this editorial (http://www.nature.com/nsmb/journal/v22/n10/full/nsmb.3110.html). Please provide one excel file per figure max, with one panel per tab. When submitting files, the title field should indicate which figure the source data pertains to.

7. DATA AVAILABILITY: this journal strongly supports public availability of data. All data used in accepted papers should be available via a public data repository, or alternatively, as Supplementary Information. If data can only be shared on request, please explain why in your Data Availability Statement and in the correspondence with your editor.

For some data types, deposition in a public repository is mandatory as detailed below:

https://www.nature.com/nature-research/editorial-policies/reporting-standards#availability-of-data

Finally, please ensure that you retain unprocessed data and metadata files after publication, ideally archiving data in perpetuity, as these may be requested during the peer review and production process or after publication if any issues arise.

EXTENDED DATA FIGURES

When re-submitting your manuscript, please ensure that any supplementary figures and tables that are crucial to the manuscript’s conclusions are converted into Extended Data figures and tables to increase visibility of these data. Extended Data figures and tables are online-only (present in the online PDF and full-text HTML versions of the paper), peer-reviewed display items that provide essential background to the article but are not included in the main article due to space

constraints. A maximum of ten Extended Data display items (figures and tables) is permitted.

While we encourage the use of color in preparing figures, please note that this will incur a charge to partially defray the cost of printing. Information about color charges can be found at http://www.nature.com/nsmb/authors/submit/index.html#costs

Nature Structural & Molecular Biology is committed to improving transparency in authorship. As part of our efforts in this direction, we are now requesting that all authors identified as 'corresponding author' on published papers create and link their Open Researcher and Contributor Identifier (ORCID) with their account on the Manuscript Tracking System (MTS), prior to acceptance. This applies to primary research papers only. ORCID helps the scientific community achieve unambiguous attribution of all scholarly contributions. You can create and link your ORCID from the home page of the MTS by clicking on 'Modify my Springer Nature account'. For more information please visit please visit www.springernature.com/orcid.

Please use the link below to submit your revised manuscript and related files:

Link Redacted

Note: This URL links to your confidential home page and associated information about manuscripts you may have submitted, or that you are reviewing for us. If you wish to forward this email to co-authors, please delete the link to your homepage.

We look forward to seeing the revised manuscript and thank you for the opportunity to review your work.

Sincerely,

Yue Feng, PhD
(she/her)
Associate Editor
Nature Structural & Molecular Biology
ORCID: 0009-0002-9697-1903

Referee expertise:

Referee #1: mitochondrial dynamics

Referee #2: mitochondrial biology, proteomics

Referee #3: mitochondrial protein homeostasis

Reviewers' Comments:

Reviewer #1 (Remarks to the Author):

The N terminus of many newly synthesized proteins is further processed after cleavage of the presequence. This has been mainly investigated in yeast. In their present manuscript the authors set out to characterize this process in human cells by high resolution proteomics of cells lacking the ICP55 protease, which removes a single amino acid from the mature part of many imported proteins. Using this approach, they identified 107 ICP55 substrates in human mitochondria. They find that the assembly of subunits not processed by ICP55 into respiratory chain supercomplexes destabilizes these complexes and diminishes respiratory activity. Similarly, they found that the chaperonin HSP60 was destabilized in the absence of ICP55. Using a SILAC approach the authors demonstrate that mitochondrial high molecular weight complexes are generally less stable in cells lacking ICP55. These are novel and important observations. Some points should be addressed to improve the clarity of data presentation.

For clarity, please indicate on page 3 that Oct1 has been identified in yeast.

Size markers should be added to Fig. 1g and h.

The authors find that two proteins, SDHAF2 and COX7A2L, are stabilized in the absence of ICP55. Are these proteins substrates of ICP55? I assume that this information is somewhere hidden in the supplemental tables, but it would be easier if it would be mentioned in the text. Also, I understand that HSP60 is processed by ICP55. Is this correct? I was unable to find HSP60 in the supplemental tables (which I find very confusing). Is HSP10 also processed by ICP55? Please provide a simple table listing the 107 identified ICP55 substrates.

The list of Extended Data Tables on p. 61 of the manuscript is not in accordance with the excel files that were submitted. There is no Extended Data Table 2, Table 4 should be Table 3 etc. Please correct.

On p. 13 the authors mention that 304 proteins were identified as ICP55 substrates. Where does this number come from? I couldn't find it elsewhere in the text.

Reviewer #2 (Remarks to the Author):

This study represents a technically and conceptually strong advance in the understanding of mitochondrial protein maturation. The use of high-resolution, comparative N-terminomics to define ICP55 substrates in a proteome-wide and unbiased manner is particularly impressive and enables detection of single amino acid processing events that would be difficult to capture with conventional approaches. Combined with complexome profiling and functional analyses, the work provides valuable insights into how subtle N-terminal processing controls global mitochondrial complex integrity and proteostasis.

However, the current manuscript and dataset need to be improved for the publication in NSMB. Please find my comments below.

<Major>

1-1. Please clarify which dataset contains the final list of the 107 selected ICP55 substrate proteins. In addition, it would be helpful to indicate whether this list includes information on sub-mitochondrial localization and, for membrane proteins, their topology (e.g., orientation of the N-terminus relative to the matrix).

1-2 Are all 107 ICP55 substrate proteins localized to the mitochondrial matrix? If some substrates are annotated as inner membrane, intermembrane space (IMS), or outer mitochondrial membrane (OMM) proteins, please clarify how their N-termini are accessible to ICP55, which is presumed to act in the matrix.

2 The changes in protein abundance between ICP55 knockout and wild-type mitochondria presented in the Extended Data Tables appear complex, yet only a few examples (e.g., COX7A2L and HSP60) are discussed in the main text. Notably, several mitochondrial ribosomal protein (MRP) subunits show both increased and decreased abundance. Please clarify how ICP55 deficiency can differentially affect the abundance of distinct subunits within the same macromolecular complex.

3. Beyond macromolecular complex subunits, numerous individual mitochondrial proteins exhibit altered abundance upon ICP55 loss. Please elaborate on the possible mechanisms by which defective ICP55-mediated N-terminal processing could lead to changes in the steady-state levels of individual mitochondrial proteins.

4. What evidence supports the conclusion that ICP55 deficiency does not broadly affect the activity or function of other mitochondrial proteases or peptidases? Additional clarification or discussion of this point would strengthen the specificity of the conclusions.

<Minor>

In the current manuscript, it is difficult to directly connect the data presented in the Extended Data Tables with the figures and results discussed in the main text. Explicit citation of the relevant Extended Data Tables in the main text and figure legends would greatly improve clarity and facilitate further evaluation of the data and manuscript.

Reviewer #3 (Remarks to the Author):

The manuscript investigates the role of the mitochondrial intermediate cleaving peptidase ICP55 in human cells. Using HUNTER N-terminomics in HEK293T mitochondria, the authors map 446 processed mitochondrial N-termini and identify 107 high-confidence ICP55 substrates, extending known motifs beyond canonical Y/L/F to include R and Q at position 1. Quantitative proteomics shows relatively modest changes in steady-state protein abundance but increased levels of specific assembly factors (SDHAF2, COX7A2L). SILAC-based complexome profiling of mixed WT/ICP55-/- mitochondria indicates that many inner-membrane and matrix proteins shift from high-molecular-weight assemblies to smaller complexes or monomers, particularly in the ETC, mitoribosome and metabolic enzymes such as IDH3. The authors conclude that ICP55-mediated removal of a single N-terminal residue is a major determinant of mitochondrial complex integrity and suggest that defective N-terminal processing contributes to human mitochondrial disease and possibly age-associated decline. Overall, this is a very nice and interesting study, but several points need to be addressed.

Major points

1. The central mechanistic claim is that retention of a single N-terminal residue on ICP55 substrates directly destabilizes protein–protein interfaces and thereby impairs assembly and/or stability of complexes (ETC, HSP60, mitoribosome, IDH3). The data clearly support an association between ICP55 loss and decreased complex integrity, and HSP60 is a particularly convincing example. However, for most complexes the evidence remains correlative: steady-state levels of many ETC subunits are unchanged or only mildly altered, but supercomplex abundance is reduced, complexes are more urea sensitive, and OCR is decreased. These observations are fully consistent with impaired complex integrity but do not by themselves demonstrate that the one-residue extension on multiple subunits is the primary cause. There is no direct proof that incorporation of the unprocessed form is necessary and sufficient for destabilization. Rescue of ETC supercomplex stability and function by ICP55 re-expression, or targeted N-terminal mutants that mimic processed versus unprocessed

states in a defined subunit, would substantially strengthen the proposed causal link. In the absence of such data, the text should more carefully distinguish between “consistent with” and “demonstrates that” when attributing destabilization specifically to the extra residue.

2. The manuscript repeatedly describes ICP55 as a central determinant of global mitochondrial complex integrity and a key stabiliser of multimeric mitochondrial protein complexes. While the complexome data clearly show widespread effects in ICP55-/- mitochondria, only a subset of mitochondrial proteins (177/933) display significantly altered migration, and the magnitude of changes differs across complexes. A more accurate and defensible wording would emphasize a broad or widespread impact, particularly on matrix and inner-membrane assemblies, rather than implying near-universal dependence of all complexes on ICP55. Along the same lines, the estimate that around 300 of about 1100 mitochondrial proteins are ICP55 substrates is extrapolated from 107 experimentally validated substrates in HEK293T plus in silico predictions that are acknowledged to be incomplete. This is reasonable as an upper-bound estimate but should be clearly labeled as such, not phrased as a firm number. Likewise, the proposed contribution of ICP55 defects to human disease and ageing-related mitochondrial decline is interesting and plausible, but remains hypothetical at this stage. Therefore, the Discussion should consistently frame these as well-supported hypotheses that require validation in patient-derived cells or in vivo models, rather than as established mechanisms.

3. The “dis-elution score” approach based on Manhattan distances is reasonable in principle, but the statistical treatment is underdescribed and appears lenient. Significant alterations are called at $p \leq 0.05$ across 933 mitochondrial proteins, without an explicit correction for multiple testing. At this scale, a substantial number of false positives is expected by chance. For a study that builds key conclusions on a global shift in complex architecture, it is important either to apply a multiple-testing correction (for example an FDR procedure such as Benjamini–Hochberg) or to explicitly quantify the expected false discovery rate and discuss it. In addition, more quantitative summary metrics (for example changes in OSR distributions, or the fraction of total signal in high- versus low-molecular-weight bins in WT versus ICP55-/-) would more robustly support the claim of a proteome-wide bias toward smaller assemblies. When commenting on statistical approaches, one further point is that essentially all western blots in the manuscript need to be repeated with proper biological replicates and quantified. Single 1+1 blots without quantification are not acceptable as evidence for abundance differences.

4. The BN-PAGEs in Figure 2 are not of sufficient quality. The supercomplex region appears collapsed into essentially one broad band, suggesting that the gels have not been run long enough to resolve respiratory supercomplexes adequately. These experiments should be repeated under conditions that provide proper separation. In addition, the quantification of BN-PAGEs in Figures 2d and 2g is problematic: control values are all normalised to 1 and thus show no variation, which is not an appropriate way to represent statistics across biological replicates.

Version 1:

Decision Letter:

Our ref: NSMB-A52112A

19th Jun 2026

Dear Professor Vögtle,

Thank you for submitting your revised manuscript "A single-amino-acid cleavage controls global mitochondrial complex integrity" (NSMB-A52112A). It has now been seen by the original referees and their comments are below. I apologise for the delay in issuing our editorial decision which came to be due to difficulty in obtaining all reviewers' reports. Nevertheless, we now have all feedback, with the reviewers finding that the paper has improved in revision. In that light we can accept the manuscript in principle for publication in Nature Structural & Molecular Biology, pending minor revisions to satisfy the referees' final requests and to fully comply with our editorial and formatting guidelines.

We are now performing detailed checks on your paper and will send you a checklist detailing our editorial and formatting requirements in a couple of weeks. Please do not upload the final materials and make any revisions until you receive this additional information from us.

To facilitate our work at this stage, it is important that we have a copy of the main text as a word file. If you could please send along a word version of this file as soon as possible, we would greatly appreciate it; please make sure to copy the NSMB account (cc'ed above).

Thank you again for your interest in Nature Structural & Molecular Biology Please do not hesitate to contact me if you have any questions.

Sincerely,

Dimitris Typas
Senior Editor
Nature Structural & Molecular Biology
ORCID: 0000-0002-8737-1319

Reviewer #1 (Remarks to the Author):

The authors have responded to my previous concerns in an adequate manner. Publication of this interesting manuscript can now be recommended.

Reviewer #2 (Remarks to the Author):

I have carefully evaluated the revised manuscript and the authors' point-by-point response. The authors have addressed my previous concerns and the manuscript has been substantially improved. Specifically, the addition of the new protease activity assays (evaluating MPP, MIP, IMP, and PreP) convincingly demonstrates that the observed complexome destabilization is specifically due to ICP55 deficiency, rather than a broad, non-specific impairment of mitochondrial proteases. Furthermore, the reorganization of Extended Data Table 3 to include submitochondrial localization annotations greatly improves the clarity and accessibility of the 107-substrate dataset.

Given the technical rigor and the conceptual advance presented in this revised version, I recommend the acceptance of this manuscript for publication in Nature Structural & Molecular Biology.

Reviewer #3 (Remarks to the Author):

I thank the authors for their careful and satisfactory revision. The manuscript has been substantially strengthened, and my main concerns have been appropriately addressed.
Most importantly, the new ICP55 re-expression experiment provides a key causal control. The rescue of respiratory chain supercomplex abundance and oxygen consumption strongly supports the conclusion that the observed mitochondrial defects are specifically caused by loss of ICP55. This addition considerably improves the mechanistic strength of the study.
Overall, the authors have addressed my concerns in a detailed and convincing manner.

INTERNAL USE ONLY AIP modules required: ["Life Science"]

Version 2:

Decision Letter:

3rd Aug 2026

Dear Professor Vögtle,

We are now happy to accept your revised paper "A single-amino-acid cleavage controls global mitochondrial complex integrity" for publication as an Article in Nature Structural & Molecular Biology.

Acceptance is conditional on the manuscript's not being published elsewhere and on there being no announcement of this work to the newspapers, magazines, radio or television until the publication date in Nature Structural & Molecular Biology.

Over the next few weeks, your paper will be copyedited to ensure that it conforms to Nature Structural & Molecular Biology style. Once your paper is typeset, you will receive an email with a link to choose the appropriate publishing options for your paper and our Author Services team will be in touch regarding any additional information that may be required.

After the grant of rights is completed, you will receive a link to your electronic proof via email with a request to make any corrections within 48 hours. If, when you receive your proof, you cannot meet this deadline, please inform us at rjsproduction@springernature.com immediately.

You will not receive your proofs until the publishing agreement has been received through our system.

Due to the importance of these deadlines, we ask that you please let us know now whether you will be difficult to contact over the next month. If this is the case, we ask you provide us with the contact information (email, phone and fax) of someone who will be able to check the proofs on your behalf, and who will be available to address any last-minute problems.

To assist our authors in disseminating their research to the broader community, our SharedIt initiative provides all co-authors with the ability to generate a unique shareable link that will allow anyone (with or without a subscription) to read the published article. Recipients of the link with a subscription will also be able to download and print the PDF.

As soon as your article is published, you can generate your shareable link by entering the DOI of your article here: <a

href="http://authors.springernature.com/share">http://authors.springernature.com/share. Corresponding authors will also receive an automated email with the shareable link

Note the policy of the journal on data deposition: http://www.nature.com/authors/policies/availability.html.

Your paper will be published online soon after we receive proof corrections and will appear in print in the next available issue. You can find out your date of online publication by contacting the production team shortly after sending your proof corrections.

You may wish to make your media relations office aware of your accepted publication, in case they consider it appropriate to organize some internal or external publicity. Once your paper has been scheduled you will receive an email confirming the publication details. This is normally 3-4 working days in advance of publication. If you need additional notice of the date and time of publication, please let the production team know when you receive the proof of your article to ensure there is sufficient time to coordinate. Further information on our embargo policies can be found here: https://www.nature.com/authors/policies/embargo.html

You can now use a single sign-on for all your accounts, view the status of all your manuscript submissions and reviews, access usage statistics for your published articles and download a record of your refereeing activity for the Nature journals.

If you have not already done so, we strongly recommend that you upload the step-by-step protocols used in this manuscript to the Protocol Exchange. Protocol Exchange is an open online resource that allows researchers to share their detailed experimental know-how. All uploaded protocols are made freely available, assigned DOIs for ease of citation and fully searchable through nature.com. Protocols can be linked to any publications in which they are used and will be linked to from your article. You can also establish a dedicated page to collect all your lab Protocols. By uploading your Protocols to Protocol Exchange, you are enabling researchers to more readily reproduce or adapt the methodology you use, as well as increasing the visibility of your protocols and papers. Upload your Protocols at www.nature.com/protocolexchange/. Further information can be found at www.nature.com/protocolexchange/about.

An online order form for reprints of your paper is available at https://www.nature.com/reprints/author-reprints.html. Please let your coauthors and your institutions' public affairs office know that they are also welcome to order reprints by this method.

Authors may need to take specific actions to achieve compliance with funder and institutional open access mandates. If your research is supported by a funder that requires immediate open access (e.g. according to Plan S principles or the NIH public access policy) then you should select the gold OA route, and we will direct you to the compliant route where possible. Because authors warrant under our subscription licensing terms that they haven't committed to licensing any version of their article under a licence inconsistent with the terms of our agreement – including the applicable embargo period – publication under the subscription model isn't suitable for authors whose funders require no embargo.

In approximately 10 business days you will receive an email with a link to choose the appropriate publishing options for your paper and our Author Services team will be in touch regarding any additional information that may be required.

You will not receive your proofs until the publishing agreement has been received through our system.

If you have any questions about our publishing options, costs, Open Access requirements, or our legal forms, please contact ASJournals@springernature.com

Sincerely,

Dimitris Typas
Senior Editor
Nature Structural & Molecular Biology
ORCID: 0000-0002-8737-1319

Click here if you would like to recommend Nature Structural & Molecular Biology to your librarian: http://www.nature.com/subscriptions/recommend.html#forms

Open Access This Peer Review File is licensed under a Creative Commons Attribution 4.0 International License, which permits use, sharing, adaptation, distribution and reproduction in any medium or format, as long as you give appropriate credit to the original author(s) and the source, provide a link to the Creative Commons license, and indicate if changes were made.
In cases where reviewers are anonymous, credit should be given to 'Anonymous Referee' and the source.
The images or other third party material in this Peer Review File are included in the article's Creative Commons license, unless indicated otherwise in a credit line to the material. If material is not included in the article's Creative Commons license and your intended use is not permitted by statutory regulation or exceeds the permitted use, you will need to obtain permission directly from the copyright holder.
To view a copy of this license, visit https://creativecommons.org/licenses/by/4.0/

Point-by-point response

Reviewer #1:
The N terminus of many newly synthesized proteins is further processed after cleavage of the presequence. This has been mainly investigated in yeast. In their present manuscript the authors set out to characterize this process in human cells by high resolution proteomics of cells lacking the ICP55 protease, which removes a single amino acid from the mature part of many imported proteins. Using this approach, they identified 107 ICP55 substrates in human mitochondria. They find that the assembly of subunits not processed by ICP55 into respiratory chain supercomplexes destabilizes these complexes and diminishes respiratory activity. Similarly, they found that the chaperonin HSP60 was destabilized in the absence of ICP55. Using a SILAC approach the authors demonstrate that mitochondrial high molecular weight complexes are generally less stable in cells lacking ICP55. These are novel and important observations. Some points should be addressed to improve the clarity of data presentation.

We thank the reviewer for the positive assessment of our manuscript.

For clarity, please indicate on page 3 that Oct1 has been identified in yeast.

We thank the reviewer for pointing this out. We have clarified in the revised manuscript that the mitochondrial intermediate peptidase (MIP/Oct1) was originally identified in both rat and yeast, and we now cite the corresponding primary studies. We also explicitly state that its functional characterization has been carried out predominantly in the yeast model system.

Size markers should be added to Fig. 1g and h.

We have added molecular weight markers to all SDS-PAGE based analyses in the revised version.

The authors find that two proteins, SDHAF2 and COX7A2L, are stabilized in the absence of ICP55. Are these proteins substrates of ICP55? I assume that this information is somewhere hidden in the supplemental tables, but it would be easier if it would be mentioned in the text. Also, I understand that HSP60 is processed by ICP55. Is this correct? I was unable to find HSP60 in the supplemental tables (which I find very confusing). Is HSP10 also processed by ICP55? Please provide a simple table listing the 107 identified ICP55 substrates.

SDHAF2 and COX7A2L were not identified as ICP55 substrates in our HUNTER analysis. However, SDHAF2 was predicted to be an ICP55 substrate by TargetP2, albeit with moderate probability (0.45). To experimentally assess potential ICP55-dependent processing, we synthesized radiolabeled precursor proteins of SDHAF2 and COX7A2L and performed *in organello* import assays into isolated wild-type (WT) and $ICP55^{KO}$ mitochondria (**new Fig. R1a,b**). COX7A2L was imported in a membrane potential-dependent manner but did not undergo processing upon import. SDHAF2 was likewise imported in a membrane potential-dependent manner and underwent presequence processing. However, no difference in migration between WT and $ICP55^{KO}$ mitochondria was detected. Consistently, SDHAF2 migration was indistinguishable between WT and $ICP55^{KO}$ mitochondria by SDS-PAGE and immunoblotting (**new Fig. R1c**). We have revised the manuscript text to explicitly state that neither SDHAF2 nor COX7A2L were identified as ICP55 substrates in our N-terminome.
By contrast, HSP60 was identified as an ICP55 substrate in our N-terminome. Due to its size (appr. 60 kDa), a single amino acid difference is not resolvable by SDS-PAGE and immunoblotting. To validate ICP55-dependent cleavage, we generated a C-terminally truncated radiolabeled HSP60 precursor ($HSP60^{1-228}$) and performed *in organello* import assays. The processed HSP60 displayed a migration difference between WT and $ICP55^{KO}$ mitochondria, consistent with the presence or absence of ICP55 mediated single residue trimming (**new Fig. R1d**).

HSP10 is not processed upon import and is therefore not an ICP55 substrate, as confirmed by immunoblot analysis of WT and ICP55^{KO} mitochondria (**new Fig. R1c**).
To improve clarity and accessibility, we have reformatted the Extended Data Tables to ensure that all relevant information is clearly presented and easily searchable. In particular, Extended Data Table 3 has been updated to provide a comprehensive and user-friendly overview of the experimentally determined ICP55 substrates.

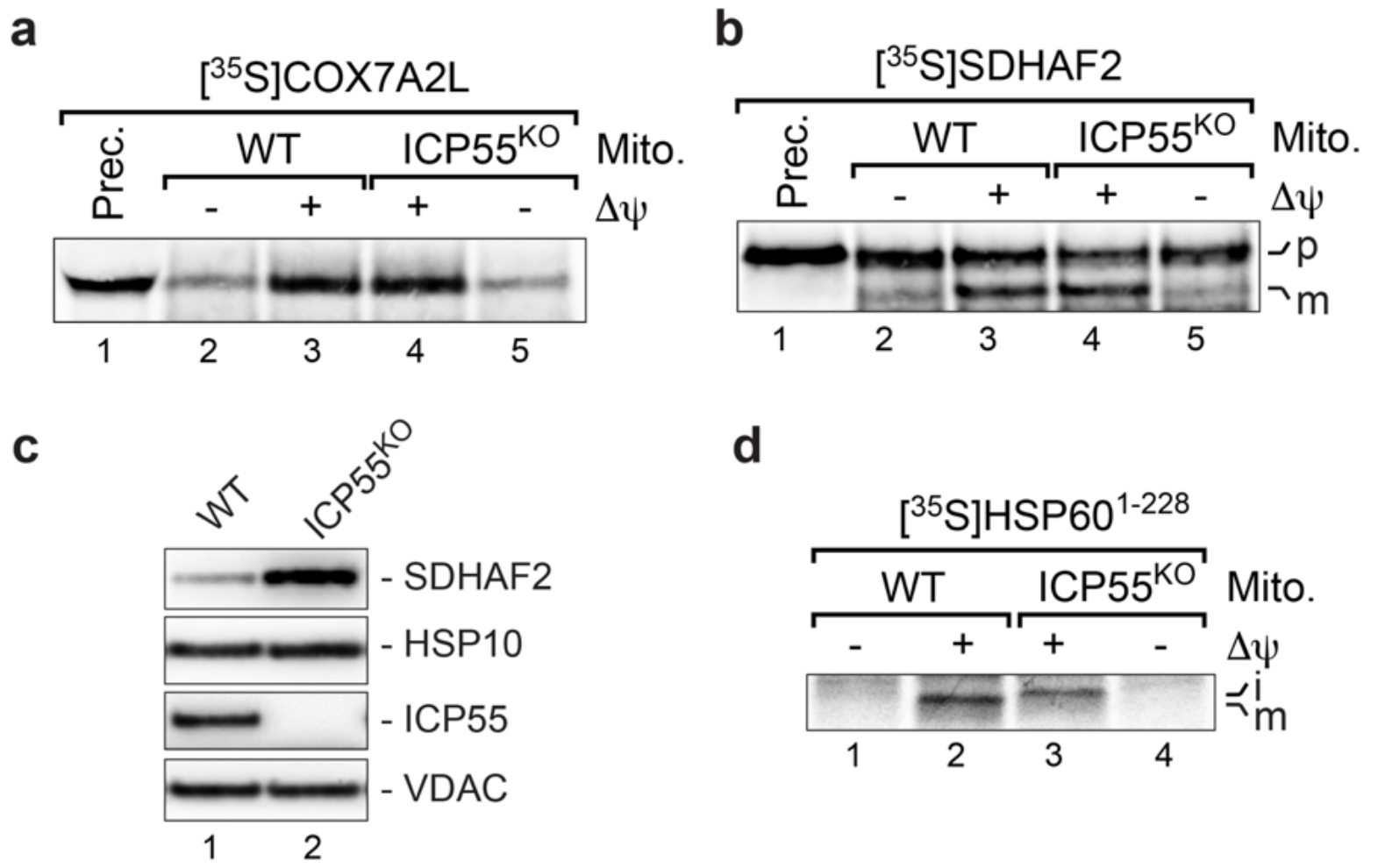

New Figure R1. a,b) *In organello* import of radiolabeled precursor proteins into isolated wild-type (WT) and ICP55^{KO} mitochondria. Where indicated the membrane potential (Δψ) was disspiated prior to the import reaction. p, precursor; m, mature protein. c) Analysis of protein levels in isolated mitochondria by SDS-PAGE and immunoblotting. d) Import of C-terminally truncated radiolabeled HSP60 (HSP60$^{1-228}$) performed as in a).

The list of Extended Data Tables on p. 61 of the manuscript is not in accordance with the excel files that were submitted. There is no Extended Data Table 2, Table 4 should be Table 3 etc. Please correct.

We apologize for the issues with the tables. We have carefully reviewed and corrected the tables throughout the manuscript and within the excel files to ensure consistency.

On p. 13 the authors mention that 304 proteins were identified as ICP55 substrates. Where does this number come from? I couldn't find it elsewhere in the text.

The number refers to the combined total of ICP55 substrates identified experimentally and predicted by MitoFates and TargetP. We have clarified this in the revised manuscript.

Reviewer #2:
This study represents a technically and conceptually strong advance in the understanding of mitochondrial protein maturation. The use of high-resolution, comparative N-terminomics to define ICP55 substrates in a proteome-wide and unbiased manner is particularly impressive and enables detection of single amino acid processing events that would be difficult to capture with conventional approaches. Combined with complexome profiling and functional analyses, the work provides valuable insights into how subtle N-terminal processing controls global mitochondrial complex integrity and proteostasis.

However, the current manuscript and dataset need to be improved for the publication in NSMB. Please find my comments below.

We thank the reviewer for the positive feedback on our manuscript.

<Major>
1-1. Please clarify which dataset contains the final list of the 107 selected ICP55 substrate proteins. In addition, it would be helpful to indicate whether this list includes information on sub-mitochondrial localization and, for membrane proteins, their topology (e.g., orientation of the N-terminus relative to the matrix).

The final, experimentally determined ICP55 substrates are listed in Extended Data Table 3. We have reformatted this table to improve accessibility and clarity, and it now includes mitochondrial sublocalization (annotated based on MitoCarta 3.0). We have also incorporated this information into the Results section of the revised manuscript. All experimentally identified ICP55 substrates localize to the mitochondrial matrix or inner membrane, consistent with their accessibility to MPP and ICP55 processing. Information on the topology of mitochondrial proteins is to our knowledge currently not available in a high-throughput format and was not systematically included (see also response below).

1-2 Are all 107 ICP55 substrate proteins localized to the mitochondrial matrix? If some substrates are annotated as inner membrane, intermembrane space (IMS), or outer mitochondrial membrane (OMM) proteins, please clarify how their N-termini are accessible to ICP55, which is presumed to act in the matrix.

We have added the mitochondrial sublocalization annotations based on MitoCarta 3.0 to the Extended Data Table 3. Importantly, all experimentally identified ICP55 substrates localize to the matrix or inner membrane. Information on inner membrane protein topology is not yet available in a high-throughput format and was therefore not included. However, as ICP55-mediated processing follows MPP cleavage, which occurs in the matrix, this strongly suggests that the N-termini of these proteins are exposed to the matrix and thus accessible to both proteases.

2 The changes in protein abundance between ICP55 knockout and wild-type mitochondria presented in the Extended Data Tables appear complex, yet only a few examples (e.g., COX7A2L and HSP60) are discussed in the main text. Notably, several mitochondrial ribosomal protein (MRP) subunits show both increased and decreased abundance. Please clarify how ICP55 deficiency can differentially affect the abundance of distinct subunits within the same macromolecular complex.

We have annotated all significantly changed proteins (increased and decreased) in the volcano plot (**new Fig. R2a**). Four proteins were significantly decreased (ICP55 itself, ACADVL (an ICP55 substrate), PUSL1, and ACSS1), which are functionally diverse and do not belong to a common pathway or complex. In contrast, four proteins were significantly increased (SDHAF2, COX7A2L, GLDC, and SDHAF4), three of which (SDHAF2, COX7A2L, and SDHAF4) are respiratory chain assembly factors. This functional enrichment among the increased proteins motivated our focus on respiratory chain integrity in subsequent analyses. (We did not further emphasize SDHAF4 in the main text, as a suitable antibody was not available to independently validate its increase via immunoblotting.)
Regarding the mitochondrial ribosome, our quantitative proteomic analysis shows that most subunits are either unchanged or slightly decreased (**Extended Data Fig. 8e**), indicating a consistent trend rather than opposing regulation within the complex. In cases where individual subunits of a complex may show variable behavior, this could reflect differential turnover of submodules rather than coordinated regulation of the fully assembled complex. For example, it has been shown that specific modules of respiratory complex I, such as the N-module, exhibit higher turnover rates than the remainder of the complex (Szczepanowska et al., 2020).

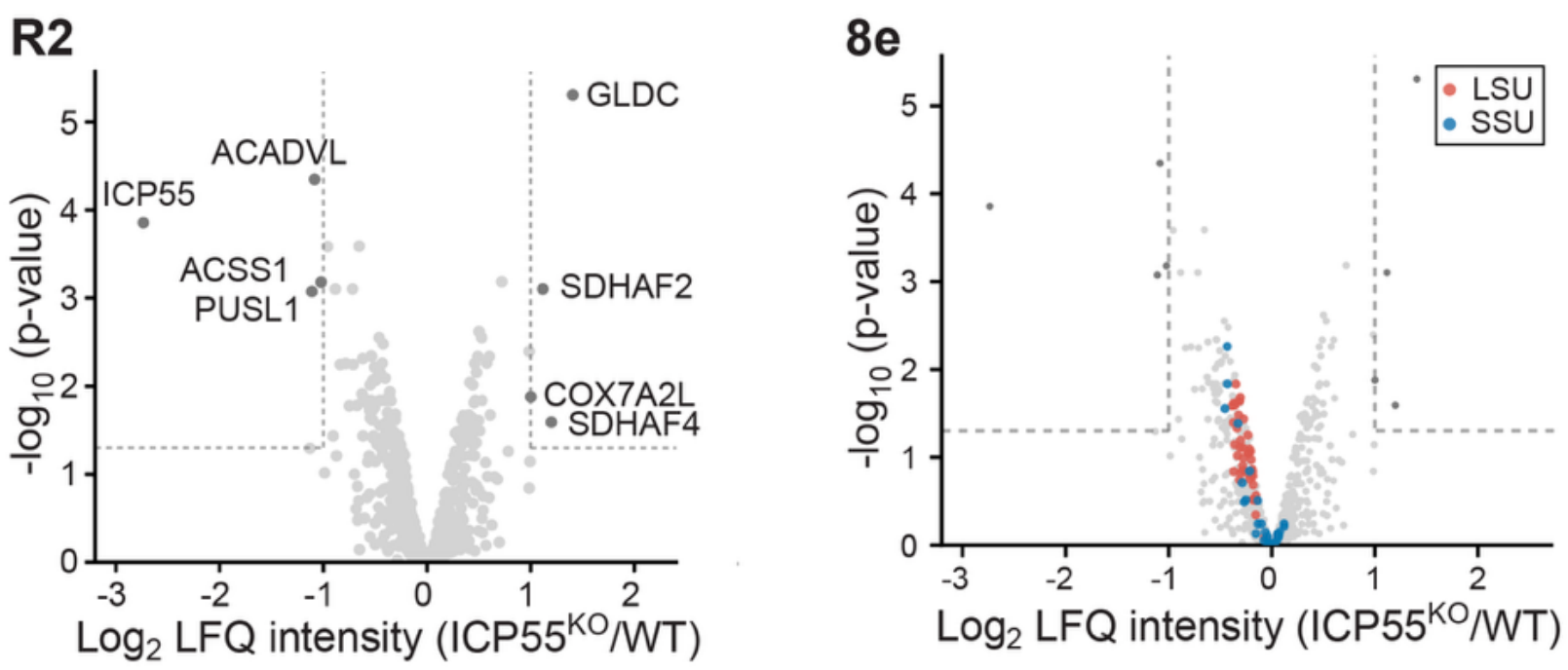

New Figure R2 and Extended Data Figure 8e. Volcano plot visualizing mitochondrial protein abundance in wild-type (WT) and ICP55^{KO} cells. n=5 biological replicates. Significantly changed proteins are labeled in R2 and the subunits of the mitochondrial ribosome are labeled in color in 8e.

3. Beyond macromolecular complex subunits, numerous individual mitochondrial proteins exhibit altered abundance upon ICP55 loss. Please elaborate on the possible mechanisms by which defective ICP55-mediated N-terminal processing could lead to changes in the steady-state levels of individual mitochondrial proteins.

We thank the reviewer for this important point. Incomplete ICP55 processing results in the accumulation of processing intermediates with N-termini starting with Y, L, F, R or Q, which are relatively bulky amino acids and may also affect folding of individual, monomeric proteins. Furthermore, in previous work in yeast, Icp55 substrates with the N-terminal residues Y, L, or F displayed reduced half-lives and increased turnover, consistent with principles of the bacterial N-end rule (Vögtle et al., 2009). These findings suggested that Icp55-mediated processing can influence protein degradation by removing destabilizing N-terminal residues.
Based on this, one possible explanation is that, in the absence of ICP55, monomeric mitochondrial proteins may undergo increased degradation due to the exposure of such N-terminal residues (N-degrons), which are recognized by mitochondrial proteases. However, the existence of a defined N-end rule pathway in mitochondria and the identity of the responsible proteases remain to be established.
In our human dataset, we observe that loss of ICP55 is associated with compromised complex stability. We therefore propose a model in which impaired ICP55 processing affects the stability of protein complexes, leading to their partial disassembly. In this scenario, unassembled subunits that retain an additional N-terminal residue in the absence of ICP55 may be preferentially recognized and degraded, potentially via an N-end rule-like mechanism. This model would provide a possible explanation for the observed changes in steady state protein levels.
We have incorporated this discussion into the revised manuscript to provide a mechanistic model while acknowledging that further work is required to define the underlying pathways.

4. What evidence supports the conclusion that ICP55 deficiency does not broadly affect the activity or function of other mitochondrial proteases or peptidases? Additional clarification or discussion of this point would strengthen the specificity of the conclusions.

We have experimentally assessed whether loss of ICP55 affects the activity of other mitochondrial proteases and peptidases. Specifically, we analyzed the activity of the presequence processing proteases MPP, MIP and IMP, as well as the major mitochondrial peptidase PreP, in wild-type and ICP55^{KO} mitochondria.
To evaluate presequence protease activity, we performed *in organello* import assays using established substrate proteins. We did not observe differences in processing efficiency for the MPP substrate frataxin, the MPP/MIP substrate OTC, or the IMP substrate DIABLO between wild-type and ICP55^{KO} mitochondria (**new Fig. R3a-c**).
In addition, we assessed PreP activity using soluble mitochondrial extracts and a fluorogenic peptide substrate (Mca-RPPGFSAFK(Dnp)-OH), whose degradation is monitored by increased fluorescence (Kücükköse et al., 2024). Degradation kinetics were indistinguishable between wild-type and ICP55^{KO} extracts, indicating unchanged PreP activity (**new Fig. R3d**).

Together, these results demonstrate that loss of ICP55 does not broadly impair the activity of other mitochondrial presequence proteases or peptidases, supporting the specificity of the observed phenotypes.

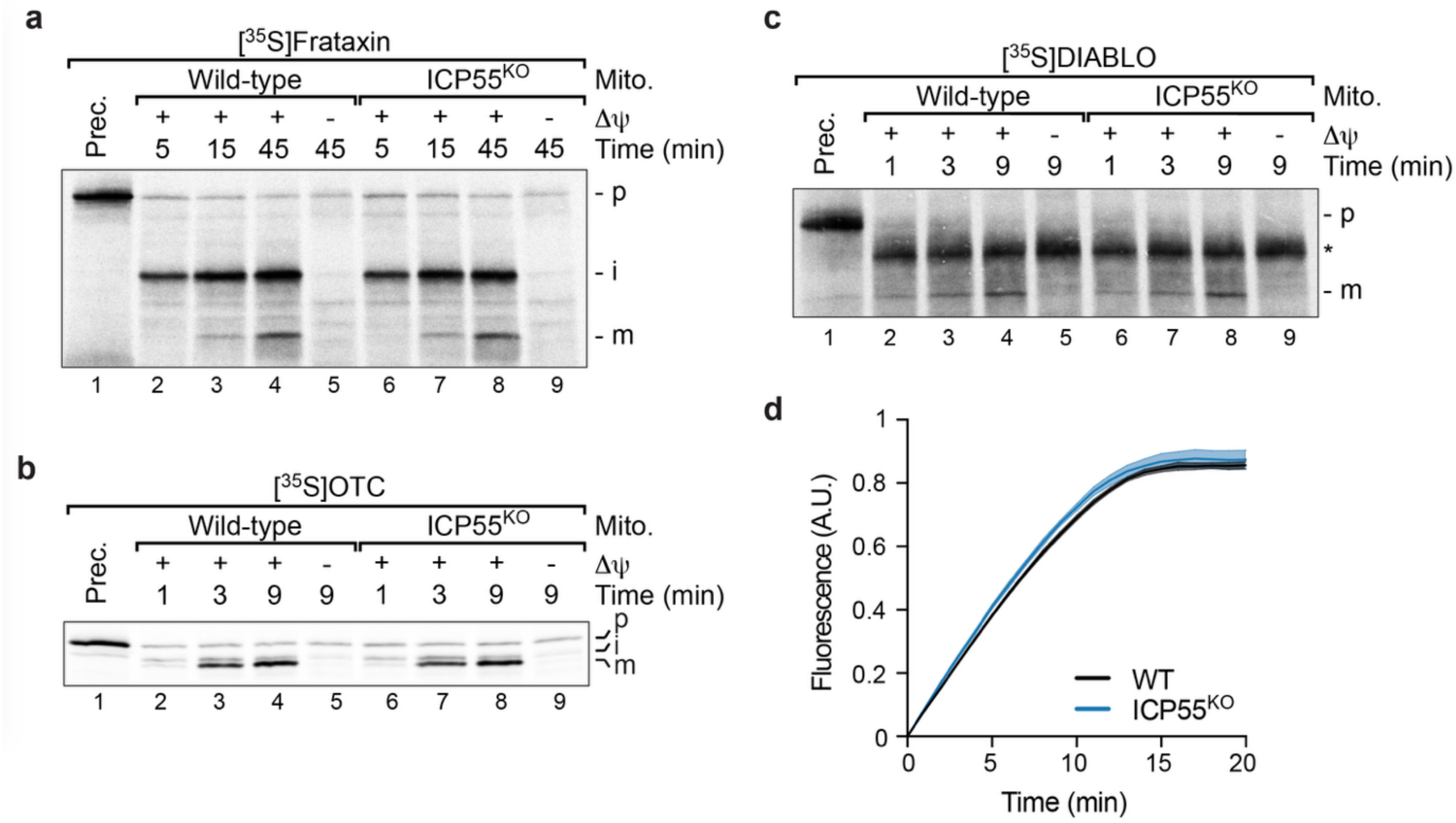

New Figure R3. a-c) *In organello* import of radiolabeled precursor proteins into isolated wild-type (WT) and ICP55^{KO} mitochondria. Where indicated the membrane potential ($\Delta\psi$) was disspiated prior to the import reaction. p, precursor; i, intermediate; m, mature protein. d) A fluorogenic PreP substrate was added to WT and ICP55^{KO} soluble mitochondrial extract. Fluorescence intensity was measured over time (excitation 320 nm, emission 405 nm). All experiments were performed in three biological replicates with the same results.

<Minor>
In the current manuscript, it is difficult to directly connect the data presented in the Extended Data Tables with the figures and results discussed in the main text. Explicit citation of the relevant Extended Data Tables in the main text and figure legends would greatly improve clarity and facilitate further evaluation of the data and manuscript.

We thank the reviewer for this helpful suggestion and apologize for the lack of clarity in the presentation of the Extended Data Tables. We have carefully revised the manuscript to ensure explicit and consistent citation of all relevant Extended Data Tables throughout the main text and figure legends. In addition, we have updated the table descriptions and organization to improve clarity and facilitate direct cross-referencing between datasets, figures, and results.

Reviewer #3:
The manuscript investigates the role of the mitochondrial intermediate cleaving peptidase ICP55 in human cells. Using HUNTER N-terminomics in HEK293T mitochondria, the authors map 446 processed mitochondrial N-termini and identify 107 high-confidence ICP55 substrates, extending known motifs beyond canonical Y/L/F to include R and Q at position 1. Quantitative proteomics shows relatively modest changes in steady-state protein abundance but increased levels of specific assembly factors (SDHAF2, COX7A2L). SILAC-based complexome profiling of mixed WT/ICP55-/- mitochondria indicates that many inner-membrane and matrix proteins shift from high-molecular-weight assemblies to smaller

complexes or monomers, particularly in the ETC, mitoribosome and metabolic enzymes such as IDH3. The authors conclude that ICP55-mediated removal of a single N-terminal residue is a major determinant of mitochondrial complex integrity and suggest that defective N-terminal processing contributes to human mitochondrial disease and possibly age-associated decline. Overall, this is a very nice and interesting study, but several points need to be addressed.

We thank the reviewer for the positive evaluation of our manuscript.

Major points

1. The central mechanistic claim is that retention of a single N-terminal residue on ICP55 substrates directly destabilizes protein–protein interfaces and thereby impairs assembly and/or stability of complexes (ETC, HSP60, mitoribosome, IDH3). The data clearly support an association between ICP55 loss and decreased complex integrity, and HSP60 is a particularly convincing example. However, for most complexes the evidence remains correlative: steady-state levels of many ETC subunits are unchanged or only mildly altered, but supercomplex abundance is reduced, complexes are more urea sensitive, and OCR is decreased. These observations are fully consistent with impaired complex integrity but do not by themselves demonstrate that the one-residue extension on multiple subunits is the primary cause. There is no direct proof that incorporation of the unprocessed form is necessary and sufficient for destabilization. Rescue of ETC supercomplex stability and function by ICP55 re-expression, or targeted N-terminal mutants that mimic processed versus unprocessed states in a defined subunit, would substantially strengthen the proposed causal link. In the absence of such data, the text should more carefully distinguish between "consistent with" and "demonstrates that" when attributing destabilization specifically to the extra residue.

We thank the reviewer for this important and insightful comment regarding the mechanistic basis of complex destabilization. We agree that establishing a causal link between retention of an additional N-terminal residue and impaired complex stability is critical. To directly address this point, we have performed two new sets of experiments.
First, we tested whether re-expression of ICP55 can rescue the observed defects in mitochondrial complex integrity and function. We find that ICP55 re-expression restores respiratory chain supercomplex abundance and oxygen consumption (**new Extended Data Fig. 4c and Fig. 2i**), demonstrating that the observed phenotypes are specifically caused by loss of ICP55 activity.

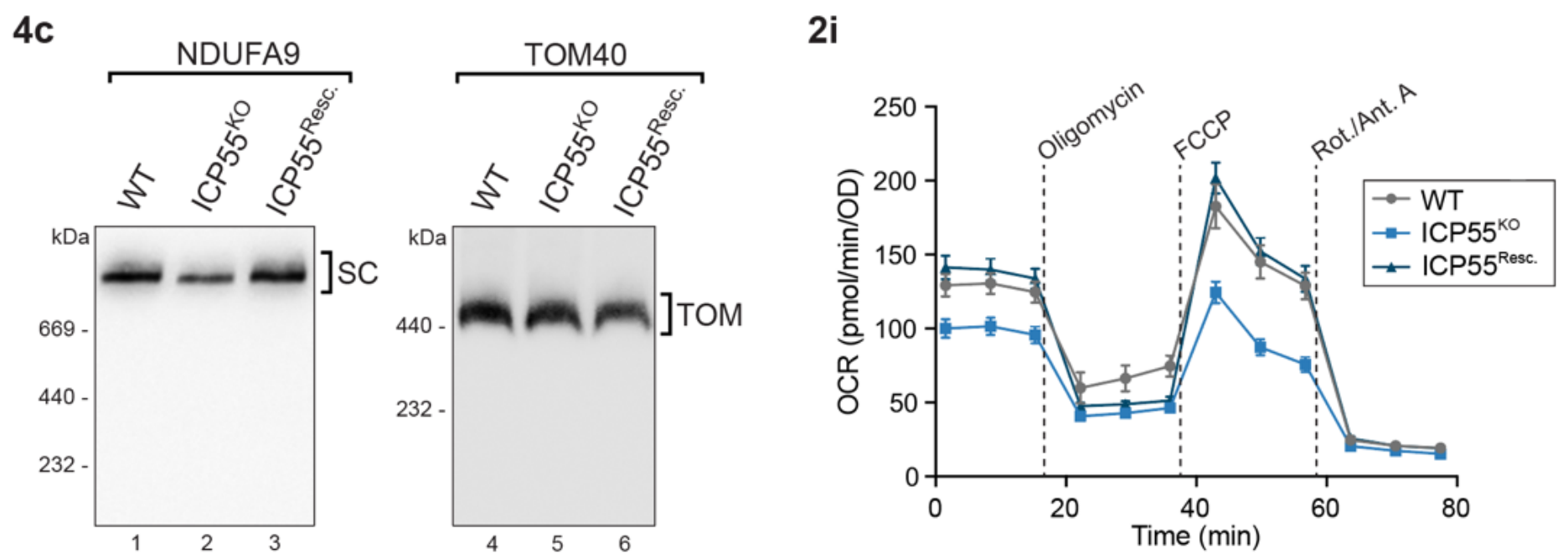

New Extended Data Figure 4c and Figure 2i. 4c) Isolated mitochondria from WT, ICP55^{KO} and ICP55^{KO} cells re-expressing ICP55 (ICP55$^{Resc.}$) were solubilized in digitonin buffer and analysed by BN-PAGE and immunoblotting. SC, respiratory chain supercomplexes; TOM40, loading control. 2i) Analysis of oxygen consumption rates (OCR) of WT, ICP55^{KO} and ICP55$^{Resc.}$ cells under basal conditions and after additions of indicated compounds. Data represent mean ± SEM from six replicates. Rot., rotenone; Ant. A, antimycin A

Second, to directly assess the effect of a single additional N-terminal residue on protein complex stability, we established an *in vitro* system using HSP60. We expressed and purified HSP60 variants differing only in their N-terminal residue (either Tyr, representing the processing intermediate, or Ala, representing the mature form). Following ATP-induced assembly (Weiss et al., 2024), we analyzed complex stability by BN-PAGE and size exclusion chromatography. While the mature HSP60 remained stably assembled, the variant carrying the additional N-terminal tyrosine rapidly disassembled, as evidenced by reduced oligomeric species and the appearance of monomeric forms (**new Extended Data Fig. 10** and **new Fig. 6b-f**).

These results provide direct experimental evidence that the presence of a single additional N-terminal residue is sufficient to destabilize a mitochondrial protein complex. We have incorporated these new data into the revised manuscript and updated the corresponding Results and Discussion sections accordingly. In particular, we have refined the text to more clearly delineate experimentally demonstrated effects from broader observations on mitochondrial complex integrity.

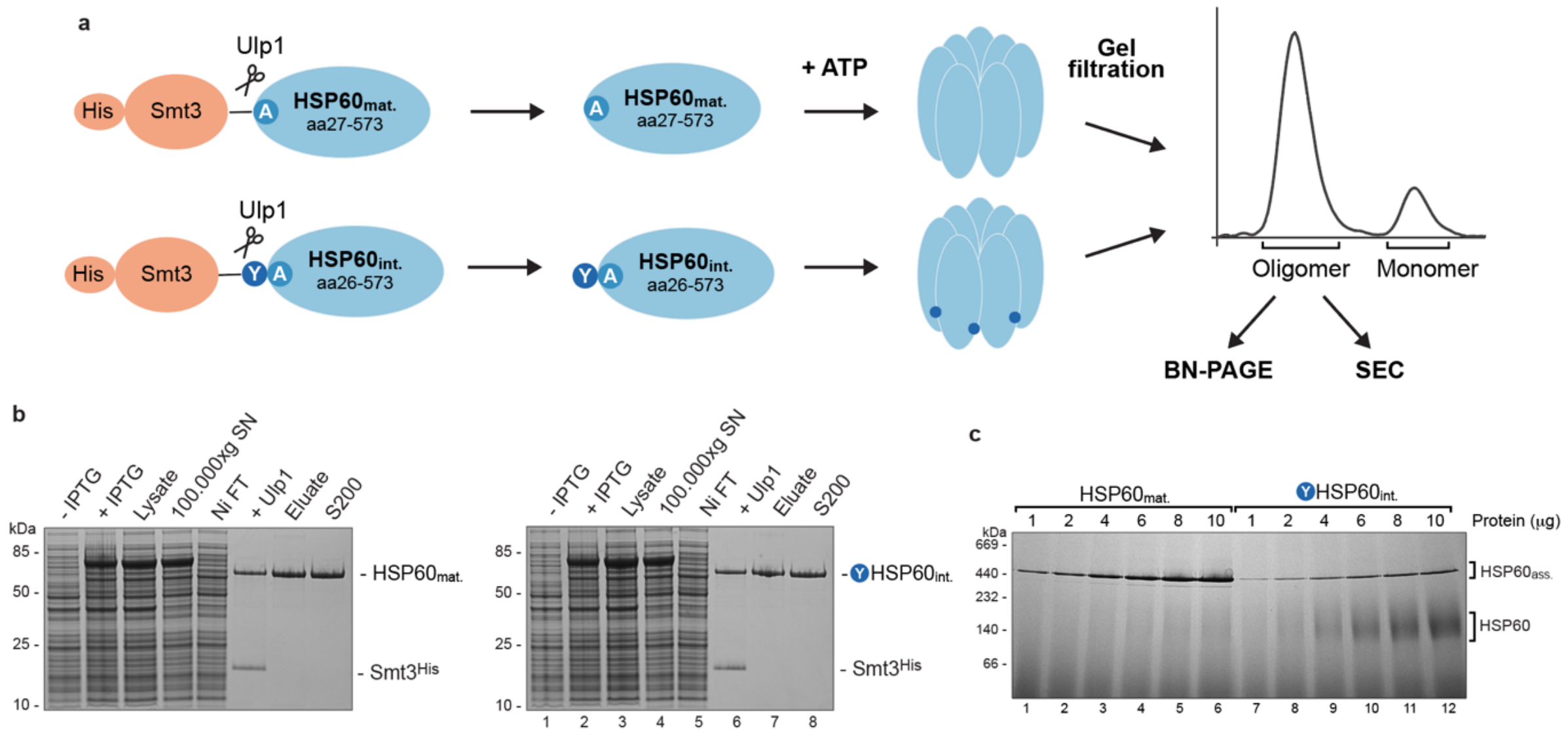

New Extended Data Figure 10. a) Schematic representation of the purification and *in vitro* assembly strategy. b) Expression and purification from HSP60 (mature form starting with Ala and intermediate form starting with Tyr) from *E. coli*. Expression was induced by addition of IPTG. The bacterial lysate was cleared by centrifugation at 100.000g, and the supernatant was applied to a NiNTA column. Bound HisSmt3-HSP60 was cleaved by incubation with Ulp1-GST and the released protein was cleared over a GSH agarose column. The flowthrough containing HSP60 was incubated with ATP and separated by size exclusion chromatography (SEC; S200 column). Samples representing the different steps of the purification procedure were analyzed by SDS-PAGE and Coomassie staining. c) Oligomeric HSP60 (mature and intermediate form) were analyzed on BN-PAGE and proteins visualized by Coomassie staining. $HSP60_{ass.}$, assembled HSP60 oligomer.

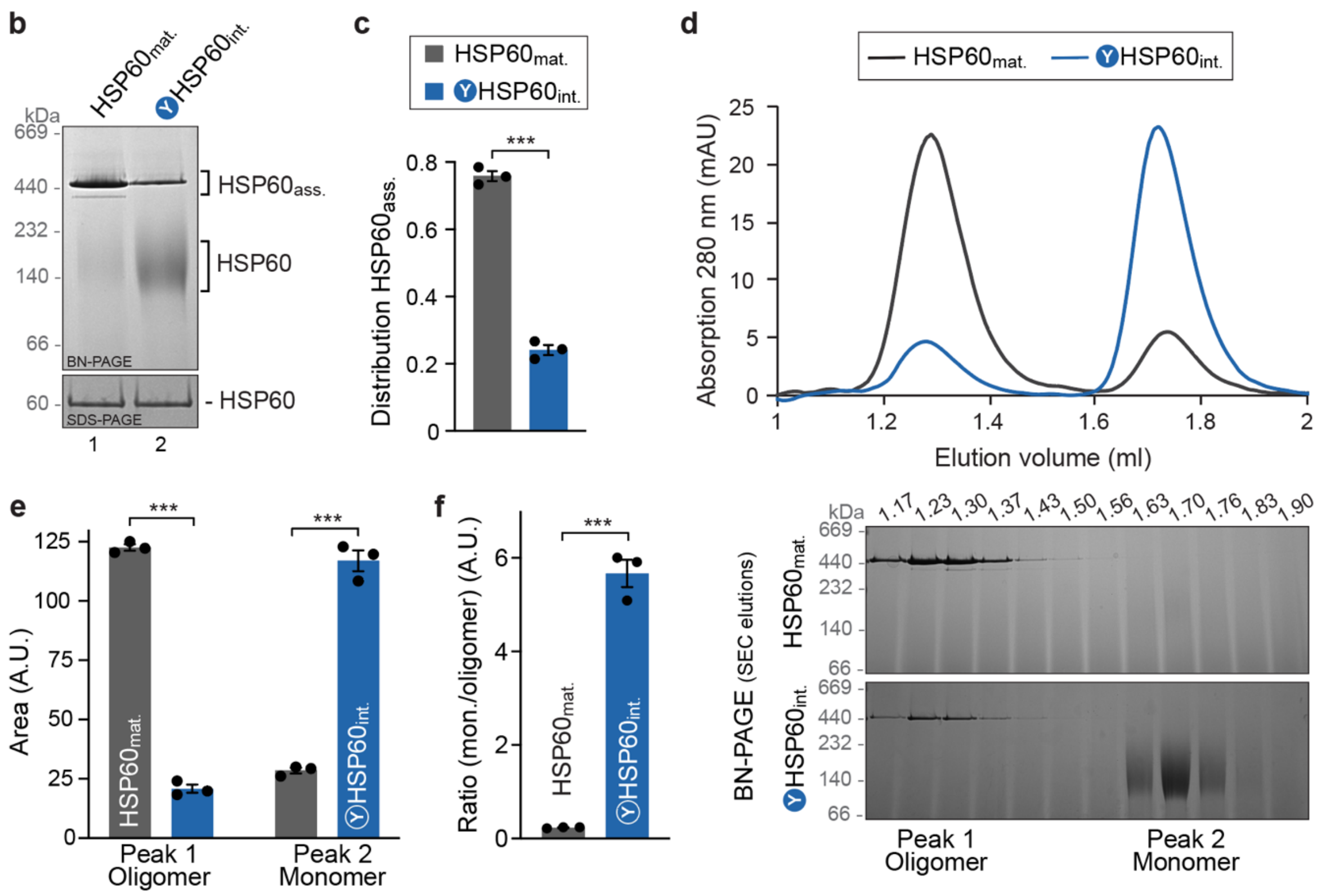

New Figure 6b-f. b) Analysis of purified mature and intermediate HSP60. Samples were analysed on BN-PAGE (top panel) or subjected to SDS-PAGE (lower panels). Proteins were visualized by Coomassie staining. $HSP60_{ass.}$, assembled HSP60 barrel. c) Quantification of assembled HSP60 from b). Data represent mean ± SEM from three replicates. Student's t test was used to compare between two groups. Significances are indicated with asterisks: ***$p < 0.001$. d) Analysis of mature (mat.) and intermediate (int., Y) HSP60 oligomer by size exclusion chromatography. Purified recombinant HSP60 oligomer was applied to a S200 Increase 5/150 GL column and eluting protein species were monitored at 280 nm (chromatogram, upper panel) and analyzed by BN-PAGE and Coomassie staining (lower panel). e) The experiment was performed as in d) using less protein and monitoring eluting protein species at 205 nm. Quantification of peak areas of the corresponding chromatograms (as depicted in d)) representing the oligomer (peak 1, at around 1.27 ml) and the monomer (peak 2, at around 1.72 ml). Data represent mean ± SEM from three replicates. f) Ratio of monomeric (mon.) and oligomeric form of mature and intermediate HSP60 from analysis in d). Data represent mean ± SEM from three replicates.

2. The manuscript repeatedly describes ICP55 as a central determinant of global mitochondrial complex integrity and a key stabiliser of multimeric mitochondrial protein complexes. While the complexome data clearly show widespread effects in ICP55-/- mitochondria, only a subset of mitochondrial proteins (177/933) display significantly altered migration, and the magnitude of changes differs across complexes. A more accurate and defensible wording would emphasize a broad or widespread impact, particularly on matrix and inner-membrane assemblies, rather than implying near-universal dependence of all complexes on ICP55. Along the same lines, the estimate that around 300 of about 1100 mitochondrial proteins are ICP55 substrates is extrapolated from 107 experimentally validated substrates in HEK293T plus in silico predictions that are acknowledged to be incomplete. This is reasonable as an upper-bound estimate but should be clearly labeled as such, not phrased as a firm number. Likewise, the proposed contribution of ICP55 defects to human disease and ageing-related mitochondrial decline is interesting and plausible, but remains hypothetical at this stage. Therefore, the Discussion should consistently frame these as well-supported hypotheses that require validation in patient-derived cells or in vivo models, rather than as established mechanisms.

We thank the reviewer for this important comment. We have revised the manuscript and specifically now describe the effects of ICP55 loss as broad, with a particular impact on mitochondrial matrix and inner membrane protein complexes. In addition, we have clarified that the estimate of appr. 300 ICP55 substrates represents an upper-bound approximation based on the combination of experimentally identified substrates and *in silico* predictions, rather than a definitive number.
Finally, we have revised the Discussion to consistently frame the potential contribution of ICP55 dysfunction to human disease and ageing-related mitochondrial decline as a hypothesis that requires further validation in patient-derived cells or *in vivo* models, rather than as an established mechanism.

3. The "dis-elution score" approach based on Manhattan distances is reasonable in principle, but the statistical treatment is underdescribed and appears lenient. Significant alterations are called at $p \leq 0.05$ across 933 mitochondrial proteins, without an explicit correction for multiple testing. At this scale, a substantial number of false positives is expected by chance. For a study that builds key conclusions on a global shift in complex architecture, it is important either to apply a multiple-testing correction (for example an FDR procedure such as Benjamini–Hochberg) or to explicitly quantify the expected false discovery rate and discuss it. In addition, more quantitative summary metrics (for example changes in OSR distributions, or the fraction of total signal in high- versus low-molecular-weight bins in WT versus ICP55-/-) would more robustly support the claim of a proteome-wide bias toward smaller assemblies. When commenting on statistical approaches, one further point is that essentially all western blots in the manuscript need to be repeated with proper biological replicates and quantified. Single 1+1 blots without quantification are not acceptable as evidence for abundance differences.

We thank the reviewer for this important comment and for highlighting the need for a more rigorous statistical framework.
To address this concern, we implemented two complementary approaches: (i) multiple testing correction using the Benjamini-Hochberg (BH) procedure and (ii) an empirical permutation-based analysis to estimate the expected false discovery rate and to assess the global significance of the observed effect. First, applying BH correction across all tested proteins reduced the number of significant proteins from 177 ($p \leq 0.05$) to 115 at an adjusted FDR threshold of ≤ 0.05, demonstrating that a substantial fraction of the hits remains robust after controlling for multiple testing. Second, to directly estimate the expected number of false positives and evaluate the global significance of the observed shift, we performed an exact permutation analysis (**new Fig. R4**). Because the dataset consists of four WT and four ICP55^{KO} elution profiles, all possible assignments of profiles to the two conditions were enumerated ($n = 70$). For each permutation, the complete analysis pipeline was rerun. This analysis revealed that, under random label assignments, an average of 30.5 (raw p-value) and 8.5 (BH-adjusted) proteins are detected as significant, compared to 177 and 115 proteins in the real dataset, respectively. This corresponds to an empirical false discovery rate of appr. 17% (raw p-values) and appr. 7% (BH-adjusted), indicating that the majority of detected proteins represent true signal. Importantly, the number of significant proteins observed in the real dataset exceeded those obtained in permuted datasets in nearly all cases (66/69), resulting in an empirical global p-value of 0.0435. This demonstrates that the observed proteome-wide shift in elution behavior is unlikely to arise from random variation. Together, these analyses show that while some false positives are expected at this scale, the overall effect is robust and statistically significant, supporting our conclusion of a widespread alteration in protein complex organization.

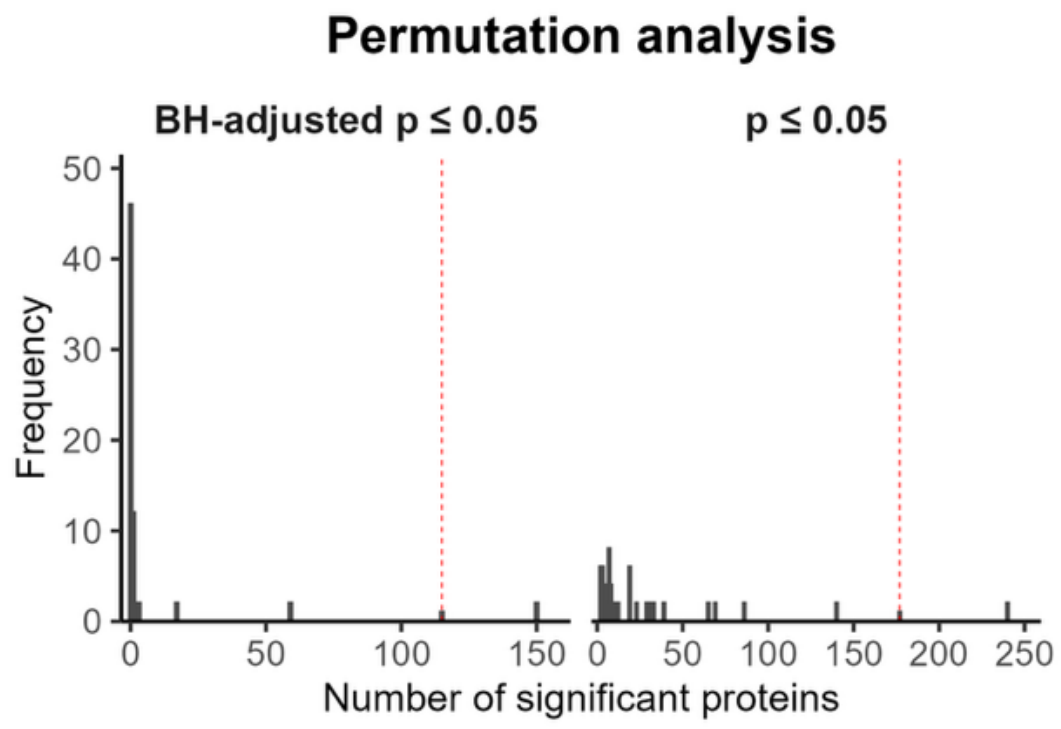

New Figure R4. Exact permutation analysis of complexome profiles (4 WT, 4 ICP55^{KO}; n = 70 permutations) estimating expected numbers of significant proteins under random label assignment. Comparison of observed versus permuted datasets demonstrates a robust, statistically significant shift in protein complex distribution (empirical p = 0.0435), with controlled false discovery rates after Benjamini-Hochberg (BH) correction.

We also agree with the reviewer that quantitative summary metrics are important to support the conclusion of a global shift toward smaller assemblies. In our manuscript, this aspect is addressed in Figure 5, which provides a global and quantitative overview of the observed changes:

- In Figure 5a, we show that the first peak of the elution profile - typically corresponding to the fraction of a protein within high-molecular-weight complexes - exhibits reduced abundance in the mutant. A similar trend is observed for the majority of second peaks.
- In Figure 5b, we demonstrate that the mutant condition displays additional elution peaks. These peaks are predominantly associated with low OSR values, indicating redistribution of proteins from high-molecular-weight assemblies into smaller complexes or monomeric forms.
- Finally, Figures 5h and 5i show that the mutant exhibits a higher number of unique peaks in later elution fractions (peaks 3-6), corresponding to lower molecular weight regions.

Together, these analyses provide a quantitative, proteome-wide view, supporting our conclusion that ICP55 deletion causes destabilization of protein complexes and a shift of protein subunits from larger assemblies toward smaller complexes.
Furthermore, we now provide quantification of all western blots and autoradiography scans from independent biological replicates directly in the main figures. This includes quantitative analysis of HSP60 assembly and stability (with and without urea treatment; **new Fig. 3b,c,f,g**). In addition, the quantitative proteome (n=5 biological replicates) and complexome profiling (n=4 biological replicates) provide independent and consistent quantitative readouts, all of which are in agreement and support our conclusions.

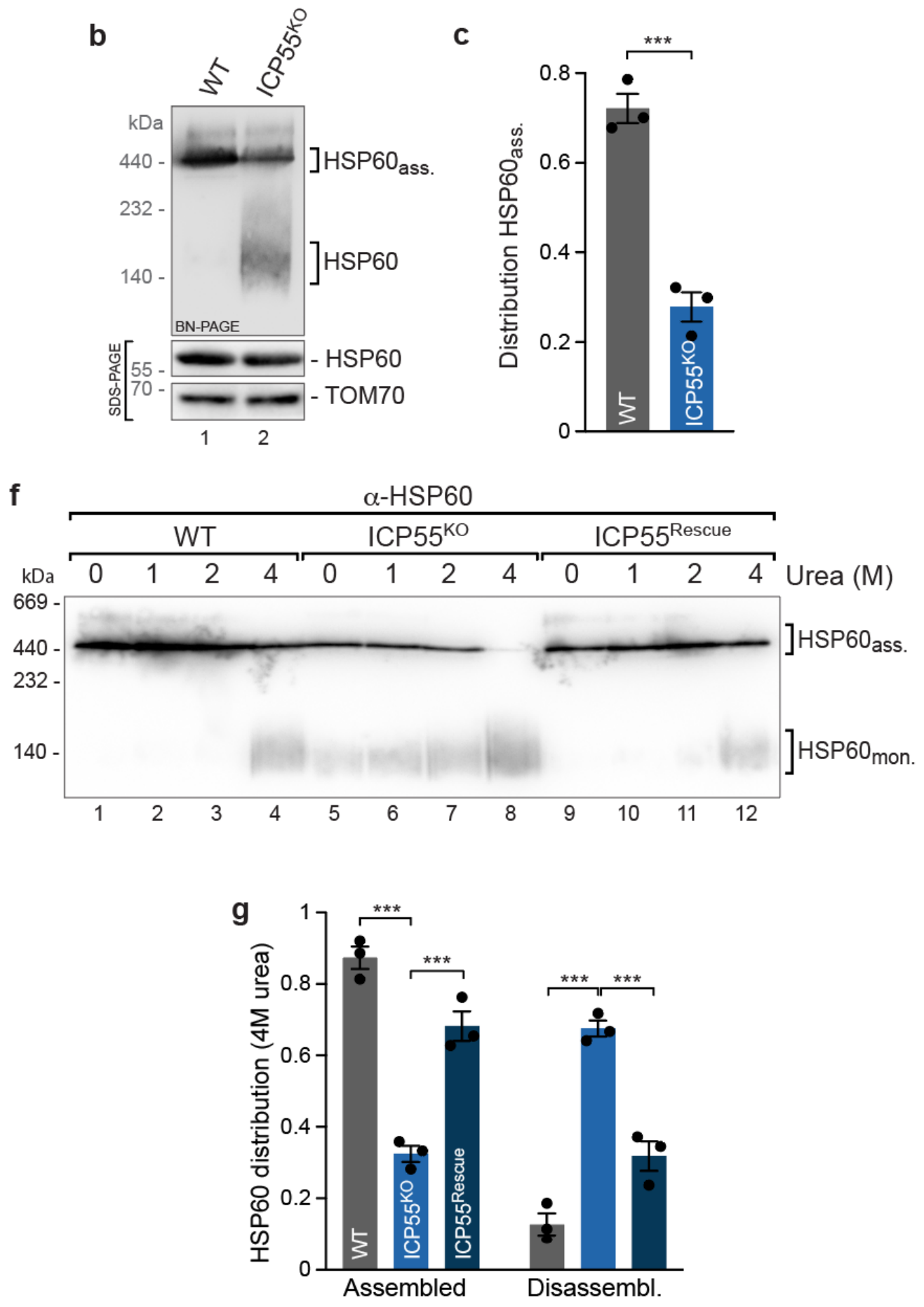

New Figure 3b,c,f,g. b) Analysis of protein levels in wild-type (WT) and ICP55^{KO} mitochondria. Samples were solubilized in digitonin buffer and analysed on BN-PAGE (top panel) or subjected to SDS-PAGE (lower panels) followed by immunoblotting. HSP60$_{ass.}$, assembled HSP60 barrel. TOM70, loading control. c) Quantification of assembled HSP60 from b). Data represent mean ± SEM from three replicates. Student's t test was used to compare between two groups. Significances are indicated with asterisks: ***$p < 0.001$. f) Isolated mitochondria from WT, ICP55^{KO} and ICP55^{KO} cells re-expressing ICP55 were solubilized in digitonin buffer in the absence or presence of different concentrations of urea and analysed by BN-PAGE and immunoblotting. g) Quantification of assembled and disassembled HSP60 after treatment of isolated mitochondria with 4 M urea (analysis as in f). Data represent mean ± SEM from three replicates. Statistical significance was assessed by one-way ANOVA followed by Tukey's multiple-comparison test, performed separately for assembled and disassembled fractions.

4. The BN-PAGEs in Figure 2 are not of sufficient quality. The supercomplex region appears collapsed into essentially one broad band, suggesting that the gels have not been run long enough to resolve respiratory supercomplexes adequately. These experiments should be repeated under conditions that provide proper separation. In addition, the quantification of BN-PAGEs in Figures 2d and 2g is problematic: control values are all normalised to 1 and thus show no variation, which is not an appropriate way to represent statistics across biological replicates.

We have repeated the BN-PAGE analyses under optimized conditions to improve resolution of respiratory supercomplexes. We now obtain clear separation of multiple supercomplex species, although their visibility depends on exposure conditions. Longer exposures reveal additional supercomplex bands, whereas shorter exposures better preserve the dynamic range of the most abundant species. To provide a balanced representation, we selected exposure conditions in the revised manuscript that allow visualization of multiple supercomplex bands while maintaining quantitative detectability of differences between WT and ICP55^{KO} mitochondria (**new Fig. 2c**).
In addition, we have revised the quantification of BN-PAGE data to appropriately reflect variability across biological replicates. Specifically, WT values are no longer fixed to 1 but instead display their inherent variation, enabling proper statistical comparison (**new Fig. 2e,h**).

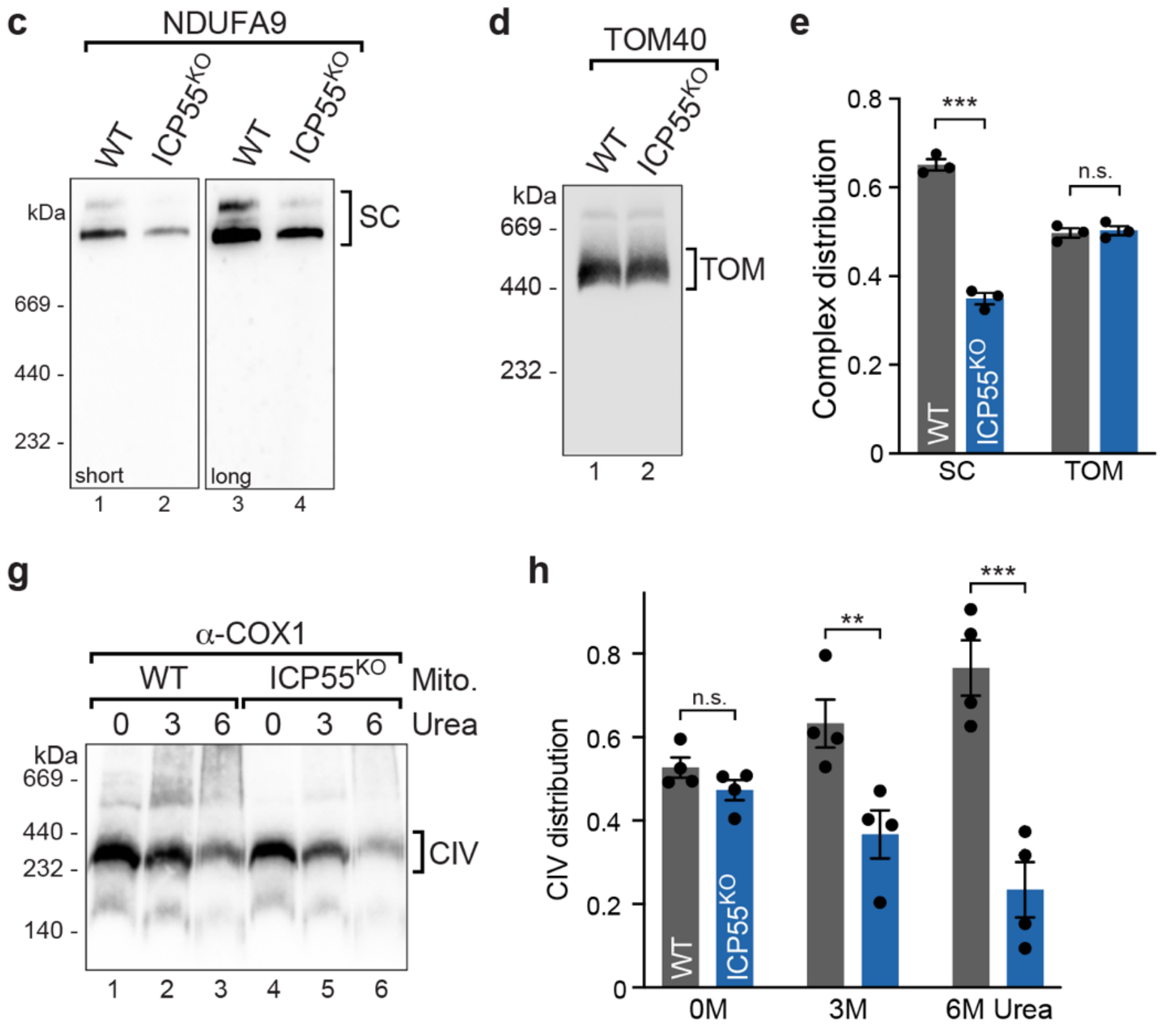

New Figure 2. c-d) Isolated mitochondria from WT and ICP55^{KO} cells were solubilized in digitonin buffer and analysed by BN-PAGE and immunoblotting. SC, respiratory chain supercomplexes; TOM, translocase of the outer mitochondrial membrane; short, short exposure; long, long exposure. e) Quantification of supercomplex and TOM complex from analysis in (c and d) using antisera against Rieske (CIII subunit) and TOM40. Data represent mean ± SEM from three replicates. Student's t test was used to compare between two groups. Significances are indicated with asterisks: ***$p < 0.001$; n.s., not significant. g) Determination of ETC complex IV stability by addition of 3 and 6 M urea during digitonin solubilization. Samples were analysed as in (c). h) Quantification of samples from (g). Data represent mean ± SEM from four replicates. Student's t test was used to compare between two groups. **$p < 0.01$.

Point-by-point response

Reviewer #1:
Remarks to the Author:
The authors have responded to my previous concerns in an adequate manner. Publication of this interesting manuscript can now be recommended.

We thank the reviewer for the positive comment on our revised manuscript.

Reviewer #2:
Remarks to the Author:
I have carefully evaluated the revised manuscript and the authors' point-by-point response. The authors have addressed my previous concerns and the manuscript has been substantially improved. Specifically, the addition of the new protease activity assays (evaluating MPP, MIP, IMP, and PreP) convincingly demonstrates that the observed complexome destabilization is specifically due to ICP55 deficiency, rather than a broad, non-specific impairment of mitochondrial proteases. Furthermore, the reorganization of Extended Data Table 3 to include submitochondrial localization annotations greatly improves the clarity and accessibility of the 107-substrate dataset.

Given the technical rigor and the conceptual advance presented in this revised version, I recommend the acceptance of this manuscript for publication in Nature Structural & Molecular Biology.

We are grateful to the reviewer for the positive evalution of our revised manuscript.

Reviewer #3:
Remarks to the Author:
I thank the authors for their careful and satisfactory revision. The manuscript has been substantially strengthened, and my main concerns have been appropriately addressed. Most importantly, the new ICP55 re-expression experiment provides a key causal control. The rescue of respiratory chain supercomplex abundance and oxygen consumption strongly supports the conclusion that the observed mitochondrial defects are specifically caused by loss of ICP55. This addition considerably improves the mechanistic strength of the study. Overall, the authors have addressed my concerns in a detailed and convincing manner.

We are happy that the additional experiments provided in the revised version are positively evaluated by the reviewer and thank the reviewer for the positive feedback.
